# CDKL5 kinase controls transcription‐coupled responses to DNA damage

**DOI:** 10.15252/embj.2021108271

**Published:** 2021-10-04

**Authors:** Taran Khanam, Ivan Muñoz, Florian Weiland, Thomas Carroll, Michael Morgan, Barbara N Borsos, Vasiliki Pantazi, Meghan Slean, Miroslav Novak, Rachel Toth, Paul Appleton, Tibor Pankotai, Houjiang Zhou, John Rouse

**Affiliations:** ^1^ MRC Protein Phosphorylation and Ubiquitylation Unit School of Life Sciences University of Dundee Dundee UK; ^2^ Albert Szent‐Györgyi Medical School Institute of Pathology University of Szeged Szeged Hungary; ^3^ MRC Reagents and Services School of Life Sciences University of Dundee Dundee UK; ^4^ Dundee Imaging Facility School of Life Sciences University of Dundee Dundee UK; ^5^ Present address: Department of Microbial and Molecular Systems (M²S) Centre for Food and Microbial Technology (CLMT) Laboratory of Enzyme, Fermentation and Brewing Technology (EFBT) Technology Campus Ghent, KU Leuven Ghent Belgium; ^6^ Present address: Department of Biochemistry and Biophysics University of California San Francisco CA USA; ^7^ Present address: Department of Medical Genetics National Health Service, Polwarth Building Foresterhill UK; ^8^ Present address: Jacqui Wood Cancer Centre Ninewells Hospital University of Dundee Dundee UK; ^9^ Present address: Bioscience Core Laboratory King Abdullah University of Science and Technology Thuwal Saudi Arabia

**Keywords:** CDKL5 disorder, DNA damage response, kinase, poly(ADP‐ribose), transcriptional regulation, DNA Replication, Recombination & Repair, Post-translational Modifications & Proteolysis, Proteomics

## Abstract

Mutations in the gene encoding the CDKL5 kinase are among the most common genetic causes of childhood epilepsy and can also give rise to the severe neurodevelopmental condition CDD (CDKL5 deficiency disorder). Despite its importance for human health, the phosphorylation targets and cellular roles of CDKL5 are poorly understood, especially in the cell nucleus. Here, we report that CDKL5 is recruited to sites of DNA damage in actively transcribed regions of the nucleus. A quantitative phosphoproteomic screen for nuclear CDKL5 substrates reveals a network of transcriptional regulators including Elongin A (ELOA), phosphorylated on a specific CDKL5 consensus motif. Recruitment of CDKL5 and ELOA to damaged DNA, and subsequent phosphorylation of ELOA, requires both active transcription and the synthesis of poly(ADP‐ribose) (PAR), to which CDKL5 can bind. Critically, CDKL5 kinase activity is essential for the transcriptional silencing of genes induced by DNA double‐strand breaks. Thus, CDKL5 is a DNA damage‐sensing, PAR‐controlled transcriptional modulator, a finding with implications for understanding the molecular basis of CDKL5‐related diseases.

## Graphical abstract

Phosphoproteomics identification of nuclear CDKL5 substrates implicates the neurodevelopmental disease‐linked kinase in poly(ADP‐ribose)‐mediated silencing at DNA double‐strand breaks.

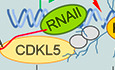

## Introduction

Cyclin‐dependent kinase‐like 5 (CDKL5) is a poorly characterized protein kinase, which is mutated in a rare, debilitating condition known as CDKL5 deficiency disorder (CDD; OMIM 300203; 300672) (Kalscheuer *et al*, 2003; Fehr *et al*, 2013). In particular, CDD is characterized by seizure onset usually before 3 months of age, severe neurodevelopmental delays, grievously impaired motor, language and hand skills, cortical visual impairment and other symptoms (Fehr *et al*, 2013). Current treatments for CDD focus on the management of symptoms, not the underlying cause of the disease. Although CDD is rare, it was recently discovered that *CDKL5* is one of the most commonly mutated genes in childhood epilepsy, and *CDKL5* mutations have also been associated with milder syndromes typified by intellectual disability and behavioural defects (Krishnaraj *et al*, 2017; MacKay *et al*, 2020). Thus, the prevalence of *CDKL5* mutations is much higher than thought previously. Developing rational therapies to treat the root cause of CDKL5‐related diseases requires an understanding of the molecular basis of these diseases and the basic functions of CDKL5. However, at present the phosphotargets and cellular roles of this kinase are poorly understood. Identifying the cellular targets of CDKL5 is crucial because the CDD‐associated mutations strongly reduce kinase activity (Munoz *et al*, 2018), suggesting that the reduced phosphorylation of CDKL5 target proteins causes brain dysfunction and disease.

Recently, we and others described complementary substrate screens to identify physiological targets of CDKL5 (Baltussen *et al*, 2018; Eyers, 2018; Munoz *et al*, 2018). These efforts revealed a network of microtubule and centrosome regulators phosphorylated by CDKL5, including MAP1S, CEP131, ARHGEF2, EB2 and DLG5. The phosphorylated serine in all of these targets lies in a common motif: R‐P‐X‐S‐A. Experiments with synthetic peptides corresponding to the sequence around the CDKL5 phosphorylation site Ser^900^ in MAP1S provided information on CDKL5 specificity. Whereas substitution of the R and P residues upstream of the phospho‐acceptor S abolishes phosphorylation by CDKL5, a G or P residue at the +1 position could be accommodated instead of the A; furthermore, T can be accommodated as the phospho‐acceptor residue (Munoz *et al*, 2018). These data suggested that the motif R‐P‐X‐[S/T]‐[A/G/P] represents a putative CDKL5 consensus motif.

The microtubule‐associated substrates of CDKL5 are based in the cytoplasm. However, CDKL5 is also located in the nucleus but little is known about its functions in this compartment, and nuclear phosphotargets were conspicuously absent from the published screens (Baltussen *et al*, 2018; Munoz *et al*, 2018). A recent report found that CDKL5 promotes renal injury in mice exposed to toxic insults by upregulating SOX9‐dependent genes (Kim *et al*, 2020). This role was linked to phosphorylation of SOX9, but the phosphoserine reported to be phosphorylated by CDKL5 (Q‐T‐H‐I‐phospho‐S^199^‐P) does not lie in an R‐P‐X‐[S/T]‐[A/G/P] motif and is therefore unlikely to be a direct CDKL5 target. Thus, there is an urgent need to identify CDKL5 targets in the nucleus to help understand its roles in this cellular compartment.

Protein kinases in the nucleus play vitally important roles in the sensing, signalling and repair of DNA damage, in particular the related kinases ATM, DNA‐PK and ATR (Jette & Lees‐Miller, 2015; Blackford & Jackson, 2017). These kinases transduce DNA damage signals, triggering a pleiotropic series of protective reactions collectively known as the DNA damage response (DDR), which prevents genome instability (Marechal & Zou, 2013; Shiloh & Ziv, 2013; Blackford & Jackson, 2017). Mutations in these kinases cause severe DNA repair defects and diseases typified by rampant genome instability including ataxia telangiectasia (ATM) and Seckel syndrome (ATR) (Blackford & Jackson, 2017). Although other kinases have been implicated in the DDR such as CHK1, CHK2, p38, JNK, MK2 and others (Smith *et al*, 2020), the overall proportion of the 560+ kinases in the human kinome (and the dark kinome (Berginski *et al*, 2021)) implicated in the DDR is low, and we speculated there may be more, perhaps with relevance to human diseases.

With the aim of expanding the repertoire of DDR kinases, we started screening the human kinome for kinases that are recruited to DNA damage sites. Here, we demonstrate that CDKL5 is recruited to DNA breaks in actively transcribed regions of the nucleus in a manner that requires the synthesis of poly(ADP‐ribose) (PAR), to which CDKL5 can bind. We present the results of a screen to identify nuclear targets of CDKL5, including Elongin A (ELOA) whose recruitment and phosphorylation at Ser^311^ require PAR synthesis and transcriptional activity like CDKL5. CDD‐associated CDKL5 mutations severely reduced ELOA phosphorylation by CDKL5. Finally, we show that CDKL5 is required for silencing of transcription known to occur near DNA breaks.

## Results

### CDKL5 is recruited to damaged chromatin in a poly(ADP‐ribose)‐dependent manner

**Figure EV1:**
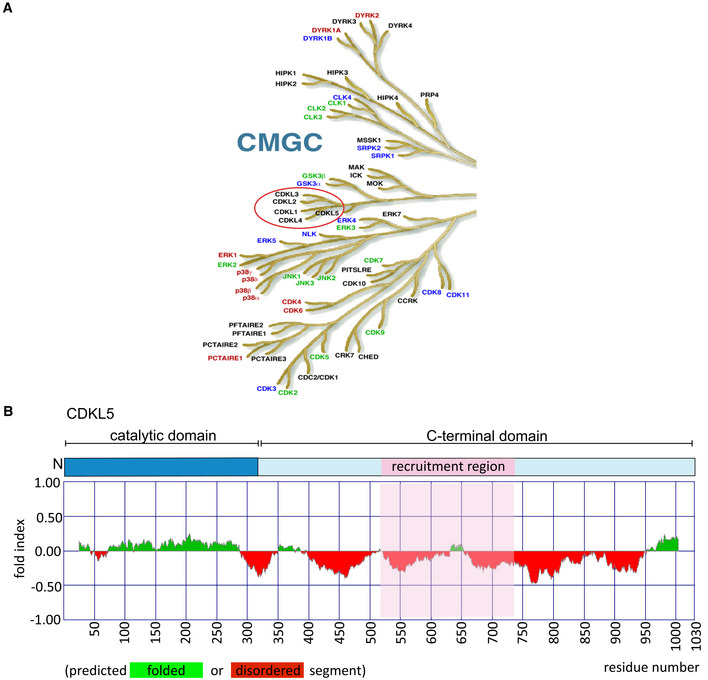
CDKL5 kinase The CMGC branch of the human kinome. The figure is taken from the dendrogram made by Manning and colleagues (Manning *et al*, 2002). The CDKL family of kinases that includes CDKL5 is encircled in red.(*Top*) Schematic diagram of CDKL5 protein. (*Bottom*) Bioinformatics analysis for CDKL5 folding using Fold index software (Prilusky *et al*, 2005). The plot shows the disorder prediction for CDKL5 protein sequence. Ordered regions are indicated in green above 0, while disordered regions are indicated in red below 0. Amino acids suggested as being folded or unfolded are depicted at the bottom of the plot. The region that mediates recruitment of CDKL5 to DNA damage sites is marked in pink.

Recruitment to DNA damage sites appears to be a universal feature of proteins regulating the vast range of protective responses encompassed by the cellular DDR (Aleksandrov *et al*, 2018). With the aim of expanding the repertoire of DDR kinases, we started screening the human kinome for kinases recruited to DNA damage sites. U–2–OS cells were stably transfected with an mCherry‐tagged form of the DNA repair nuclease FAN1 to mark DNA damage sites. These cells were transiently transfected with GFP‐tagged kinases individually, starting with the CMGC branch of the human kinome (Fig EV1A). After pre‐sensitizing with bromodeoxyuridine (BrdU), cells were micro‐irradiated using a 355‐nm laser along a track in the nucleus (“stripe”). We chose these conditions in particular in order to induce the most pleiotropic range of DNA lesions possible, to avoid restricting our screen to a particular type of DNA damage.

**Figure 1:**
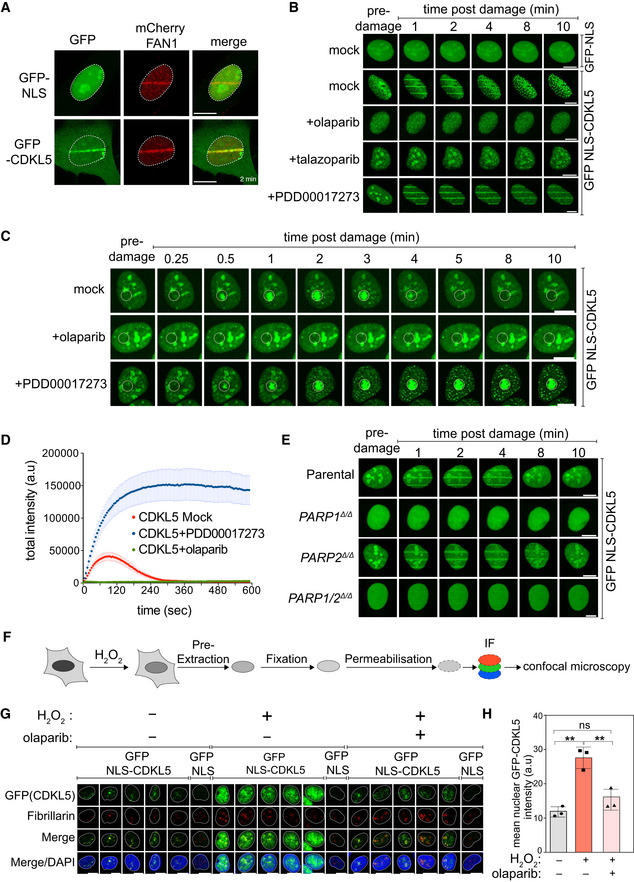
CDKL5 senses DNA damage in a PAR‐dependent manner BrdU‐sensitized U‐2‐OS Flp‐In T‐REx cells stably expressing mCherry‐FAN1 and GFP‐NLS or GFP–CDKL5 (no NLS) were line‐micro‐irradiated (355 nm) and imaged after 2 min. Scale bar is 10 μm.BrdU‐sensitized U‐2‐OS cells stably expressing GFP‐NLS‐CDKL5 were pre‐incubated with DMSO (mock), olaparib (5 μM), talazoparib (50 nM) or PDD00017273 (0.3 μM) for 1 h prior to micro‐irradiation and live imaged at the indicated times post–irradiation. One of three independent experiments is shown. Scale bar is 10 μm.Same as (B) except that cells stably expressing GFP‐NLS‐CDKL5 were pre‐incubated with DMSO (mock), olaparib (5 μM) or PDD00017273 (0.3 μM) for 1 h prior to spot micro‐irradiation (405 nm). Individual cells from one of two independent biological replicates are shown. Scale bar is 10 μm.Quantitation of spot intensities. Data represent the mean ± SEM of two independent experiments; > 50 micro‐irradiated cells per point.BrdU‐sensitized parental or *PARP1* ^Δ/Δ^, *PARP2* ^Δ/Δ^, *PARP1*/*2* ^Δ/Δ^ U–2–OS cells transiently expressing GFP‐NLS‐CDKL5 were subjected to 355 nm line micro‐irradiation followed by time‐lapse imaging. One of two independent experiments is shown. Scale bar is 10 μm.Diagram of the workflow for the chromatin retention experiments.Cells subjected to the workflow in (F) were detergent–extracted and fixed before staining with anti‐GFP or fibrillarin (nucleoli). Scale bar is 10 μm.Quantification of the detergent‐insoluble GFP‐NLS‐CDKL5 signal (minus nucleolar signal). The mean ± SD from three biological experiments is shown. Statistical significance was assessed by one‐way ANOVA test. Asterisks ** indicate *P*‐value of < 0.01; ns—not significant. Source data are available online for this figure.

The first GFP‐tagged kinase to demonstrate robust recruitment to sites of laser micro‐irradiation was CDKL5 (Fig 1A). Recruitment of GFP‐tagged CDKL5 to sites of line (Fig 1B) or spot (Fig 1C and D) micro‐irradiation was rapid and transient (Movies EV1 and EV2), reminiscent of proteins that bind poly(ADP‐ribose) chains generated by DNA damage‐activated poly(ADP‐ribose) polymerases (PARPs) (Ahel *et al*, 2009). Consistent with this idea, CDKL5 recruitment was blocked by the PARP inhibitors olaparib and talazoparib (Fig 1B–D) or by *PARP1* disruption (Fig 1E); in contrast, retention time was prolonged by PDD00017273, an inhibitor of poly(ADP‐ribose) glycohydrolase PARG, which delays PAR degradation (Fig 1B–D; Movies EV1 and EV2) (James *et al*, 2016). We took an alternative approach to visualize CDKL5 recruitment and found it to be retained on damaged chromatin after cells were exposed to H_2_O_2_, a potent inducer of DNA breaks and PARylation (Fig 1F–H). Retention of CDKL5 on chromatin induced by H_2_O_2_ was prevented by olaparib, whereas the nucleolar retention seen in undamaged cells was unaffected (Fig 1G and H).

**Figure 2:**
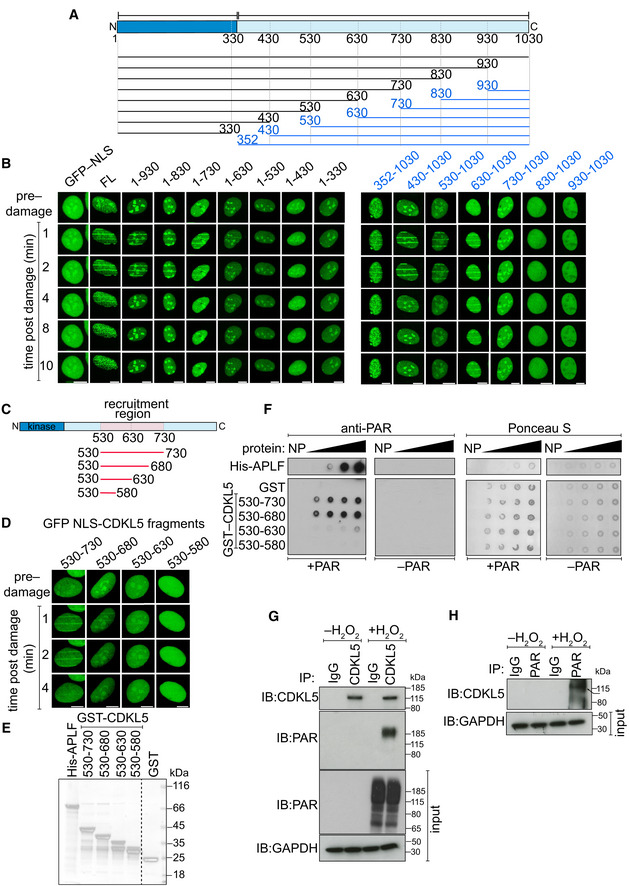
CDKL5 recruitment domain binds PAR directly ASchematic diagram of CDKL5 deletion mutants, deleting from the N‐terminal (blue) or C‐terminal (black) ends. All proteins were expressed with an N‐terminal NLS and GFP tag.BBrdU‐sensitized U‐2‐OS (Flp‐In T‐REx) cells stably expressing GFP‐NLS, the GFP‐NLS‐CDKL5 deletion mutants shown in (A) or full length (FL) GFP‐NLS‐CDKL5 was subjected to line micro‐irradiation (355 nm) and time‐lapse imaging. Three independent experiments were performed, and one representative experiment is shown. Scale bar is 10 μm.CSchematic for fragments corresponding to the PAR‐dependent recruitment region in CDKL5 as identified in (B).DSame as in (B) except that the GFP‐NLS‐tagged CDKL5 fragments indicated were examined. Scale bar is 10 μm.ECoomassie gel showing recombinant fragments of human CDKL5 fused to GST purified from bacterial lysates. GST and APLF were also purified as controls.FRecombinant fragments of CDKL5 fused to GST (1.2, 2.5, 5, 10 µg), or GST, were dot‐blotted on nitrocellulose membrane and then incubated with synthetic PAR. PAR binding was detected by far Western blotting. APLF was used as positive control. One of three independent experiments is shown.G, HU‐2‐OS (Flp‐In T‐Rex) cells stably expressing CDKL5 were either mock‐treated or treated with 500 µM H_2_O_2_ for 30 min. Extracts were subjected to immunoprecipitation with antibodies against CDKL5 (G) or PAR (H) (or non‐specific IgG as control). Precipitates, and input lysates, were analysed by Western blotting using the indicated antibodies. One of two independent experiments is shown. Source data are available online for this figure.

The data above indicate that CDKL5 recruitment to DNA breaks requires local synthesis of PAR. This may reflect a requirement for PAR‐mediated chromatin relaxation, or alternatively, CDKL5 may bind PAR directly. Using bioinformatics means, we failed to detect any of the known PAR‐binding motifs (Teloni & Altmeyer, 2016), and we therefore attempted to pinpoint the recruitment region in CDKL5 experimentally using a series of overlapping N‐terminal and C‐terminal deletion constructs. This revealed a region between amino acids 530 and 730 necessary for CDKL5 recruitment (Fig 2A and B); further deletions revealed that the region 530–680 of CDKL5 was sufficient (Fig 2C and D). This region is intrinsically disordered (Fig EV1B), reminiscent of proteins that undergo liquid de‐mixing when they bind PAR, such as FUS (Altmeyer *et al*, 2015). Recombinant CDKL5 fragments corresponding to the recruitment region pinpointed above bound to PAR *in vitro* (Fig 2E and F), albeit with a lower apparent affinity than the positive control APLF (Ahel *et al*, 2008). Accordingly, PAR was detected in CDKL5 precipitates, and vice versa, after exposure of cells to H_2_O_2_ (Fig 2G and H). Taken together, these data show that CDKL5 is recruited to DNA breaks in a manner requiring the synthesis of PAR, to which CDKL5 can bind directly.

### CDKL5 recruitment to DNA damage sites requires ongoing transcription

**Figure 3:**
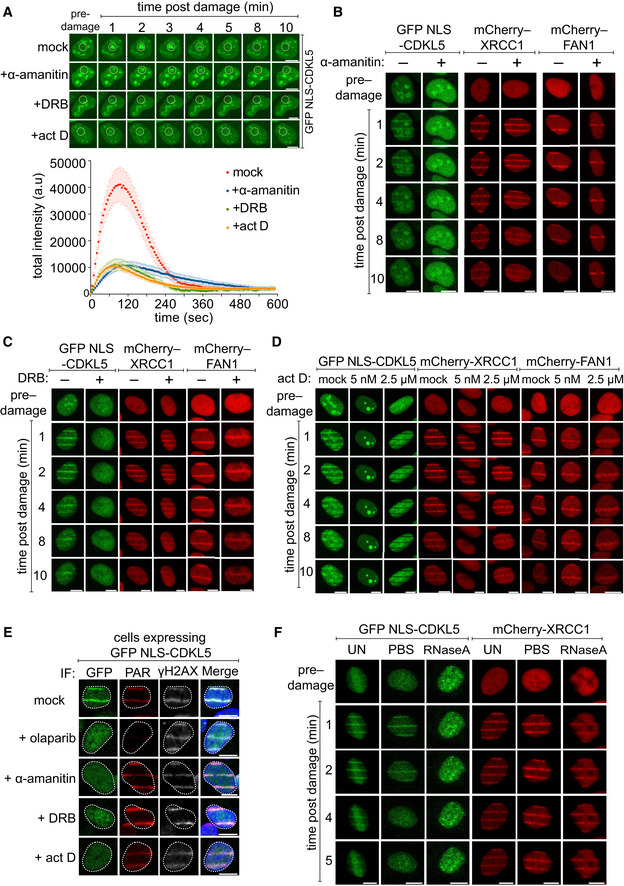
CDKL5 recruitment to DNA lesions requires ongoing transcription A(*Top*) BrdU‐sensitized U‐2‐OS (Flp‐In T‐REx) cells stably expressing GFP‐NLS‐CDKL5 were treated with indicated transcription inhibitors before subjecting to spot micro‐irradiation (405 nm). (*Bottom*) Quantitation of spot intensities. Data represent the mean ± SEM of two independent experiments; > 50 micro‐irradiated cells per point. The “mock” trace shown is identical to the “mock” trace shown in Fig 1D, as the data come from the same set of biological replicates. Scale bar is 10 μm.B–DBrdU‐sensitized U‐2‐OS (Flp‐In T‐Rex) cells stably expressing GFP‐NLS‐CDKL5, mCherry‐XRCC1 or mCherry‐FAN1 were pre‐incubated with indicated transcription inhibitors prior to line micro‐irradiation (355 nm) and time‐lapse imaging. One of three independent experiments is shown. Scale bar is 10 μm.ESame as (B–D) except that BrdU‐sensitized cells stably expressing GFP‐NLS‐CDKL5 were also pre‐incubated with olaparib as control. Cells were subjected to line micro‐irradiation, fixed and then subjected to indirect immunofluorescence using antibodies against GFP, PAR and γH2AX. Scale bar is 10 μm.FStable cell lines were permeabilized and incubated with RNase A or PBS before irradiation and imaging. Scale bar is 10 μm. Source data are available online for this figure.

Although local PAR synthesis is required for CDKL5 recruitment to DNA damage sites, we discovered unexpectedly the recruitment mechanism is more complex. We noticed that the transcription inhibitors actinomycin D or α‐amanitin, which inhibit RNA polymerases (RNAPs) I and II, or DRB (5,6‐dichloro‐1–b‐D‐ribofuranosylbenzimidazole), which blocks RNAP II elongation, abrogated the recruitment of CDKL5 to micro‐irradiation sites (Fig 3A–E); PAR synthesis and recruitment of the PAR‐binding single‐strand break (SSB) repair protein XRCC1 or FAN1 were unaffected under these conditions. Consistent with the idea that RNA synthesis is required for CDKL5 recruitment, incubation of permeabilized cells with RNase A abolished micro‐irradiation tracks formed by CDKL5 but did not affect recruitment of XRCC1 (Fig 3F). Therefore, CDKL5 is recruited to DNA breaks at sites of active transcription.

### A phosphoproteomic screen to identify nuclear targets of CDKL5

**Figure EV2:**
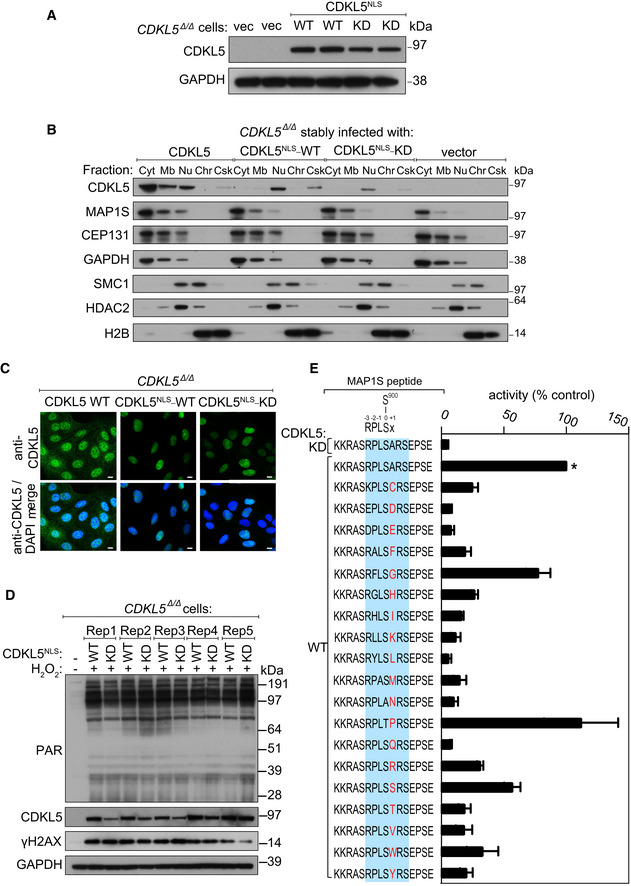
Restricting CDKL5 expression to the cell nucleus Extracts of CDKL5‐disrupted U‐2‐OS (Flp‐In T‐REx) cells (*CDKL5* ^Δ/Δ^) stably expressing CDKL5^NLS^ WT or a K^42^R kinase‐dead mutant (CDKL5^NLS^–KD) or empty vector were subjected to Western blotting with the antibodies indicated. Two different dishes of cells are shown per condition.Subcellular fractionation of lysates from *CDKL5* ^Δ/Δ^ cells stably expressing CDKL5, CDKL5^NLS^ WT or CDKL5^NLS^ KD or empty vector. Lysates were fractionated to isolate proteins found in the following subcellular compartments: cytoplasmic (Cyt), membrane (Mb), nuclear (Nuc), chromatin (Ch) or cytoskeleton (Csk). Fractionated samples were resolved by SDS–PAGE and probed with antibodies shown.
*CDKL5* ^Δ/Δ^ cells stably expressing CDKL5, CDKL5^NLS^ WT or CDKL5^NLS^ KD were subjected to indirect immunofluorescence analysis with anti‐CDKL5 antibodies. Scale bar is 10 μm.
*CDKL5* ^Δ/Δ^ cells stably expressing CDKL5^NLS^ WT or CDKL5^NLS^ KD (or empty vector) were treated with 500 µM H_2_O_2_ for 15 min. Samples were resolved by SDS–PAGE and probed with indicated antibodies or stained with Ponceau S to show equal loading. Rep=biological replicate.Peptide kinase assays to investigate CDKL5 sequence specificity. Anti‐FLAG precipitates from HEK293 cells transiently expressing FLAG‐tagged CDKL5 (wild‐type “WT” or a K^42^R kinase‐dead “KD” mutant) were incubated with synthetic peptides corresponding to sequence around the previously reported CDKL5 phosphorylation site in MAP1S (Ser^900^) designed specifically to investigate the effect of amino acid substitutions A^901^ on the phosphorylation of MAP1S Ser^900^. Assays were done in the presence of [γ‐^32^P]‐labelled ATP‐Mg^2+^, and peptide phosphorylation was measured by Cerenkov counting. Phosphorylation of the control wild‐type MAP1S peptide is taken as 100% (*). The data are represented as mean ± SEM from three independent experiments. The RPXSA motif is shaded in blue, and amino acid substitutions compared with the wild‐type MAP1S Ser^900^ peptide are shown in red. Source data are available online for this figure.

**Figure 4:**
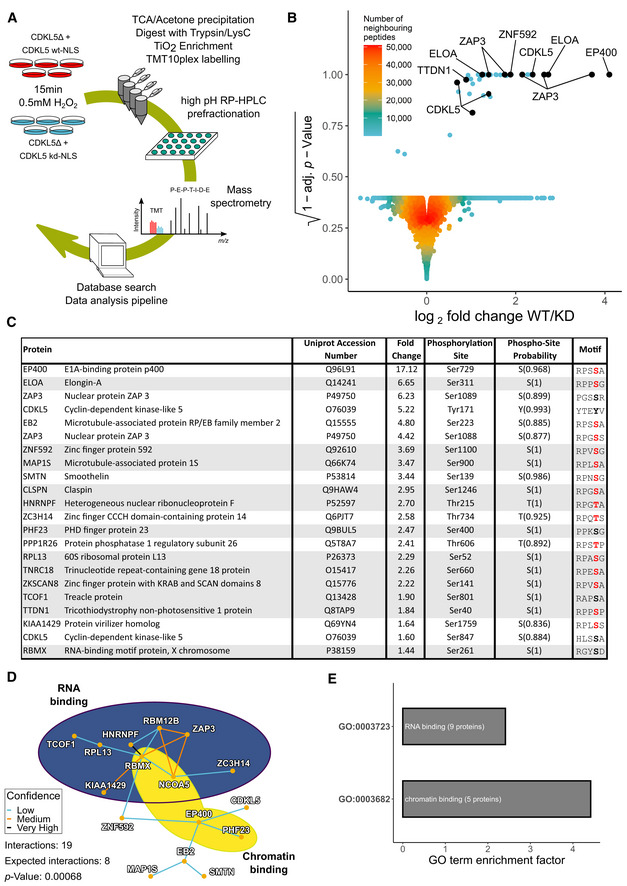
Transcriptional regulators as putative CDKL5 targets Quantitative phosphoproteomic workflow.Volcano plot of the mass spectrometric data from the experiment in (A) (see Table EV1).List of proteins containing phosphorylation sites more abundant in cells expressing CDKL5^NLS^ WT vs KD, with phosphorylation sites having PTM score probabilities > 75% (peptides with a 100% PTM score probability are shaded in grey). Phosphorylation sites with a R–P–X–[S/T]–[A/G/P/S] motif are highlighted in red.Protein–protein interaction network of putative CDKL5 substrates from Table EV1. Confidence levels are based on the STRING database v11.0 combined score with following bins: 150–400: low confidence (blue); 400–700: medium confidence (gold); 700–900: high confidence (not encountered in this dataset); and > 900: very high confidence (black). *P*‐value was calculated as 0.00068.Analysis of GO terms. Significance cut‐off was set as α = 0.01 with at least three proteins identified in the respective group. GO term enrichment factor expresses the relative over‐representation of the GO term within the group of proteins containing a phosphorylation site that is more abundant in WT compared with KD compared with the group of all proteins.

**Figure EV3:**
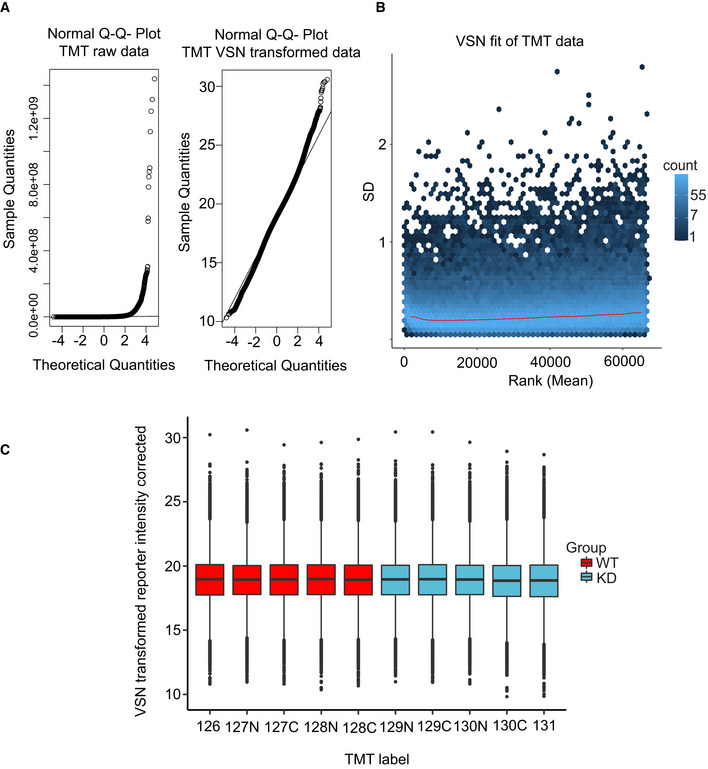
Phosphoproteomic data quality control Left: Normal Q–Q plot of the raw TMT intensity data with large deviations from a normal distribution as seen from datapoints not following the indicated line in the plot. Right: Q–Q plot of the TMT intensity data after VSN transformation. Only minor deviations from the line indicates the transformed data follow a normal distribution to a satisfactory degree. The hypervariable datapoints in the upper quantiles are controlled by the application of the robust implementation of the empirical Bayes algorithm used by *limma* (Phipson *et al*, 2016) and implemented in the analysis scripts.Standard deviation plotted against the intensity rank of the VSN‐transformed TMT data. Red line indicates the mean standard deviation. Line is approximately horizontal, indicating that the variance is not overly dependent on intensity rank and suggests a successful VSN transform.Boxplot of intensity distribution in each TMT channel. No obvious discrepancy between the median values of the individual channels indicates a successful calibration by VSN and no introduction of an obvious intensity bias for any experimental group. The central band of the boxplot indicates the median value, while the hinges represent the first and third quartile (bottom and top of boxplot, respectively). The whiskers extend to the largest/smallest (upper or lower whisker, respectively) datapoint not further than 1.5 times the interquartile range from their respective hinge. The experiment was conducted using five biological replicates of CDKL5^NLS^ WT (WT, red) and CDKL5^NLS^ KD (KD, blue) where each TMT channel represents a single biological replicate from the respective group. Source data are available online for this figure.

The data presented above suggested that nuclear targets of CDKL5 may be involved in transcriptional control, and we speculated that, if so, the nuclear targets of CDKL5 should include transcriptional regulators. Previous screens for CDKL5 targets identified exclusively cytosolic targets. To identify the nuclear targets specifically, we stably expressed CDKL5 wild‐type (WT) or a K^42^R kinase‐dead (KD) mutant (Munoz *et al*, 2018) exclusively in the nucleus of CDKL5‐disrupted U–2–OS cells by adding an artificial nuclear localization signal (NLS). Immunofluorescence and fractionation experiments confirmed that the NLS‐tagged forms of CDKL5 are predominantly nuclear (Fig EV2A–C). We next compared the phosphoproteome of these two cell populations, after exposure to H_2_O_2_ to induce PAR‐dependent CDKL5 retention at DNA breaks (Figs 4A and EV2D; Table EV1). Five biological replicates of each of the two populations (CDKL5^NLS^ WT cells + H_2_O_2_; CDKL5^NLS^ KD cells + H_2_O_2_) were lysed, and Cys residues were reduced and alkylated. After trypsinization of cell extracts, phosphopeptides were enriched by chromatography. The 10 samples were then isotopically labelled with tandem mass tags (TMT), allowing multiplexed and quantitative analysis of all 10 samples, which were combined and analysed together (Rauniyar & Yates, 2014). The pooled sample was fractionated into 60 fractions using basic reverse‐phase liquid chromatography. These fractions were then concatenated into 20 fractions and analysed by LC‐MS/MS. Applying a false discovery threshold (FDR) of 5% identified 46,258 unique peptides of which 36,696 had at least one phosphorylation site with a PTM score probability of ≥ 75%; this yielded 28,185 unique phosphorylation sites. Normalization and intensity distribution in the TMT channels were checked and deemed satisfactory (Fig EV3).

In order to identify putative CDKL5 substrates, mass spectrometric data (Table EV1) were subjected to volcano plot analysis as shown in Fig 4B. This analysis revealed 37 phosphopeptides (31 unique sequences) that were greater in abundance in the CDKL5^NLS^ WT samples compared with CDKL5^NLS^ KD. This group of 37 clustered away from the bulk of phosphopeptides, and all of the phosphopeptides in the cluster had a *P*‐value < 0.0005. Of these 37 phosphopeptides, 22 had a single, unique phosphorylation site (≥ 75% PTM score probability) and were therefore assigned as peptides of interest (Fig 4C). Besides CDKL5 itself, and the previously identified substrates MAP1S and EB2, our analysis revealed a range of nuclear proteins as putative CDKL5 targets. Strikingly, the phospho‐acceptor Ser or Thr residue in almost all of the putative nuclear CDKL5 substrates lies in an extended version of the motif identified for the cytosolic targets: R–P–X–[**S/T**]–[A/G/P/S] (Fig 4C). The R located at the −2 position and the and P located at −1 are known to be essential for Ser/Thr phosphorylation by CDKL5 (Munoz *et al*, 2018). To test whether the residues that can be accommodated at the +1 position *in vitro* agree with the [A/G/P/S] amino acid selection identified above, a range of synthetic peptides based on the sequence around a previously identified substrate MAP1S Ser^900^ were synthesized. Two lysine residues were added at the N‐terminus of each peptide to enable binding to P81 phosphocellulose paper, which enabled isolation of peptides at the end of kinase reactions and quantitation of peptide phosphorylation. The wild‐type peptide sequence was KKRASRPLS^900^ARSEPSE (Fig EV2E), and we tested the impact of substituting A^901^ at the +1 position relative to the S^900^ phospho‐acceptor site for every other amino acid. As shown in Fig EV2E, the MAP1S Ser^900^ peptide was efficiently phosphorylated by FLAG precipitates from extracts of cells expressing C‐terminally FLAG‐tagged CDKL5 but not the FLAG‐CDKL5 K^42^R kinase‐dead mutant. We found that A, G and P were the preferred residues at the +1 position, with the only other amino acid allowing peptide phosphorylation at greater than 50% of wild‐type levels was S (Fig EV2E). These *in vitro* data are in good agreement with the motif R‐P‐X‐[**S/T**]‐[A/G/P/S] that shows remarkably strong enrichment among the putative CDKL5 targets shown in Fig 4C. These data suggest strongly that R‐P‐X‐[**S/T**]‐[A/G/P/S] represents a prerequisite consensus motif for CDKL5 target phosphorylation and suggest hits from our screen with this motif are direct targets of CDKL5.

Gene ontology (GO) analysis showed a striking enrichment of transcription regulators (Fig 4D and E). The top three hits from the screen include EP400, a chromatin‐remodelling transcriptional activator (Pradhan *et al*, 2016); Elongin A (ELOA), a transcriptional elongation factor and component of an E3 ligase complex that ubiquitylates RNAPII (Conaway & Conaway, 2019); ZAP3 (YLPM1), a protein phosphatase 1‐interacting putative nucleoside kinase that binds to hnRNP‐G and transcriptional co‐activators, whose cellular roles are unclear. Other putative CDKL5 substrates include trichothiodystrophy non‐photosensitive 1 (TTDN1), an uncharacterized protein mutated in a form of trichothiodystrophy (TTD), typically caused by failure in transcription‐coupled DNA repair (Heller *et al*, 2015).

**Figure 5:**
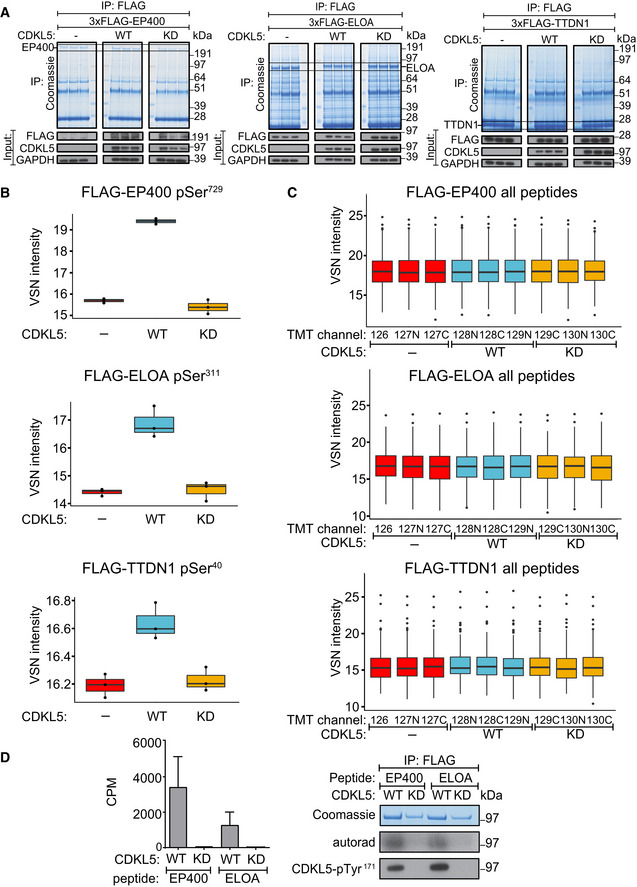
Validating phosphorylation of EP400, ELOA and TTDN1 HEK293 cells were co‐transfected with CDKL5^NLS^ (wild‐type “WT” or kinase‐dead “KD” K^42^R mutant) and either FLAG‐EP400 (*left*), FLAG‐ELOA (*middle*) or FLAG‐TTDN1 (*right*). 24 h later, cells were incubated with H_2_O_2_ (500 µM) for 15 min before being harvested and lysed. Protein extracts were subjected to immunoprecipitation with anti‐FLAG‐agarose beads. Precipitates were subjected to SDS–PAGE and blotting with antibodies shown (bottom panels) or staining with Coomassie Brilliant Blue (top panels). The bands corresponding to the FLAG‐tagged proteins were excised from the gels in A. and processed for mass spectrometric detection of relevant phosphopeptides. Three independent co‐transfection experiments were done for every condition.Boxplots showing VSN‐normalized intensity of phosphopeptides corresponding to EP400‐pSer^729^, ELOA‐pSer^311^ and TTDN1‐pSer^40^ from the experiment in (A). The central band of the boxplot indicates the median value, while the hinges represent the first and third quartile (bottom and top of boxplot, respectively). The whiskers extend to the largest/smallest (upper or lower whisker, respectively) datapoint not further than 1.5 times the interquartile range from their respective hinge. In all cases, the data were derived from 3 biological replicates.Boxplots of the VSN‐adjusted TMT reporter ion intensities for all peptides for each TMT label in the case of FLAG‐EP400, FLAG‐ELOA and FLAG‐TTDN1 from the experiment in (A). The central band of the boxplot indicates the median value, while the hinges represent the first and third quartile (bottom and top of boxplot, respectively). The whiskers extend to the largest/smallest (upper or lower whisker, respectively) datapoint not further than 1.5 times the interquartile range from their respective hinge. Datapoints were further removed, and then, the whiskers are plotted individually. The experiment was conducted using three biological replicates within each respective group, and each TMT channel represents a single biological replicate.Left: Anti‐FLAG precipitates from HEK293 cells transiently expressing FLAG‐tagged CDKL5 (wild‐type “WT” or a K^42^R kinase‐dead “KD” mutant) were incubated with the synthetic peptides indicated, in the presence of [γ‐^32^P]‐labelled ATP‐Mg^2+^, and peptide phosphorylation was measured by the Cerenkov counting. Data are represented as mean ± SEM from three independent experiments. Right: Same but anti‐FLAG precipitates were subjected to SDS–PAGE and autoradiography to detect CDKL5 autophosphorylation, or Western blotting with CDKL5‐pTyr^171^ antibody specific for the CDKL5‐Tyr^171^ autophosphorylation site (Munoz *et al*, 2018). Source data are available online for this figure.

**Figure 6:**
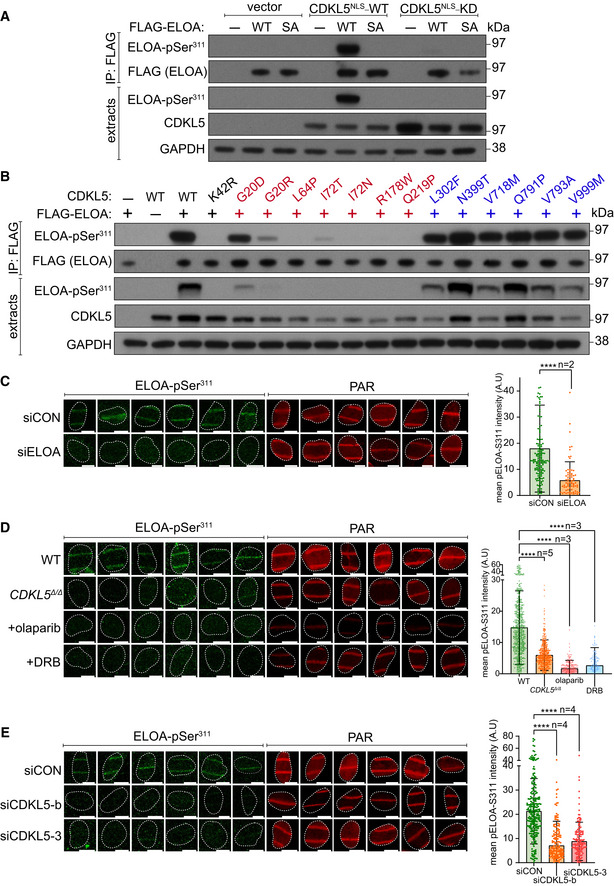
Phosphorylation of ELOA Ser^311^ by CDKL5 on damaged chromatin AHEK293 cells were co‐transfected with CDKL5 (wild‐type “WT” or kinase‐dead “KD” K^42^R mutant) fused to an NLS, and FLAG‐ELOA (wild‐type “WT” or a S^311^A mutant “SA”). Anti‐FLAG precipitates or cell extracts were probed with the antibodies indicated. One of three independent experiments is shown.BSame as (A) showing a range of pathogenic (red) and benign (blue) CDKL5 variants.C–EWild‐type (WT), *CDKL5*‐disrupted (*CDKL5^Δ^* ^/^ *^Δ^)* or siRNA‐transfected cells were subjected to indirect immunofluorescence analysis with the indicated antibodies at laser tracks. Quantification of ELOA‐pSer^311^ signal at the laser tracks is shown. Data represent mean ± SD of total pELOA Ser^311^ intensities in different biological replicates as indicated (*n*). For simplicity, only intensities greater than zero are shown. Statistical significance was assessed by one‐way ANOVA test or the unpaired t‐test with Welch's correction. Asterisks **** indicate *P*‐values of < 0.0001. Scale bar is 10 μm. Source data are available online for this figure.

We sought to validate EP400, ELOA and TTDN1 as CDKL5 substrates by testing the phosphorylation of these proteins expressed in HEK293 cells. Extracted ion chromatogram (XIC) analysis of tryptic phosphopeptides isolated from FLAG‐tagged EP400 (pSer^729^; UniProtKB accession Q96L91), ELOA (pSer^311^; UniProtKB accession Q14241) and TTDN1 (pSer^40^; UniProtKB accession Q8TAP9) confirmed phosphorylation of these proteins in cells when co‐expressed with wild‐type, but not kinase‐dead, CDKL5 (Fig 5A–C). Furthermore, CDKL5 robustly phosphorylated synthetic peptides corresponding to EP400 Ser^729^ and ELOA Ser^311^ demonstrating direct phosphorylation (Fig 5D). We chose ELOA for further investigation, because it has already been implicated in the DDR (Weems *et al*, 2015) and generated antibodies specific for phospho‐Ser^311^ to further characterize ELOA phosphorylation. Co‐expression with WT, but not KD CDKL5, markedly increased Ser^311^ phosphorylation of FLAG‐ELOA, but not of an ELOA Ser^311^Ala mutant (Fig 6A). We also assessed the impact of CDD‐associated CDKL5 mutations, which are located predominantly in the kinase catalytic domain (Krishnaraj *et al*, 2017). As shown in Fig 6B, a series of CDD‐associated mutations severely reduced CDKL5 activity towards ELOA‐pSer^311^, whereas a series of benign variants did not (Fig 6B). Therefore, ELOA phosphorylation is a potential biomarker of CDKL5 activity that may be useful in the clinic.

### ELOA recruitment and CDKL5‐dependent phosphorylation of ELOA Ser^311^ at DNA damage sites require PAR synthesis and active transcription

**Figure EV4:**
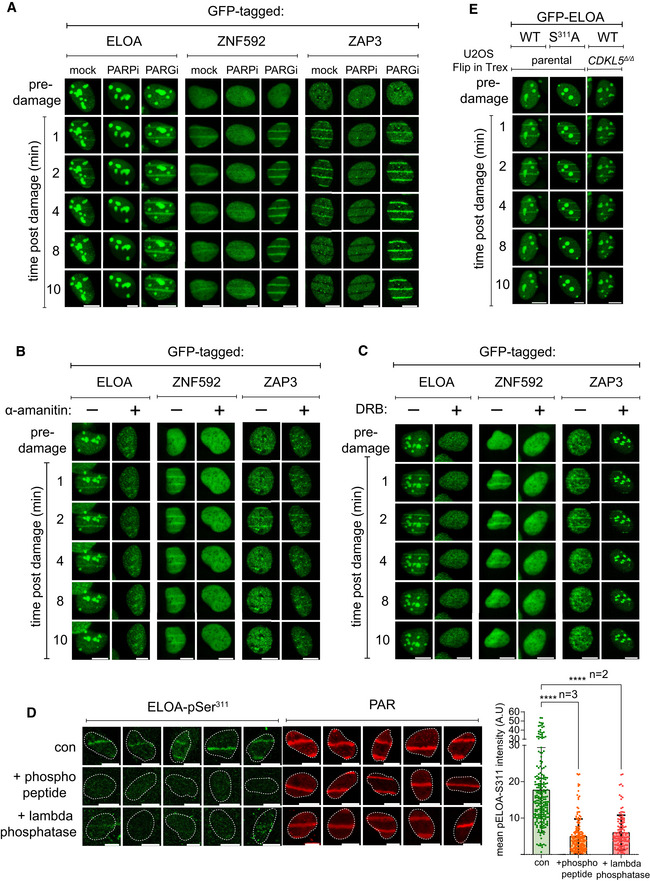
Recruitment of CDKL5 substrates to DNA damage sites A–CBrdU‐sensitized U‐2‐OS (Flp‐In T‐REx) cells stably expressing GFP‐tagged forms of the proteins indicated were pre‐incubated with (A) olaparib (PARPi; 5 µM) or PD00017273 (PARGi; 0.3 µM, 1 h), (B) α‐amanitin (20 µg/ml, 8 h) or (C) DRB (100 µM, 2 h) prior to line micro‐irradiation (355 nm) and time‐lapse imaging. One of three independent experiments is shown. Scale bar is 10 μm.DBrdU‐sensitized U‐2‐OS (Flp‐In T‐REx) cells were subjected to nuclear line micro‐irradiation (355 nm). Cells were fixed and then mock‐treated (con) or treated with lambda‐phosphatase prior to incubation with the primary antibodies indicated. Alternatively, ELOA‐pSer^311^ phosphopeptide was included during incubation with the primary antibodies before indirect immunofluorescence analysis. Quantification of ELOA‐pSer^311^ signal at the laser tracks is shown. Data represent mean ± SD of total pELOA Ser^311^ intensities in different biological replicates as indicated (*n*). For simplicity, only intensities greater than zero are shown. Statistical significance was assessed by one‐way ANOVA test. Asterisks **** indicate *P*‐values of < 0.0001. Scale bar is 10 μm.EBrdU‐sensitized U‐2‐OS cells (Flp‐In T‐Rex; *CDKL5*–disrupted (*CDKL5^Δ^* ^/^ *^Δ^)* or parental cells) stably expressing GFP‐tagged ELOA wild‐type (WT) or S^311^A mutant were line‐micro‐irradiated and imaged after at the time points indicated. Source data are available online for this figure.

We speculated that ELOA might be phosphorylated by CDKL5 at DNA damage sites, and in this light, we wondered whether ELOA is recruited to DNA damage sites perhaps by a similar mechanism to CDKL5. In agreement with this idea, ELOA recruitment to laser micro‐irradiation tracks was rapid, transient and inhibited by olaparib, α‐amanitin and DRB. Similar results were obtained for other putative CDKL5 substrates such as ZNF592 and ZAP3 (Fig EV4A–C), but not EP400 (data not shown). Strikingly, we observed CDKL5‐dependent phosphorylation of endogenous ELOA Ser^311^ at micro‐irradiation tracks. Signal intensity was reduced by depletion of ELOA (Fig 6C) or by incubation of cells with lambda‐phosphatase or the ELOA Ser^311^ phosphopeptide antigen (Fig EV4D), thereby confirming antibody specificity. ELOA phosphorylation was reduced by disruption or depletion of CDKL5 (Fig 6D and E), or by olaparib or DRB, which block recruitment of both CDKL5 and ELOA (Fig 6D). These data reveal CDKL5‐dependent phosphorylation of ELOA at DNA damage sites, involving a common mechanism of recruitment for both kinase and substrate. Notably, the recruitment of ELOA to micro‐irradiation tracks was not dependent on the phosphorylation of Ser^311^ as recruitment appeared normal in CDKL5 knockout cells, and recruitment of an ELOA S^311^A mutant was indistinguishable from wild type (Fig EV4E).

### CDKL5 facilitates silencing of transcription near DNA breaks

**Figure 7:**
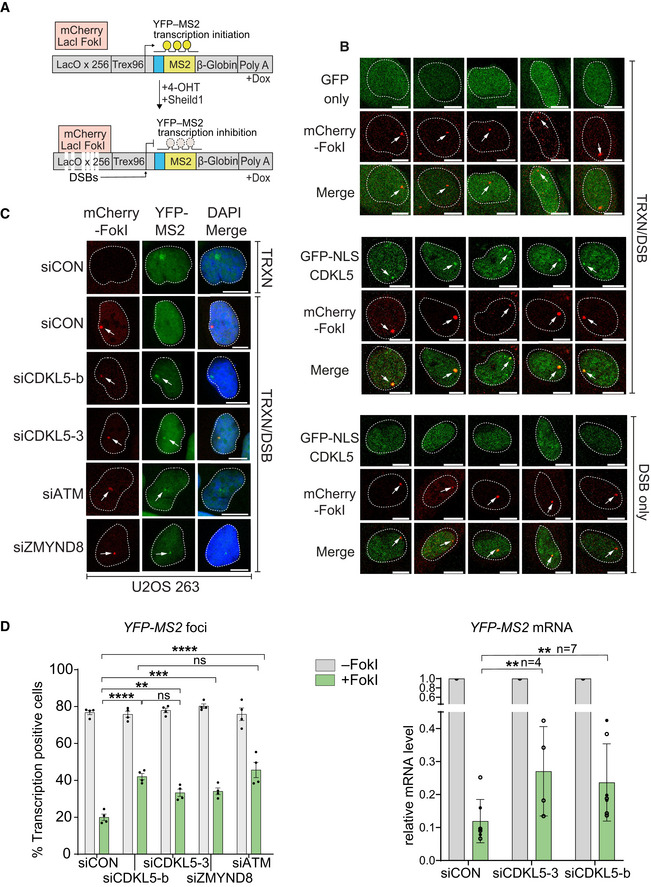
CDKL5 facilitates transcriptional repression at DNA breaks Cartoon of reporter construct (Tang *et al*, 2013) in which induction of the mCherry‐tagged FokI endonuclease (with 4‐OHT) results in double‐strand break (DSB) in a region upstream of a doxycycline‐inducible reporter gene (YFP‐MS2). Ongoing transcription of the reporter gene can be visualized by the presence of a YFP‐MS2 fusion protein that binds stem–loop structures in the nascent transcript.CDKL5 is recruited to FokI‐induced DSBs. GFP alone (top panel) or GFP‐NLS‐CDKL5 (middle and bottom panels) was stably expressed in U‐2‐OS 265 DSB reporter cells. Cells were mock‐treated or treated with 1 µg/ml doxycycline for 3 h to induce transcription of the reporter gene. An hour before DSB induction, cells were treated with 0.3 µM PARG and 10 µM ATM inhibitor. Site‐specific DSBs were induced by treating the cells with 4‐OHT and Sheild1 ligand. Cells were live‐imaged at 37°C, between 15 and 25 min following DSB induction. Representative image showing the recruitment of GFP‐NLS‐CDKL5 to FokI‐induced DSBs upstream of transcriptionally active (middle) but not the inactive (bottom) MS2 gene. GFP alone is used a control (top). White arrowheads mark the location of the mCherry‐FokI upstream of the MS2 reporter cassette. Images are representative of multiple technical replicates of three independent experiments. Scale bar is 10 μm.Representative image for U–2–OS 263 IFII cells harbouring the reporter construct and transfected with the siRNAs indicated. After addition of doxycycline, transcription was monitored in cells ± induction of FokI by quantification of YFP(–MS2) foci. Arrows indicate sites of FokI‐mediated DSB (mCherry) and YFP‐MS2 transcript. “TRXN”: doxycycline added; “TRXN/DSB”: doxycycline added with 4‐OHT. Scale bar is 10 μm.(Left) Quantification of transcription in U‐2‐OS 263 IFII reporter cells from experiment in B. > 150 cells were analysed per condition per experiment. The mean ± SD from four independent experiments is shown. (Right) Quantitative RT–PCR analysis of YFP‐MS2 mRNA in U‐2‐OS 263 reporter cells. Data represent mean ± SD in different biological replicates as indicated (*n*). Statistical significance for all the data was assessed by two‐way ANOVA test, **P < 0.01, ***P < 0.001 and ****P < 0.0001; ns—not significant. Source data are available online for this figure.

**Figure EV5:**
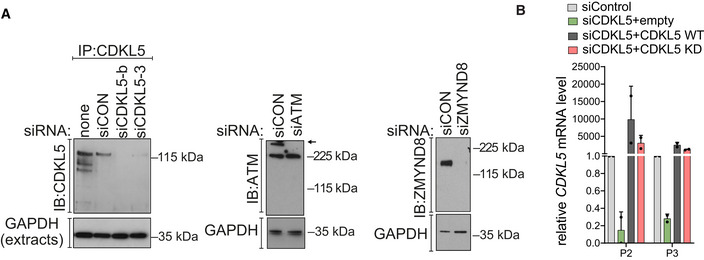
siRNA‐mediated knockdown of proteins in U‐2‐OS reporter cells CDKL5, ATM and ZMYND8 were depleted in U‐2‐OS 263 IFII reporter cells using indicated siRNA. siCON—non‐targeting control.qRT–PCR measurements in U2OS I‐PpoI cells to validate the silencing efficiency of siCDKL5 and the subsequent rescue efficiency of ectopic expression of siRNA‐resistant forms of CDKL5 wild‐type (WT) and K^42^R kinase‐dead mutant (KD). P2 and P3 primer pairs were used to analyse the changes in mRNA levels. The mean ± SD from two qPCR replicates of two independent experiments is shown. Source data are available online for this figure.

The ontological enrichment for transcription regulators among the nuclear CDKL5 substrates suggested a role in transcriptional control at DNA damage sites. Breaks in genomic DNA are known to silence adjacent genes (Shanbhag *et al*, 2010; Pankotai *et al*, 2012; Gong *et al*, 2015), and we therefore tested a role for CDKL5, using two different experimental systems. First, we used a reporter system in which addition of 4‐hydroxytamoxifen (4‐OHT) induced nuclear translocation of mCherry‐FokI nuclease, which induces a cluster of double‐strand breaks (DSBs) upstream of a doxycycline‐inducible reporter gene; DSB induction silences the reporter cassette (Fig 7A) (Shanbhag *et al*, 2010). We set out to test whether CDKL5 is recruited to FokI‐induced DSB, mindful that it is difficult to capture transiently recruited PAR‐dependent proteins at DSB foci. To capture CDKL5 recruitment, reporter cells were pre‐incubated with PARG inhibitor, which causes a modest extension in CDKL5 retention time, and the ATM inhibitor KU455933 to prevent transcriptional silencing; 4‐OHT was added for just 15 min to induce FokI. Under these conditions, between 5 and 20% of cells showed clear mCherry‐FokI foci, and around 3–5% cells showed co‐localization of GFP‐tagged CDKL5 with mCherry‐FokI foci (Fig 7B). Despite the low proportion of cells displaying GFP‐CDKL5 foci, three biological replicates of this experiment, with multiple technical replicates done on the same day per biological replicate, yielded similar data. GFP alone never co‐localized with mCherry‐FokI. In each experiment, we included a control where doxycycline was omitted so that transcription of the reporter cassette was off. Under these conditions, no GFP‐CDKL5 foci were observed co‐localizing with mCherry‐FokI (Fig 7B). These data suggest (but do not prove) that CDKL5 is recruited specifically to DSB where there is active transcription. We noticed that siRNA‐mediated depletion of CDKL5 weakens silencing of the reporter cassette, similar to depletion of ATM or ZMYND8, which was previously reported to be involved in mediating transcriptional silencing near DNA breaks (Figs 7C and D, and EV5A) (Shanbhag *et al*, 2010; Gong *et al*, 2015).

**Figure 8:**
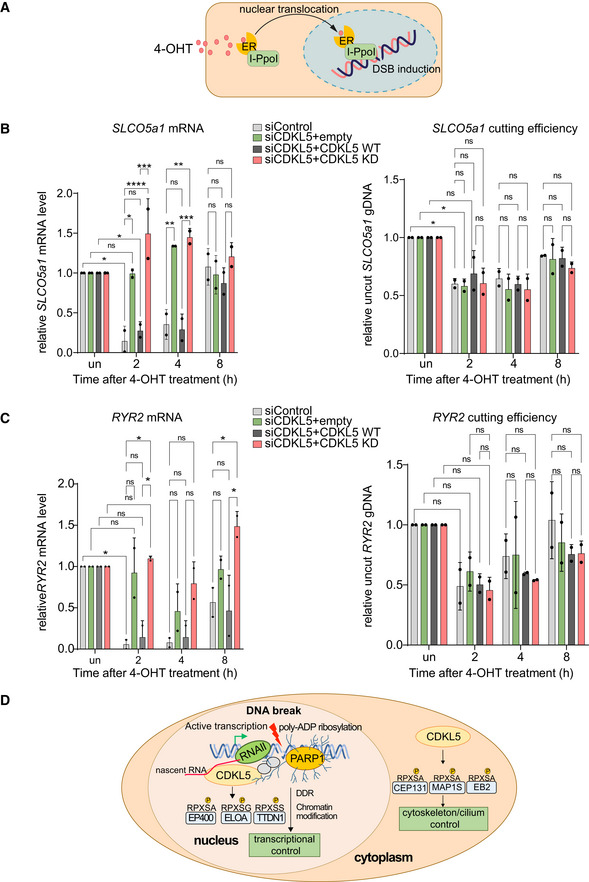
Kinase activity of CDKL5 facilitates transcriptional silencing ASchematic diagram of the I‐PpoI system for inducing DNA breaks in the nuclear human genome. Addition of 4‐OHT to U‐2‐OS‐pEP15 cells stably expressing the I‐PpoI endonuclease fused to the estrogen receptor (ER) induces nuclear translocation of the fusion protein and cleavage cleavage of FokI recognition sites in nuclear DNA resulting, leading to DSB induction.B, CQuantitative PCR with reverse transcription (qRT–PCR) analysis of *SLCO5a1* (B) and *RYR2* expression levels (C) (left panels) U‐2‐OS HA‐ER‐I‐PpoI cells depleted of CDKL5 transiently transfected with FLAG‐tagged CDKL5 wild‐type (WT) or a K^42^R‐mutated kinase‐dead (KD) mutant, or empty vector, at the times indicated after inducing I‐PpoI. The mean ± SD from two qPCR replicates of two independent experiments is shown. Statistical significance for all the data was assessed by two‐way ANOVA test *P < 0.05, **P < 0.01, ***P < 0.001 and ****P < 0.0001; ns—not significant. I‐PpoI‐mediated cutting efficiency in the relevant gene is shown in the right‐hand panel (see Materials and Methods).DSchematic diagram depicting CDKL5 functions in nucleus and cytosol. Source data are available online for this figure.

Next, we took advantage of a system where inducible overexpression of the site‐specific meganuclease I‐PpoI cuts 14–30 times in the human genome, including active genes such as *SLCO5a1* and *RYR2* (Fig 8A) (Pankotai *et al*, 2012; Caron *et al*, 2019 *)*. As shown in Fig 8B (left panels), a strong decrease in *SLCO5a1* mRNA levels was observed 2 h after I‐PpoI induction, when DSB induction was maximal (right panels), but mRNA levels returned to basal levels by 8 h consistent with previous reports (Pankotai *et al*, 2012; Caron *et al*, 2019). Strikingly, CDKL5 depletion largely abolished the I‐PpoI‐induced silencing of *SLCO5a1* (Fig 8B, left panels) but did not affect either formation or repair of the I‐PpoI‐mediated DSB at these loci (Fig 8B, right panels). Ectopic expression of an siRNA‐resistant form of CDKL5 rescued the defect in I‐PpoI‐mediated silencing of *SLCO5a1* caused by the CDKL5 siRNA, whereas a K^42^R‐mutated kinase‐dead form of CDKL5 did not (Figs 8C and EV5B). Similar data were obtained for the *RYR2* gene known to be silenced by I‐PpoI induction (Fig 8C). Therefore, the kinase activity of CDKL5 facilitates the transcriptional silencing of genes at or close to DSB.

## Discussion

In this study, we show that CDKL5 is recruited to DNA damage induced by laser micro‐irradiation and DNA breaks, and the available data indicate that active transcription is required—transcriptional inhibitors block CDKL5 recruitment to sites of laser micro‐irradiation; pre‐incubation of cells with RNase A abolishes CDKL5 signal at laser tracks; CDKL5 does not appear to be recruited to FokI nuclease‐induced DSB without transcription nearby. One interpretation of these data is that CDKL5 acts as a coincidence detector recognizing breaks (and maybe other DNA lesions) that occur at sites of active transcription (Fig 8D). CDKL5 recruitment to micro‐irradiation sites is PAR‐dependent, and CDKL5 can bind PAR directly; therefore, the direct binding of CDKL5 to PARP1‐generated PAR may allow detection of DNA breaks. However, the mechanism whereby CDKL5 detects transcriptional activity remains to be determined. It was reported that the binding of the central BRCT domain of XRCC1 to both PAR and DNA is required for recruitment to DNA breaks (Polo *et al*, 2019). By analogy, it is tempting to speculate that binding of the CDKL5 recruitment region to both nascent RNA and PAR is required for retention at DNA breaks, but more work is needed to test this idea.

Consistent with a requirement of transcriptional activity for CDKL5 recruitment to DNA breaks, quantitative phosphoproteomic screening for nuclear substrates revealed that CDKL5 phosphorylates a range of transcriptional regulators including EP400, TTDN1, ZAP3 and ELOA. In almost all of the hits from our screen, the phospho‐acceptor Ser/Th residues lies in the motif R‐P‐X‐[S/T]‐[A/G/P/S], a motif that our *in vitro* experiments pointed to independently as a CDKL5 consensus motif. Therefore, it is likely that the hits from our screen are direct targets of CDKL5, and we found that CDKL5 can directly phosphorylate the relevant site in EP400 and ELOA. The motif R‐P‐X‐[S/T]‐[A/G/P/S] identified in the current study is likely to represent the definitive CDKL5 consensus motif, and we predict that proteins lacking such a motif cannot be a direct substrate of this kinase.

We detected CDKL5‐dependent phosphorylation of ELOA Ser^311^ at sites of DNA damage that was prevented by inhibitors of PARP or transcription, which block recruitment of both CDKL5 and ELOA. These data suggest a model where a common recruitment mechanism involving PARylation and nascent RNA synthesis juxtaposes both kinase and substrate at DNA damage sites, enabling robust phosphorylation of substrate by kinase to precipitate a local transcriptional response (Fig 8D). It is not yet clear whether recruitment to DSB also affects the intrinsic activity of CDKL5 nor is it known whether PAR binding affects CDKL5 catalytic function. These possibilities will be interesting to investigate.

The apparent requirement for local transcription in CDKL5 recruitment to DNA breaks, and the enrichment of transcriptional regulators in the CDKL5 substrate screen, pointed strongly to a role in modulating transcription at DNA breaks. A well‐documented response to DNA lesions such as double‐strand breaks is the silencing of transcription near the lesion, presumably to facilitate access to repair proteins and proteins that reset chromatin status before and after repair (Shanbhag *et al*, 2010; Marnef *et al*, 2017; Tufegdzic Vidakovic *et al*, 2020). In two different experimental systems, CDKL5 was found to facilitate the silencing of genes harbouring DNA breaks (Figs 7 and 8). In particular, the silencing of active endogenous genes induced by I‐PpoI‐induced DSB was affected profoundly by inhibiting CDKL5 kinase activity (Fig 8B and C). However, the underlying mechanisms are not yet clear, and it will be important to study which CDKL5 substrates are most relevant to transcriptional repression at DSB. In a broader context, it will be important to investigate how phosphorylation affects CDKL5 substrates such as ELOA, EP400 and TTDN1. As an E3 ligase, ELOA is likely to ubiquitylate a range of proteins at DNA damage sites, and it is possible this activity, and/or the transcriptional elongation‐promoting activity of ELOA, is modulated by CDKL5. EP400 is a chromatin remodeller important for transcriptional activation, which could be modulated by CDKL5 (Pradhan *et al*, 2016). The cellular roles of TTDN1 are unclear, but mutations in the *TTDN1* gene cause a form of trichothiodystrophy (TTD) referred to as non‐photosensitive (NPS) TTD (Nakabayashi *et al*, 2005; Heller *et al*, 2015) typified by seizures and symptoms seen in CDKL5‐associated diseases (Heller *et al*, 2015). Thus, functional connections between TTDN1, CDKL5 and transcriptional control will be interesting to pursue.

We are interested in the possibility that CDKL5 controls transcriptional elongation even in the absence of genotoxic insult. The induction of transient programmed DSBs by topoisomerases I and II in gene promoters has been linked to the control of transcriptional activity particularly in neurons (Ju *et al*, 2006; King *et al*, 2013; Madabhushi *et al*, 2015). Also, DNA damage‐responsive PARPs have been implicated in transcriptional elongation in cells that had not been exposed to genotoxic insults (Gibson *et al*, 2016). Tying these observations together, programmed DNA breakage in active genes may create a PAR signal that recruits CDKL5 to achieve local phosphorylation of the substrates identified in this study, leading to the modulation of transcription. A function of this kind could have a major impact on brain function, which would help to explain the symptoms of disease caused by mutations in *CDKL5* (McKinnon, 2016).

At present, CDD is treated with anti‐epileptic drugs, but these drugs treat the symptoms not the cause of CDD, and most infants with CDD become refractory within months of starting treatment (Kadam *et al*, 2019). Thus, a better rationally designed treatment for CDD and other CDKL5‐related conditions is needed. Most CDKL5 mutations in CDD severely reduce activity towards substrates, but it is likely CDD is caused by failure to phosphorylate the appropriate substrates. One possible avenue for the future would be to use cell‐based assays employing phospho‐specific antibodies against CDKL5 targets, to screen for small molecules or gene deletions that rescue phosphorylation of these targets. Such drugs might work by upregulating compensating kinases, other CDKL kinases perhaps, or inhibiting negative regulators of CDKL5 substrate phosphorylation. Better tools are needed for screens of this kind, such as rabbit monoclonal phospho‐specific antibodies against ELOA or EP400, which might also be useful biomarkers in gene therapy efforts that are in the pipeline.

## Materials and Methods

### Reagents

All reagents including antibodies, cDNA clones, oligonucleotides and peptides used in the present study are enlisted in Table EV2. All cDNA clones and antibodies generated in‐house, and datasheets for each plasmid, can be requested via the MRC‐PPU Reagents and Services reagents website at the following link: https://mrcppureagents.dundee.ac.uk/reagents‐from‐paper/rouse‐CDKL5‐paper‐3.

### ELOA phospho‐Ser^311^ antibodies

ELOA‐pSer^311^ antibodies were raised by MRC‐PPU Reagents and Services at the University of Dundee in sheep and purified against the relevant antigen: (DA081; 3^rd^ bleed; raised against the peptide KEENRRPPS*GDNARE conjugated to bovine serum albumin). Sheep were immunized with the peptide antigen followed by four further injections 28 days apart, with bleeds taken seven days after each injection.

### Cell lines and cell culture

All cells were kept at 37°C under humidified conditions with 5% CO_2_. HEK293, HEK293FT and U‐2‐OS Flp‐In T‐REx, U‐2‐OS 263 IFII reporter cells and U‐2‐OS 265 reporter cells were grown in Gibco DMEM (Life Technologies, Paisley, UK) supplemented with 100 U/ml penicillin, 100 µg/ml streptomycin, 1% (v/v) l‐glutamate (GIBCO, Invitrogen), 1% (v/v) sodium pyruvate and 1% (v/v) non‐essential amino acids, 10% (v/v) foetal bovine serum or 10% (v/v) TET System‐approved FCS for U–2–OS reporter cell lines (631106; Takara Bio). U‐2‐OS‐pEP15 cells (Caron *et al*, 2019) were maintained in 1 mg/ml glucose phenol red‐free DMEM (Lonza) supplemented with steroid‐free FBS (Thermo Fisher Scientific), 1% antibiotic–antimycotic (Sigma‐Aldrich), GlutaMAX™‐I (Gibco) and 800 µg/ml G‐418 (Sigma‐Aldrich). U‐2‐OS (Flp‐In T‐REx) cells were maintained in 10 µg/ml blasticidin. Hygromycin (100 µg/ml) or puromycin (2 µg/ml) was used to select for the integration of constructs in Flp‐In recombination sites. All cell lines were regularly tested for mycoplasma contamination. U‐2‐OS Flp‐In T‐REx *CDKL5* ^Δ/Δ^ cells were described previously (Munoz *et al*, 2018).

### Cell transfections

HEK293 cells were transfected using the calcium phosphate transfection protocol as previously described (Munoz *et al*, 2018). Cells were seeded at a confluence of 20–30% in 15‐cm plates and 24 h later were co‐transfected with a total of 10 µg of plasmid (5 µg + 5 µg in the case of plasmid co‐transfection). Cells were incubated with the transfection mixture for 24 h before being harvested and lysed.

For transient expression of GFP‐tagged proteins in U‐2‐OS cells, cells were transfected with 1–2 µg of pcDNA5 FRT/TO plasmids using GeneJuice Transfection Reagent (Millipore) onto 1 × 10^5^ adhered U‐2‐OS or U‐2‐OS Flp‐In T‐Rex cells in 2 ml media in a 3.5‐cm glass‐bottom dish (FD35‐100, WPI). 8 h following transfection, cells were incubated overnight with 0.5–1 µg/ml tetracycline hydrochloride to induce expression of the target protein.

For siRNA‐mediated knockdown of proteins, cells were transfected with a 100 nM suspension of relevant siRNA duplexes (Eurofins or Dharmacon) or siRNA SMARTpools (Dharmacon) using Lipofectamine RNAi‐MAX transfection reagent (13778150, Invitrogen, Paisley, UK) as per manufacturer's guidelines. Cells were analysed for 60–72 h following transfection. siRNA sources and sequences are outlined in Table EV2.

### Generation of stable cell lines using the Flp‐In T‐REx system

To generate U–2–OS (Flp‐In T‐REx) cells stably expressing target proteins, cells were co‐transfected with 9 µg of POG44 Flp‐recombinase expression vector (Thermo Fisher) and 1 µg of pcDNA5 FRT/TO‐target protein, using GeneJuice Transfection Reagent (Millipore). 48 h following transfection, cells were selected in the presence of 100 µg/ml hygromycin and 10 µg/ml blasticidin in the medium. Around 10–12 days later, surviving colonies were pooled together and resulting cultures were analysed for the expression of target protein following induction with increasing amounts of tetracycline hydrochloride (T3383; Sigma‐Aldrich).

### Retrovirus production and target cell infection for the constitutive expression of target proteins

To generate cells stably expressing nucleus‐restricted CDKL5 (NM_003159.2 with silent changes t996c and t2118c), *CDKL5^Δ^* ^/^ *^Δ^* cells (Munoz *et al*, 2018) were infected with retroviruses expressing wild‐type CDKL5 with an exogenous nuclear localization signal (CDKL5^NLS^ WT), a kinase‐dead (K^42^R) CDKL5^NLS^ KD or an empty vector. Similarly, GFP‐NLS‐CDKL5 or GFP alone was stably expressed in U‐2‐OS 265 reporter cells by retroviral transduction. Briefly, HEK293FT cells were transfected with respective expression plasmid constructs along with the GAG/Pol and VSVG constructs required for retroviral production, using calcium phosphate transfection protocol as previously described (Munoz *et al*, 2018). 48 h following transfections, retrovirus‐containing medium from the cell dish was collected, filtered and applied along with polybrene (8 µg/ml) to the target cells for 24 h. Medium was replaced with fresh medium containing appropriate selection antibiotic for another 36 h. Surviving cells were pooled together, and successful retrovirus integration is confirmed though Western blotting. A list of plasmid constructs is included in Table EV2.

### Whole‐cell extract preparation and Western blotting

Cell pellets were lysed on ice for 30 min in ice‐cold lysis buffer (50 mM Tris–HCl (pH 7.4) buffer containing 0.27 M sucrose, 150 mM NaCl, 1% (v/v) Triton X‐100, 0.5% (v/v) Nonidet NP‐40 and 0.1% (v/v) 2‐mercaptoethanol) supplemented with a protease inhibitor cocktail (cOmplete™, EDTA‐free Protease Inhibitor Cocktail), benzonase (Novagen, 50 U/ml) and microcystin‐LR (catalogue number: 33893, Sigma) at a final concentration of 10 ng/ml and phosphatase inhibitor cocktail‐2 (P5726; Merck) at 1% (v/v). The lysate was cleared by centrifugation at 17,000 *g* for 15 min, and the supernatant was collected for protein measurement by the Bradford assay and stored at −80°C. For Western blotting, the whole‐cell extract (40 µg) was mixed with LDS–PAGE sample buffer (Thermo Fisher) containing 5% (v/v) 2‐mercaptoethanol before boiling at 95°C. Samples were resolved by 4–12% Bis–Tris SDS–PAGE gradient gels (NuPAGE, Thermo Fisher) followed by transfer onto a Hybond C Extra Nitrocellulose Membrane (GE1060000; GE Healthcare) for 105 min at 100 V. The membrane was blocked in 5% (w/v) non‐fat dry milk in TBS–Tween‐20 (0.2% v/v) for 1 h and probed with diluted primary antibodies. The membrane was washed three times in TBS–Tween‐20 (0.1%(v/v)), incubated with secondary antibodies diluted in blocking buffer for 1 h, and washed three times in TBS–Tween‐20 (0.1% (v/v)) prior to developing the membrane using SuperSignalTM West Pico PLUS Chemiluminescent Substrate (Thermo) and capturing the signal on an X‐ray film. See Table EV2 for antibody and dilution information.

### Subcellular fractionation

Subcellular fractionation experiments were performed using the Thermo Fisher Subcellular Protein Fractionation Kit for Cultured Cells (catalogue number: 78840). Briefly, 15‐cm plates of cells were washed with PBS and cells harvested using trypsin–EDTA solution. Cell pellets were washed twice with ice‐cold PBS and then resuspended in 500 µl of CEB buffer. Fractionation was subsequently done following the manufacturer's instructions and using the following amounts of each one of the buffers: 500 µl of MEB buffer and 250 µl for all the other buffers. Protein concentration in each fraction was measured, and samples were resuspended in LDS sample buffer and boiled before being subjected to SDS–PAGE.

### *In vitro* peptide phosphorylation reactions

HEK293 cells were transiently transfected with either wild‐type or kinase‐dead (K42R) CDKL5‐FLAG‐expressing constructs, and 48 h later, plates were washed in cold PBS and lysed in ice‐cold buffer (50 mM HEPES (pH 7.5), 1% (v/v) Triton X‐100, 0.27 M sucrose and 300 mM NaCl) freshly supplemented with protease inhibitor cocktail (cOmplete™, EDTA‐free), 10 mM iodoacetamide, 10 ng/ml microcystin‐LR, 2% (v/v) phosphatase inhibitor cocktail‐2 (Sigma‐Aldrich) and 500 U/ml universal nuclease. Lysates were then cleared by centrifugation for 10 min at 20,000 *g* at 4°C, and protein concentration was measured. Extracts (˜ 2.0 mg) were then incubated with 10 µl (settled) anti‐FLAG agarose M2 affinity beads (Sigma‐Aldrich) for 2 h at 4°C. Beads were washed five times in lysis buffer containing 1 M NaCl and then twice in kinase buffer (50 mM Tris 7.5, 10 mM MgCl_2_, and 0.1 mM EGTA). Beads were resuspended in 15 µl kinase buffer containing 0.15 mM peptide substrate and 0.1% (v/v) 2‐mercaptoethanol. Reactions were initiated with the addition of 5 µl [γ‐^32^P]‐ATP (0.1 mM), incubated for 30 min at 30°C with constant shaking and stopped by adding 10 µl of 0.5 M EDTA. Samples were centrifuged at 20,000 *g*, and supernatants (30 µl) were spotted onto P81‐phosphocellulose paper. Papers were then washed 5 times in 75 mM orthophosphoric acid, once in acetone, and dried. ^32^P incorporation in each sample was measured by the Cerenkov counting using a PerkinElmer TriCarb scintillation counter. Beads were resuspended in LDS sample buffer, boiled and subjected to SDS–PAGE followed by Coomassie staining.

### Laser micro‐irradiation

#### “Line” micro‐irradiation

Around 1 × 10^5^ cells expressing fluorescently tagged proteins of interest were seeded in 3.5‐cm glass‐bottom dishes (FD35‐100 for 24 h in media containing 10 µM bromodeoxyuridine [BrdU–Sigma] and 0.5–1 µg/ml tetracycline hydrochloride [Sigma]). Shortly prior to irradiation, cells were washed with PBS and the medium was replaced with warm, low absorption medium (31053; Thermo). Cells were placed in an incubator chamber at 37°C with 5% CO_2_ supplementation mounted on a Leica TCS SP8X microscope system (Leica Microsystems). Laser micro‐irradiation was performed using a protocol adapted from Mistrik *et al* (2016). Briefly, a striation pattern was generated by scanning bidirectionally at either 16 × 16 or 32 × 32 pixel resolution using a 355‐nm laser (coherent), resulting in a pattern of 16 or 32 horizontal lines across the imaging field. The laser dose was adjusted by altering the laser scanning speed and the number of scanning iterations per line. Typically, irradiation was performed by scanning at 5 Hz with three iterations per line. The power at the objective (approximately 1.5 mW) was measured using a power meter (Thorlabs). Using the above settings, we typically irradiated at approximately 1.4–2.8 J/m^2^. Laser micro‐irradiation experiments were performed using a Leica HC PL APO CS2 63×/1.20 water objective, using a predefined imaging template utilizing the “Live Data Mode” module within the Leica LASX software. After software‐mediated autofocus, a pre‐irradiation image was recorded, followed by 355‐nm laser micro‐irradiation. Time‐lapse imaging was performed following the field of view every 30 s for 5–10 min. Pre‐ and post‐irradiation images were taken at 1,024 × 1,024 pixel resolution, scanning at 467 Hz, taking eight 1‐µm optical sections per image with 2× averaging. Pre‐ and post‐irradiation images were stitched using an ImageJ macro and used for visualization and analysis.

#### “Spot” micro‐irradiation

Cells were prepared for imaging as described above. Cells were placed in an environmental chamber at 37°C with 5% CO_2_ attached to an Axio Observer Z1 spinning disc confocal microscope (Zeiss). Micro‐irradiation was performed using a single‐point scanning device (UGA‐42 Firefly, Rapp OptoElectronic). Single‐point regions of interest (ROI) were defined for each cell and irradiated with 100% 405‐nm laser power for 600 iterations after removal of the ND filter. The estimated power delivered per ROI on average was approximately 27 J/m^2^. ROI *x*–*y* co‐ordinates were recorded and used for subsequent image analysis. A predefined imaging template was used within the Zen Blue acquisition software. A pre‐irradiation image was recorded, followed by 405‐nm irradiation. A time lapse was subsequently performed every 5 s for 10 min. Hardware autofocus (Definite Focus, Zeiss) was used to ensure focus was maintained throughout the time lapse and was applied every 70 frames. To avoid image acquisition during laser micro‐irradiation, a 3‐s delay was applied from the start of micro‐irradiation and the beginning of the time lapse. Images were acquired using a C13440 camera (Hamamatsu), using a C Plan APO 64×/1.40 oil objective, acquiring 4× 0.5 µm optical sections per image with 4 × 4 binning.

#### Image analysis

Recruitment to sites of spot micro‐irradiation was quantified using CellTool by modifying analysis protocol adapted from Aleksandrov *et al* (2018). Briefly, pre‐ and post‐irradiation images were first stitched using an ImageJ macro. Maximum intensity projections of the stitched images were then taken. Individual cells were manually cropped from the original image, and a 5 x5 Gaussian blur filter was applied to minimize the impact of noise on subsequent image processing. Micro‐irradiated spots were then tracked using the spot detector /track module within CellTool. Recruitment was calculated as the difference between the average intensity in the recruitment region and in a nearby region, multiplied by the total area of recruitment. For negative results, where the protein of interest was not recruited, ROI co‐ordinates were imported to CellTool and the maximum recruitment within the static ROI was determined, as described above.

#### Drug treatment

PARP inhibitors olaparib (S1060; Selleck Chem) and talazoparib (S7048; Selleck Chem) and PARG inhibitor PDD00017273 (5952; Tocris Bioscience) were used at a final concentration of 5 µM, 50 nM and 0.3 µM, respectively, and were added to the cells 1 h prior to and during micro‐irradiation. Transcription inhibitors were employed as follows: α‐amanitin (20 µg/ml) for 8 h; DRB (100 µM) for 2 h; and actinomycin D (5 nM and 2.5 µM) for 40 min prior to and for the entire duration of micro‐irradiation. For RNase treatment, cells were first washed with warm PBS and permeabilized with Tween‐20 (1% (v/v)) in PBS for 5 min followed by treatment with 1 mg/ml RNase A (Thermo) for 10 min at room temperature (RT). Following the respective treatments, cells were micro‐irradiated and imaged immediately.

### Immunofluorescence

Cells grown on coverslips were washed twice with cold PBS and fixed with 4% paraformaldehyde (sc‐281692, Santa Cruz) in PBS for 15 min at RT. After fixation, cells were washed twice with PBS and permeabilized with 0.5% (v/v) Triton X‐100 (in PBS) for 15 min at RT, washed twice with PBS and blocked for at least 1 h in antibody dilution buffer (1× PBS containing 5% normal donkey serum, 0.1% (v/v) fish skin gelatine, 0.1% (v/v) Triton X‐100, and 0.05% (v/v) Tween‐20). Incubation with the relevant primary antibody (overnight at 4°C) was followed by three washes (5 min in PBS + 0.05% (v/v) Tween‐20) and incubation with appropriate fluorescently labelled secondary antibody (60 min, RT). Coverslips were washed three times (5 min in PBS + 0.05% (v/v) Tween‐20), stained with DAPI (Sigma; 1 µg/ml in PBS, 5 min) and mounted using ProLong Gold antifade mounting agent (P36934; Thermo).

To measure chromatin retention of CDKL5 after oxidative DNA damage, U‐2‐OS Flp‐In T‐REx cells expressing GFP‐NLS or GFP‐NLS‐CDKL5 were grown on coverslips in media containing 1 µg/ml tetracycline. After 18 h, cells were pre–incubated with PDD00017273 (0.3 µM; “PARGi”) either in the absence or presence of PARP inhibitor olaparib (15 µM) for 60 min before exposing the cells to hydrogen peroxide (H1009; Sigma; 500 µM) for 30 min. Cells were then washed twice with cold PBS (containing 0.3 µM PARGi) and pre‐extracted in cold 0.2% (v/v) Triton X‐100 (in PBS containing 0.3 µM PARGi) for 4 min at room temperature prior to fixation as above. Imaging of fixed samples was carried out on a Leica TCS SP8 MP microscope using oil immersion objective (HPA CL APO CS2 63×/1.40 Oil). Quantification of detergent‐insoluble anti‐GFP signal (excluding nucleolar GFP signal) from > 150 cells per sample per repeat was done using Fiji ImageJ‐based macro. Non‐nucleolar anti‐GFP fluorescence signal was quantified in the region co‐localizing with DAPI but excluding the nucleolar region defined by fibrillarin co‐labelling. Mean nuclear GFP fluorescence was plotted relative to that in untreated WT cells. Data were plotted and analysed by GraphPad Prism v9.0.0 using one‐way ANOVA followed by the Bonferroni multiple comparison test.

To examine Ser^311^ phosphorylation of endogenous ELOA at sites of laser micro‐irradiation, 1 × 10^5^ U‐2‐OS Flp‐In T‐REx cells (wild type, CDKL5 disrupted (*CDKL5* ^Δ/Δ^) or cells pre‐depleted with indicated siRNA for 48 h) were seeded onto 8‐well chamber slides (Ibidi), 24 h prior to the experiment, in media containing 10 µM bromodeoxyuridine (Sigma). 0.3 µM PARG inhibitor (PDD00017273) was added to cells 30 min before the irradiation. Nuclei were irradiated as described previously. The cells were pre‐extracted with cold 0.2% (v/v) Triton X–100 (in PBS) for 2 min at RT and washed twice with cold PBS. Cells were fixed with 4% paraformaldehyde in PBS for 10 min at RT. After fixation, cells were washed twice with PBS and permeabilized with 0.2% (v/v) Triton X‐100 (in PBS) for 5 min at RT, washed twice with PBS and blocked for 45 min in antibody dilution buffer (1× PBS containing 5% (v/v) normal donkey serum, 0.1% (v/v) fish skin gelatine, 0.1% (v/v) Triton X‐100 and 0.05% (v/v) Tween‐20). Fixed, permeabilized cells were incubated with ELOA‐pSer^311^ antibody (0.32 µg/ml antibody pre‐mixed with 4.8 µg/ml of the corresponding non‐phosphopeptide for 12 h at 4°C overnight, followed by three washes [5 min in PBS + 0.05% (v/v) Tween‐20] and incubation with appropriate fluorescently labelled secondary antibody [60 min, RT]). Cells were washed three times (5 min in PBS+0.05% (v/v) Tween‐20), stained with DAPI (1 µg/ml in PBS, 5 min) and mounted using ProLong Gold antifade mounting agent. The buffers used in each step were supplemented with 1% (v/v) phosphatase inhibitor cocktail‐2 and PhosSTOP (Roche: 1 tablet per 10 ml). Imaging of fixed samples was carried out on a Leica TCS SP8 MP microscope using oil immersion objective (HP CL APO CS2 63×/1.40 Oil). Treatment with olaparib and DRB was done prior to irradiation as explained before. To confirm the phosphospecificity of the ELOA‐pSer^311^ antibody, fixed and permeabilized cells were (i) mock‐treated or treated with 100 U lambda‐phosphatase (NEB) overnight at 30°C prior to primary antibody incubation and (ii) incubated with 0.32 µg/ml of the phospho‐specific antibody that had been pre‐mixed with 6.4 µg/ml of the relevant phosphopeptide antigen for 12 h at 4°C.

Quantification of ELOA‐pSer^311^ to DNA damage sites was performed using a CellProfiler image analysis pipeline. After segmentation and cropping of individual nuclei, micro‐irradiation tracks delineated by PAR were segmented. Within each nucleus, the background nuclear intensity outside the segmented tracks was subtracted from the mean intensity from all detected irradiation tracks. Data were plotted and analysed by GraphPad Prism v9.0.0 using one‐way ANOVA followed by Dunnett's multiple comparison test or the unpaired t‐test with Welch's correction. The image analysis scripts are available on request.

### Recombinant protein expression and purification

*Escherichia coli* BL21 codon plus (DE3) cells transformed with expression plasmids encoding GST‐tagged CDKL5 fragments (530–730, 530–680, 530–630, 530–580), or GST alone or His_6_‐APLF were grown in Luria Broth (LB) medium containing 100 µg/ml ampicillin to *A*_600_ 0.5, followed by 0.5 mM isopropyl β‐d‐thiogalactopyranoside (IPTG) induction in early log phase for 16 h at 20°C. Cells were harvested by centrifugation at 3,500 *g*, and pellets were resuspended in lysis buffer (50 mM Tris–HCl pH 7.5, 150 mM NaCl, 5 mM DTT, 5 mM EDTA, 1 mM PMSF and 0.2 mg /ml lysozyme, 25 units Universal nuclease [Pierce™ Universal Nuclease for Cell Lysis], and left on ice for 30 min followed by brief sonication on ice [five cycles of 30 s on, 30 s off at 30% amplitude]). The homogenate was centrifuged at 20,000 *g* for 30 min at 4°C, and the clarified cell lysates were applied to respective affinity resin columns. (i) The clarified cell lysates from cells overexpressing GST‐fusion proteins were applied to glutathione‐Sepharose resin pre‐equilibrated with equilibration buffer (50 mM Tris–HCl, pH 7.5, 150 mM NaCl, 5 mM DTT and 5 mM EDTA, and 0.1% (v/v) Triton X‐100). The column was washed five times with equilibration buffer and twice with equilibration buffer without detergent. The GST‐fusion proteins were eluted with 20 mM reduced glutathione in 50 mM Tris–HCl, pH 7.5, 150 mM NaCl, 10% (v/v) glycerol, 1 mM DTT and 5 mM EDTA. (ii) The clarified cell lysates obtained from cells overexpressing His_6_‐APLF were applied to Ni‐NTA resin pre‐equilibrated with equilibration buffer (50 mM Tris–HCl, pH 7.5, 150 mM NaCl and 5 mM DTT, 0.1% (v/v) Triton X‐100 and 10 mM imidazole). The column was washed five times with equilibration buffer and twice with equilibration buffer without detergent. The His_6_‐APLF was eluted using 300 mM imidazole in 50 mM Tris–HCl, pH 7.5, 150 mM NaCl, 10% (v/v) glycerol and 1mM DTT. Eluted proteins were dialysed overnight at 4°C in sucrose buffer (50 mM Tris–HCl, pH 7.5, 150 mM NaCl, 270 mM sucrose, 0.1 mM EGTA, 0.03% (v/v) Brij‐35 and 0.1% (v/v) β‐mercaptoethanol). The proteins were concentrated, snap‐frozen and stored at −80°C for further use.

### *In vitro* poly(ADP‐ribose) binding assay

Serial dilutions (10, 5, 2.5 and 1.25 µg) of GST, GST‐CDKL5 fragments and His_6_‐APLF were dot–blotted onto an activated nitrocellulose membrane under low vacuum conditions. The membranes were dried and stained with Ponceau S to check loading. The membrane was washed and blocked with 5% (w/v) skimmed milk powder in PAR‐binding buffer (20 mM Tris–HCl, pH 7.5, and 50 mM NaCl) for 1 h prior to incubation with 50 nM synthetic PAR (Trevigen; in blocking buffer, 45 min, RT). The membrane was washed twice with PAR‐binding buffer followed by incubation with primary antibodies (rabbit anti‐PAR polyclonal; Trevigen, 1:5,000 in blocking buffer, 4°C, overnight) and secondary antibodies (goat anti‐rabbit HP‐conjugated; Thermo, 1:5,000 in milk, 1 h, RT). PAR‐binding buffer was used to rinse the membrane three times after each antibody incubation. The membrane was developed using SuperSignalTM West Pico PLUS Chemiluminescent Substrate (Thermo), and the resulting signal was captured on an X‐ray film.

### Immunoprecipitation: CDKL5 binding to PAR in cells

U‐2‐OS Flp‐In T‐REx cells stably expressing CDKL5 were mock‐treated or treated with H_2_O_2_ (500 µM; 30 min) in the presence of PDD00017273 (0.3 µM). CDKL5 was immunoprecipitated from 2 mg extract using anti‐CDKL5 antibody (S957D); sheep IgG (31243, Thermo) was used as control. Cells were lysed in 50 mM Tris–HCl (pH 7.4) buffer containing 0.27 M sucrose, 150 mM NaCl, 1% (v/v) Triton X‐100, 0.5% (v/v) Nonidet NP‐40 and 0.1% (v/v) 2‐mercaptoethanol supplemented with a protease inhibitor cocktail (cOmplete™, EDTA‐free Protease Inhibitor Cocktail), benzonase (Novagen, 50 U/ml), 10 ng/ml microcystin‐LR (33893; Sigma), phosphatase inhibitor cocktail‐2 (P5726; Merck) at 1% (v/v), 0.3 µM PARGi PDD00017273 (5952; Tocris bioscience) and 5 µM PARPi olaparib. Extracts were then incubated for 30 min at 4°C and clarified by centrifugation at 17,000 *g* in a refrigerated centrifuge. Clarified extracts were pre‐cleared using DynaBeads Protein G (10003D; Life Technologies) conjugated with sheep IgG isotype control using the manufacturer's protocol, for 45 min at 4°C. Pre‐cleared extracts were used to immunoprecipitate CDKL5 using sheep polyclonal CDKL5 antibodies or sheep IgG isotype control with DynaBeads Protein G. Approximately 2 µg of anti‐CDKL5/sheep IgG isotype control was linked to beads to perform pull down from 2 mg of pre‐cleared extracts for 2 h at 4°C. Alternatively, pre‐cleared extracts (2 mg) were incubated for 4 h at 4°C with 2 µg of pan‐ADP‐ribose binding reagent (MABE1016; Merck) or normal rabbit IgG (2729S; Cell Signaling) conjugated to DynaBeads Protein G. Beads were washed three times with lysis buffer and twice in cold PBS before boiling at 95°C in LDS–PAGE sample buffer (Thermo Fisher) containing 5% (v/v) 2‐mercaptoethanol. Samples were resolved in 4–12% Bis–Tris SDS–PAGE gradient gels (NuPAGE, Thermo Fisher). Input lysates or immunocomplexes were analysed by Western blotting using sheep polyclonal anti‐CDKL5, pan‐ADP‐ribose binding reagent and anti‐GAPDH (14C10; Cell Signaling) antibodies. Antibodies were diluted in 5% (w/v) skimmed non‐fat dry milk in TBS–Tween‐20 (0.2% v/v). Membranes were incubated overnight at 4°C or 2 h at RT with the relevant antibodies, then washed. Membranes were then incubated with recombinant protein G‐HP (1: 2,500; ab7460) for 1 h at RT. The membrane was developed using SuperSignalTM West Pico PLUS Chemiluminescent Substrate (Thermo), and the resulting signal was captured on an X‐ray film.

### Phosphoproteomic screening for nuclear substrates of CDKL5

Twenty 15‐cm plates of CDKL5‐disrupted U‐2‐OS cells (*CDKL5* ^Δ/Δ^) expressing CDKL5^NLS^ WT or CDKL5^NLS^ K^42^R were grown to around 70% confluence, treated with H_2_O_2_ (500 µM for 15 min), washed twice with PBS and harvested in 4 ml of ice‐cold solution containing 20% (v/v) TCA, 80% (v/v) acetone and 0.2% (w/v) DTT, transferred into 5‐ml Eppendorf tubes and stored at −20°C overnight. Samples were then centrifuged twice at 20,000 *g*, −10°C for 20 min, and supernatants were then discarded. Pellets were resuspended with 2 ml ice‐cold 80% (v/v) acetone and then centrifuged again at 20,000 *g* at −10°C for 30 min. After removing the supernatants completely, pellets were left to air‐dry for 10 min.

TCA/acetone‐precipitated pellets were resuspended in 500 µl 8 M urea, 50 mM AmBiC, 1% (v/v) phosphatase inhibitor cocktail‐2, 0.1% (v/v) microcystin, pH 8.0 and benzonase at a concentration of 0.2% (v/v) and incubated for 15 min at room temperature, and finally lysed using a Bioruptor sonicator. Lysates were centrifuged at 20,000 *g* for 30 min at RT and stored at −80°C for further mass spectrometric analysis. Five independent biological replicates were carried out, on different days.

Protein concentrations were determined using a BCA assay kit, and the absorbance was measured at 560 nm. A total of 5 mg protein from each sample was reduced with 5 mM DTT at 45°C for 30 min, alkylated with 10 mM iodoacetamide at room temperature in the dark for 20 min, quenched by addition of 5 mM DTT, digested with Lys–C (1:200 (w/w), LysC:protein) for 4 h at 30°C and then diluted with 50 mM ammonium bicarbonate to 1.5 M final urea concentration, followed by trypsin digestion (1:50 (w/w), trypsin: protein) at room temperature overnight. 1% TFA (v/v) was added to stop the digestion. The acidified digests were centrifuged at 10,000 *g* for 10 min. The collected supernatants were then desalted on 200 mg Sep–PAK tC18 cartridges, and the eluents were dried by speed vacuum centrifugation (Thermo). Desalted peptides were resuspended in 1 ml of 2 M lactic acid and 50% (v/v) acetonitrile (ACN) and centrifuged at 15,000 *g* for 20 min. Supernatants were transferred to an Eppendorf tube containing 18 mg of titanium dioxide (TiO_2_) beads (GL sciences, Japan) and vortex‐mixed for 1 h at room temperature. The TiO_2_ beads were washed two times (10 min per wash) with 2 M lactic acid and 50% (v/v) ACN followed by three washes with 0.1% (v/v) TFA and 50% (v/v) ACN. Phosphopeptides were eluted twice with 150 µl of 10% (v/v) ammonia solution (NH_4_OH) and were finally eluted with 150 µl of 50% (v/v) ACN and 5% (v/v) ammonia solution (NH_4_OH). The combined eluent was dried with vacuum centrifugation and then cleaned up using in‐house‐made C18 StageTips (3 M Empore™). 1% of each TiO_2_‐enriched sample was analysed by mass spectrometry prior to following processes.

TMT10plex labelling was performed according to the manufacturer's protocol using the TMT Labeling Kit. Briefly, the TiO_2_‐enriched sample was resuspended into 100 µl of 100 mM TEAB. A total of 0.4 mg of each TMT tag was used for labelling each sample. After 1‐h incubation, 2 µl of each labelled sample was diluted with 18 µl of 0.1% formic acid and was then checked for TMT labelling efficiency. After checking the labelling efficiency, each TMT‐labelled sample was quenched by incubation with 8 µl of 5% (w/v) hydroxylamine for 30 min at RT. The quenched samples were then mixed and fractionated with high pH reverse‐phase C18 chromatography using the Ultimate 3000 high‐pressure liquid chromatography system (Dionex) at a flow rate of 569 µl/min using two buffers: buffer A (10 mM ammonium formate, pH 10) and buffer B (80% ACN, 10 mM ammonium formate, pH 10). Briefly, the desalted TMT‐labelled samples were resuspended in 200 µl of buffer A (10 mM ammonium formate, pH10) and fractionated on a C18 reverse‐phase column (4.6 × 250 mm, 3.5 µm, Waters) with a gradient as follows: 3% buffer B to 12.5% buffer B in 5 min, 12.5% to 40% buffer B in 35 min, 40% B to 60% B in 15 min, 60% B to 100% B in 5 min, 100% for 5 min, ramping to 3% B in 5 min and then 3% for 10 min. A total of 60 fractions were collected and then concatenated into 20 fractions, which were further desalted over C18 StageTips and speed vacuum‐dried prior to LC–MS/MS analysis.

#### LC–MS/MS mass spectrometry

LC–MS/MS analysis was performed with an Orbitrap Fusion Lumos (Thermo), with a Thermo Dionex Ultimate 3000RSLC nano‐liquid chromatography instrument. Peptide concentration from each fraction was quantified by Nanodrop, samples were dissolved in 0.1% formic acid, and 1 µg of each fraction was loaded on C18 trap column with 3% (v/v) ACN and 0.1% (v/v) TFA at 5 µl/min flow rate. Peptides were separated over an EASY‐Spray column (C18, 2 µm, 75 µm × 50 cm) with an integrated nano‐electrospray emitter (flow rate 300 nl/min). Peptide separation was done over 180 min with a segmented gradient applying following buffer system: buffer A: 0.1% (v/v) formic acid; and buffer B: 80% (v/v) acetonitrile and 0.08% (v/v) formic acid. The first seven fractions started from 6 to 35% buffer B for 120 min (note: the following seven fractions started from 8% and the last six fractions started from 10%), 35–45% buffer B for 30 min, 45–95% buffer B for 5 min and 95% buffer B for 5 min. Eluted peptides were analysed on an Orbitrap Fusion Lumos (Thermo Fisher Scientific, San Jose, CA) mass spectrometer. Spray voltage was set to 2.2 kV, RF lens level was set at 30%, and ion transfer tube temperature was set to 275°C. The Orbitrap Fusion Lumos was operated in positive ion data‐dependent mode with HCD fragmentation and orbitrap detector for all precursor fragments for reporter ion quantitation. The mass spectrometer was operated in data‐dependent Top speed mode with 3 s per cycle. The full scan was performed in the range of 350–1,500 *m*/*z* at nominal resolution of 120,000 at 200 *m*/*z* and AGC set to 4 × 10^5^ with maximal injection time of 50 ms. The MS2 scan was set with an isolation width of 1.2 *m*/*z* with no offset, followed by selection of precursors above an intensity threshold of 5 × 10^4^ for higher‐energy collisional dissociation (HCD)–MS2 fragmentation with 38% normalized collision energy. Dynamic exclusion was set to 60 s. Monoisotopic precursor selection was set to *peptide*, and maximum injection time was set to 120 ms. Charge states between 2 and 7 were included for MS2 fragmentation and analysis of fragment ions in the orbitrap using 50,000 resolving power with auto normal range scan starting from *m*/*z* 100 and AGC target of 5 × 10^4^.

#### Global phosphoproteomic data analysis

Mass spectrometric raw data were searched against the UniProt database (*homo sapiens*, including protein isoform sequences, 42,326 entries, downloaded 05/04/2018 from www.uniprot.org) using MaxQuant (version 1.6.3.4) (Tyanova, Temu *et al*, 2016). Variable modifications were set to: oxidation of methionine, phosphorylation of serine, theonine and tyrosine, deamidation of asparagine, carbamylation of the peptide N‐terminus and acetylation of the protein N‐terminus. Fixed modification was set as carbamidomethylation of cysteine. False discovery rate threshold for peptide identification was set to 5%. Quantitative result data were analysed using an in‐house R (Version 4.0.1) (R‐Core‐Team, 2020) analysis pipeline (Script Files S1–S4). In brief, the intensities of peptides with more than one observation within a single sample fraction were averaged. Peptides quantified in several fractions were averaged in each respective fraction independently to avoid reporter ion quantification bias caused by differences in precursor co‐isolation populations between different sample fractions. Data were normalized and calibrated using variance stabilizing normalization (VSN) (Huber *et al*, 2002, 2003). Statistical testing was carried out using linear models for microarrays (*limma*) (Ritchie *et al*, 2015). under application of robust hyperparameter estimation (Phipson *et al*, 2016).

To define putative CDKL5 substrates, mass spectrometric data (Table EV1) were subjected to the volcano plot analysis shown in Fig 4B, which revealed 37 phosphopeptides (31 unique sequences) that were higher in abundance in the CDKL5 WT samples compared with the KD samples; this group clustered away from the bulk of phosphopeptides, and all the phosphopeptides within this cluster had *P* < 0.0005. 22 of these 37 phosphopeptides had a single, unique phosphorylation site (≥ 75% PTM score probability) and were assigned as peptides of interest (Fig 4C). Additionally, phosphorylation sites were flagged if they had a PTM score probability of ≥ 0.994, corresponding to a false localization rate (FLR) of 1% (Ferries *et al*, 2017). Peptide metadata were extracted from the following databases: UniProt (gene ontology (GO) data, downloaded from www.uniprot.org on 05/04/2020) (UniProt, 2019) and STRING (version 11.0, downloaded from https://string–db.org/) (Szklarczyk *et al*, 2019). GO terms and protein–protein interaction networks were analysed using R (Script File S3) under application of Fisher's exact test against a background of all unique leading razor proteins within this study (5,985 proteins as assigned by MaxQuant from the identified peptides; isoforms of the same protein were not counted as distinct proteins). GO terms were deemed significant if they had a *P*‐value of ≤ 0.01 and at least three proteins from the group of 25 unique proteins with significant peptides exhibiting the respective GO term (foreground; 24 proteins with more and one protein with a fewer phosphopeptides in CDKL5 WT samples compared with KD). The protein–protein interaction network of the 24 unique proteins containing a phosphopeptide higher in abundance in CDKL5 WT samples compared with KD was analysed by R (Script File S4) using the STRING database with an interaction score threshold of 150. Following R packages were used: ggplot2 (Wickham, 2016), reshape2 (Wickham, 2007), vsn (Huber *et al*, 2002), *limma* (Ritchie *et al*, 2015), seqinR (Charif & Lobry, 2007), plyr (Wickham, 2011), stringr (Wickham, 2019), ggrepel (Slowikowski, 2020), ggpointdensity (Kremer, 2019), wesanderson (Ram & Wickham, 2018), extrafont (Winston, 2014), scales (Wickham & Seidel, 2020), matrixStats (Bengtsson, 2020), GO.db (Carlson, 2020), STRINGdb (Szklarczyk *et al*, 2019), igraph (Csardi & Nepusz, 2006), gtools (Warnes *et al*, 2021, https://CRAN.R‐project.org/package=gtools) and ggnetwork (Briatte, 2020). Session information is listed in Text File S1. The mass spectrometric nuclear phosphoproteomic data have been deposited to the ProteomeXchange Consortium via the PRIDE (Perez‐Riverol *et al*, 2019) partner repository with the dataset identifier PXD022916.

Data analysis Script Files (S1–S4), Session information text file S1, and the relevant database links can be downloaded from the Zenodo link: https://doi.org/10.5281/zenodo.5095151.

### Extracted ion chromatogram (XIC) analysis

HEK293 cells were transfected using the calcium phosphate transfection protocol as previously described (Munoz *et al*, 2018). Cells were seeded at a confluence of 20–30% in 15‐cm plates and 24 h later were co‐transfected with 5 µg DNA for each plasmid. Cells were kept with the transfection mixture for 24 h and then either mock‐treated or incubated with H_2_O_2_ at a final concentration of 500 µM for 15 min. Plates were then washed twice with phosphate saline buffer and lysed in a 50 mM Tris–HCl (pH 7.4)‐based buffer containing 0.27 M sucrose, 150 mM NaCl, 1% (v/v) Triton X‐100, 0.5% (v/v) Nonidet NP‐40 and 0.1% (v/v) 2‐mercaptoethanol. Lysis buffer was freshly supplemented with a protease inhibitor cocktail (cOmplete™, EDTA‐free Protease Inhibitor Cocktail), benzonase at 50 U/ml, microcystin‐LR at 10 ng/ml final concentration, phosphatase inhibitor cocktail‐2 (Merck) at 1% (v/v), olaparib (10 µM) and PDD00017273 (2 µM). Lysates were incubated for 30 min at 4°C and clarified by centrifugation at 20,000 *g* at 4°C.

For extracted ion chromatography (XIC) analysis, approximately 25 µl (settled volume) of FLAG‐M2 agarose (Sigma‐Aldrich; F1804) beads was mixed with the following amounts of lysate for 2–3 h at 4°C: 10 mg of crude lysate for EP400 samples, 6 mg for Elongin A and 2.5 mg for TTDN1. Precipitates were then extensively washed with lysis buffer and finally once in cold PBS. Samples were then denatured in 25 µl LDS–PAGE sample buffer (Thermo Fisher) supplemented with 5% (v/v) 2‐mercaptoethanol and then incubated at 95°C for 5 min. All the immunoprecipitations were done in triplicates using lysates from independent replicate transfections. Samples were resolved in 4–12% Bis–Tris SDS–PAGE gradient gels (NuPAGE, Thermo Fisher), and relevant bands were excised and further processed for mass spectrometry as detailed below. Protein bands excised from the gel were destained, and proteins were digested with trypsin/LysC as described in Munoz *et al* (2018). Peptides were labelled with TMT10plex (Thermo Fisher) according to the manufacturer's protocol, omitting the TMT131 label. Deviating from the protocol described in Munoz *et al* (2018), labelling reaction was stopped using a 5% (w/v) hydroxylamine (Sigma) solution. After labelling, the peptides were freeze‐dried and stored at −80°C until required.

Peptides were resuspended in 2% (v/v) acetonitrile (Merck) and 0.1% (v/v) formic acid (Merck), incubated for 15 min in an ultrasonic bath (VWR) and afterwards transferred into glass autosampler vials (Waters). Peptides were separated and analysed using the instrumental setup as described for the phosphoproteomic dataset. Elution of peptides was achieved by a segmented linear gradient over 120 min: initial 3 min of isocratic 3% B, followed by 3% B to 7% B in 2 min, to 25% B in 60 min, to 45% B in 30 min, to 95% B in 5 min and isocratic state at 95% B for 5 min. This was followed by a linear gradient from 95% B to 5% B within 0.5 min and column re‐equilibration for 14.5 min at 5% B. Flow rate was set to 300 nl/min. MS precursor ion scan was conducted within the Orbitrap at a resolution of 120,000 at 200 *m*/*z*. The top 15 precursors within a mass range of 350–1,500 *m*/*z* were isolated in the quadrupole (0.7 Da isolation window, AGC target: 4 × 10^5^, max. injection time 50 ms) for subsequent fragmentation using HCD (38% normalized collision energy, AGC target: 5 × 10^4^, max. injection time 120 ms) and analysed in the Orbitrap with a resolution of 50,000 at 200 *m*/*z*. Analysed peptides were dynamically excluded after their first measurement from reanalysis for a duration of 60 s. Data were recorded in profile mode. In case of the file “Ivan_EP400–TMT.raw”, an inclusion list of 628.3314 *m*/*z* (TMT‐labelled phosphopeptide SSPVNRPSpSATNK) was set. Orbitrap‐run metadata were extracted using the MARMoSET R package as described on their GitHub page (https://github.molgen.mpg.de/loosolab/MARMoSET, accessed 28/11/2020) (Kiweler *et al*, 2019).

Mass spectrometric raw data were searched using MaxQuant (version 1.6.3.4). Variable and fixed modifications with the exclusion of carbamylation of the peptide N‐terminus, FASTA and FDR thresholds were set as described above for the phosphoproteomic dataset. Data were analysed using in‐house written R‐scripts (see Data availability), which were modified from Munoz *et al* (2018). In brief, all TMT reporter intensities of the identified peptides of the respective protein were normalized using VSN and intensities were statistically tested using a t‐test with subsequent Bonferroni correction of the significance threshold of α = 0.05. In cases where phosphopeptides were detected multiple times, the median intensity within each respective TMT channel was taken for statistical testing. Peptides with a *P*‐value < 0.0125 (4 tests: EP400, TTDN1) or < 0.00833 (6 tests: ELOA) were considered significant. Session information is listed in Text File S2. The mass spectrometry‐extracted ion chromatography data have been deposited to the ProteomeXchange Consortium via the PRIDE (Perez‐Riverol *et al*, 2019) partner repository with the dataset identifier PXD022975. Data analysis Script Files S5–S8 and Session information text file S2, with links to the relevant databases can be downloaded from the Zenodo link: https://doi.org/10.5281/zenodo.4311494.

### U–2–OS FokI transcription reporter assay

#### Recruitment of CDKL5 to FokI‐induced DSBs

U‐2‐OS 265 transcription reporter cells (Tang, Cho *et al*, 2013) were infected with retroviruses as explained above, to stably express GFP alone or GFP‐CDKL5 with an exogenous nuclear localization signal. The cells were seeded onto an 8‐well chamber slides 24 h before the experiment. On the day of the experiment, cells were mock‐treated or treated with 1 µ/ml doxycycline hyclate (D9891‐G; Sigma‐Aldrich) for 3 h, to induce reporter gene transcription. To prolong the retention of CDKL5 at FokI‐induced DSBs, inhibitors of PARG (0.3 µM) and ATM (10 µM) were added in the same media for 30–45 min before adding 1 µM Shield‐1 ligand (632189; Clontech Laboratories UK Ltd) and 1 µM 4‐hydroxytamoxifen (4‐OHT; H7904–5MG, Sigma) to induce mCherry‐FokI expression and subsequent DSB induction. Just 15 min after DSB induction, cells were live‐imaged on a Leica TCS SP8 MP microscope using water immersion objective (HAP CL APO CS2 63×/1.40 water) supplemented with 5% CO_2_ maintained at 37°C. The fields of cells were quickly scanned manually to image the cells showing co‐localization of mCherry‐FokI foci with GFP signal. Imaging was done for 10 min, making total time lapse of not more than 25 min post‐DSB induction. Due to the CDKL5 transience at DSBs, the experiment was done in multiple technical replicates on the same day by employing staggered drug treatments, to ensure image collection of several fields, and in three independent biological replicates.

#### MS2 foci

U‐2‐OS 263 IFII transcription reporter cells (Tang *et al*, 2013) transfected with relevant siRNA were seeded onto eight‐well chamber slides and treated with 1 µM Sheild1 (632189, Clontech Laboratories UK Ltd) and 1 µM 4–hydroxytamoxifen (4–OHT; H7904–5MG, Sigma) for 3 h to induce mCherry–FokI expression and 1 mg/ml doxycycline hyclate (D9891–G; Sigma‐Aldrich) for an additional 3 h to induce reporter gene transcription. Cells were fixed with 4% paraformaldehyde in PBS (sc–281692; Santa Cruz), washed three times with PBS, permeabilized with 0.2% (v/v) Triton X‐100/PBS for 3 min at RT, washed and stained with DAPI (1 µg/ml in PBS, 5 min, RT) and mounted using ProLong Gold antifade mounting agent (P36934; Thermo). Imaging of fixed samples was carried out on a Leica TCS SP8 MP microscope using oil immersion objective (HAP CL APO CS2 63×/1.40 Oil). The number of transcription‐positive cells was scored manually from a total of 150–200 cells per variable in each independent repeat. Data were plotted and analysed by GraphPad Prism v9.0.0 using two‐way ANOVA followed by the Bonferroni multiple comparison test.

#### RNA isolation, reverse transcription and quantitative real‐time PCR

RNA was extracted from 1.2 × 10^6^ cells using E.Z.N.A.^®^ Total RNA Kit I (R6831–01) following the manufacturer's protocol. cDNA was synthesized from 1 µg RNA using iScript cDNA Synthesis Kit (170–8891). qPCR was performed using a CFX384 real‐time PCR system (Bio‐Rad), relevant primers with 2% (around 20 ng) of the cDNA and TB Green™ Premix Ex Taq™ II (Tli RNase H Plus; RR820L; Takara) with two repeats for each PCR. The ΔΔC_t_ method was used for evaluation. GAPDH gene was used as a housekeeping gene for normalization. Data were analysed in Excel software (Microsoft) and plotted in GraphPad Prism v9.0.0 software. Statistical significance was determined by two‐way ANOVA followed by Dunnett's multiple comparison test. Primers used are listed in Table EV2.

### Gene silencing in response to I‐PpoI‐mediated DSB induction

2 × 10^5^ cells/ml/well U‐2‐OS‐pEP15 cells (DOX‐inducible ER‐I‐PpoI expressing stable cell line) were seeded into six‐well plates for siRNA transfection. Cells were transfected with 40 nM siControl or siCDKL5‐b (Dharmacon) using Interferin transfection reagent (Polyplus). Next day, 24 h after siRNA silencing, cells were transfected with 3 µg pcDNA5D empty, pCDNA5D‐CDKL5 WT or pCDNA5D‐CDKL5‐K^42^R KD plasmids using jet‐PEI transfection reagent (Polyplus). 16 h prior to the first 4‐OHT treatment, 1 µg/ml doxycycline hyclate (Sigma‐Aldrich) was applied to induce the expression of I‐PpoI endonuclease. The following day, 1 µM 4‐OHT (Sigma‐Aldrich) treatment was used at different time points (2, 4 and 8 h) to facilitate the nuclear translocation of I‐PpoI. 48 h after siRNA transfection, cells were collected and destined for RNA and gDNA isolation. For RNA isolation, ReliaPrep RNA Tissue Miniprep System (Promega), but for gDNA isolation, ReliaPrep gDNA Tissue Miniprep System (Promega), is used according to the manufacturer's instructions. cDNA reverse transcription was performed with Applied Biosystems TaqMan Reverse Transcription Reagents (Thermo Fisher Scientific) according to the manufacturer's instructions. qPCR experiments were performed on RotorGene Q qPCR machine. For data evaluation, DDCt method was used. Data were plotted and analysed by GraphPad Prism v9.0.0 using two‐way ANOVA followed by Tukey's multiple comparison tests.

## Author contributions

TK and IM executed most of the experiments. HZ performed mass spectrometric global phosphoproteomic experiments (IM prepared samples). FW analysed the global MS dataset and executed the XIC experiments, performed the related data analysis and wrote all R‐scripts. TC helped with microscopy and the analysis of microscopy data. PA helped with microscopy experiments. MM carried out peptide kinase assays. MS made the original discovery of CDKL5 recruitment to DNA damage. MN expressed proteins in bacteria and purified them. BNB, VP and TP carried out I‐PpoI experiments. RT made cDNA clones. JR conceived the project, helped design experiments and wrote the paper.

## Conflict of interest

The authors declare no conflict of interests.

## Supporting information



Expanded View Figures PDF

Table EV1

Table EV2

Movie EV1

Movie EV2

Source Data for Expanded View

Source Data for Figure 1

Source Data for Figure 2

Source Data for Figure 3

Source Data for Figure 5

Source Data for Figure 6

Source Data for Figure 7

Source Data for Figure 8

## Data Availability

The global phosphoproteomic mass spectrometric data have been deposited in ProteomeXchange with the primary accession code PXD022916 that can be downloaded from https://www.ebi.ac.uk/pride/archive/projects/PXD022916. Data analysis Script Files (S1–S4), Session information text file S1, and the relevant database links can be downloaded from the Zenodo link: https://doi.org/10.5281/zenodo.5095151. The extracted ion chromatogram data have been deposited in ProteomeXchange with the primary accession code PXD022975 (http://www.ebi.ac.uk/pride/archive/projects/PXD022975). Data analysis Script Files (S5–S8) and Session information text file S2, with links to the relevant databases, can be downloaded from the Zenodo link: https://doi.org/10.5281/zenodo.4311494. The microscopy source data links in the relevant figure legends are kindly enabled by the Open Microscopy Environment (OMERO; https://www.openmicroscopy.org/) (Allan, Burel *et al*, 2012).
