# Supplementary material for: CDKL5 kinase controls transcription‐coupled responses to DNA damage
Source: EMBO J. 2021 Oct 4;40(23):e108271. doi: 10.15252/embj.2021108271 (PMC8634139; doi:10.15252/embj.2021108271)
Supplement: Supplementary file 1 — Expanded View Figures PDF [file EMBJ-40-e108271-s006.pdf]

Expanded View Figures

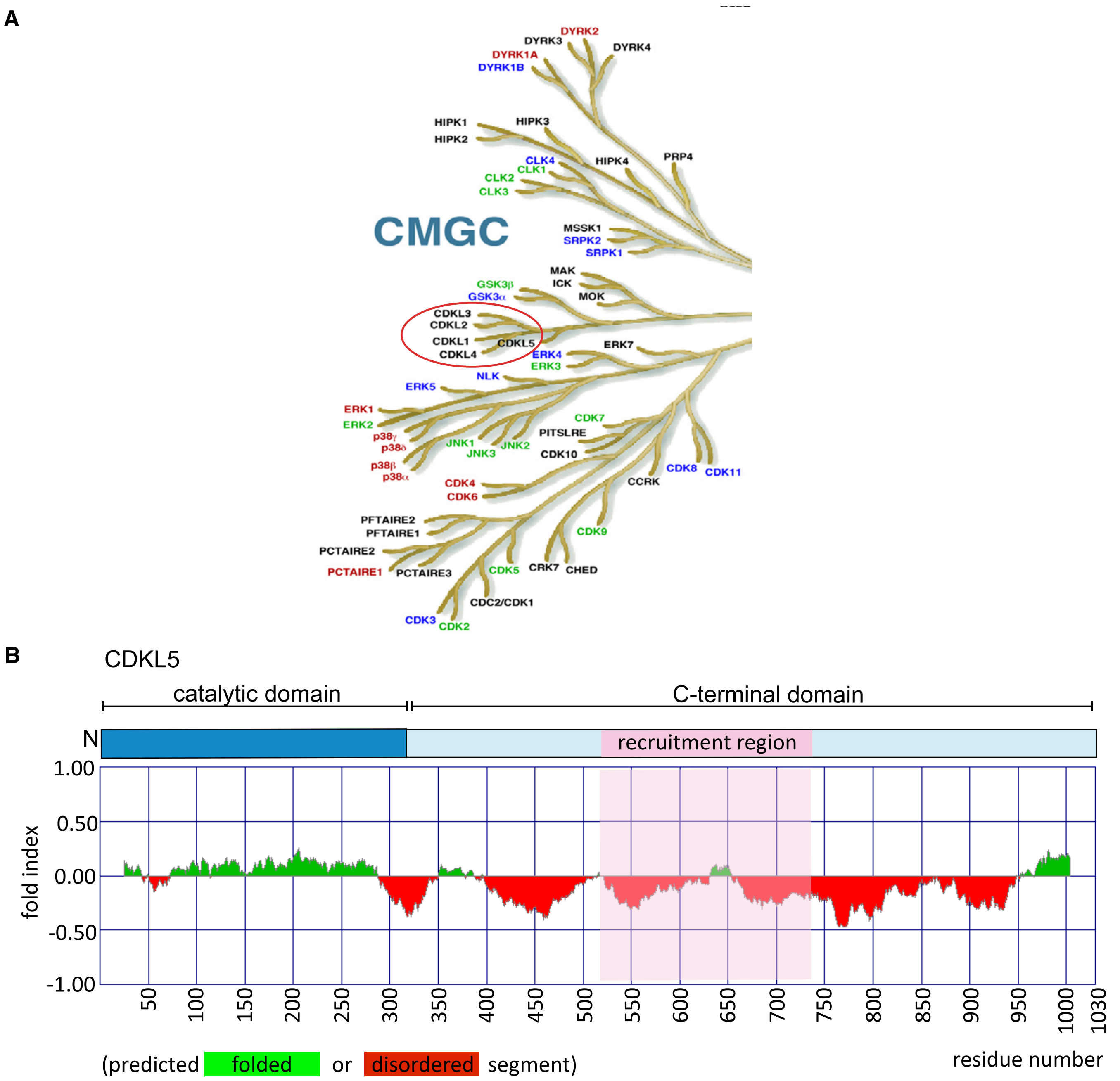

Figure EV1. CDKL5 kinase.

A The CMGC branch of the human kinome. The figure is taken from the dendrogram made by Manning and colleagues (Manning *et al*, 2002). The CDKL family of kinases that includes CDKL5 is encircled in red.

B (*Top*) Schematic diagram of CDKL5 protein. (*Bottom*) Bioinformatics analysis for CDKL5 folding using Fold index software (Prilusky *et al*, 2005). The plot shows the disorder prediction for CDKL5 protein sequence. Ordered regions are indicated in green above 0, while disordered regions are indicated in red below 0. Amino acids suggested as being folded or unfolded are depicted at the bottom of the plot. The region that mediates recruitment of CDKL5 to DNA damage sites is marked in pink.

© 2021 The Authors

Figure EV2. Restricting CDKL5 expression to the cell nucleus.

A Extracts of CDKL5-disrupted U-2-OS (Flp-In T-REx) cells (*CDKL5*$^{\Delta/\Delta}$) stably expressing CDKL5^{NLS} WT or a K^{42}R kinase-dead mutant (CDKL5^{NLS}–KD) or empty vector were subjected to Western blotting with the antibodies indicated. Two different dishes of cells are shown per condition.

B Subcellular fractionation of lysates from *CDKL5*$^{\Delta/\Delta}$ cells stably expressing CDKL5, CDKL5^{NLS} WT or CDKL5^{NLS} KD or empty vector. Lysates were fractionated to isolate proteins found in the following subcellular compartments: cytoplasmic (Cyt), membrane (Mb), nuclear (Nuc), chromatin (Ch) or cytoskeleton (Csk). Fractionated samples were resolved by SDS–PAGE and probed with antibodies shown.

C *CDKL5*$^{\Delta/\Delta}$ cells stably expressing CDKL5, CDKL5^{NLS} WT or CDKL5^{NLS} KD were subjected to indirect immunofluorescence analysis with anti-CDKL5 antibodies. Scale bar is 10 μm.

D *CDKL5*$^{\Delta/\Delta}$ cells stably expressing CDKL5^{NLS} WT or CDKL5^{NLS} KD (or empty vector) were treated with 500 μM H_2O_2 for 15 min. Samples were resolved by SDS–PAGE and probed with indicated antibodies or stained with Ponceau S to show equal loading. Rep=biological replicate.

E Peptide kinase assays to investigate CDKL5 sequence specificity. Anti-FLAG precipitates from HEK293 cells transiently expressing FLAG-tagged CDKL5 (wild-type "WT" or a K^{42}R kinase-dead "KD" mutant) were incubated with synthetic peptides corresponding to sequence around the previously reported CDKL5 phosphorylation site in MAP1S (Ser^{900}) designed specifically to investigate the effect of amino acid substitutions A^{901} on the phosphorylation of MAP1S Ser^{900}. Assays were done in the presence of [γ-^{32}P]-labelled ATP-Mg^{2+}, and peptide phosphorylation was measured by Cerenkov counting. Phosphorylation of the control wild-type MAP1S peptide is taken as 100% (*). The data are represented as mean ± SEM from three independent experiments. The RPXSA motif is shaded in blue, and amino acid substitutions compared with the wild-type MAP1S Ser^{900} peptide are shown in red.

Source data are available online for this figure.

© 2021 The Authors

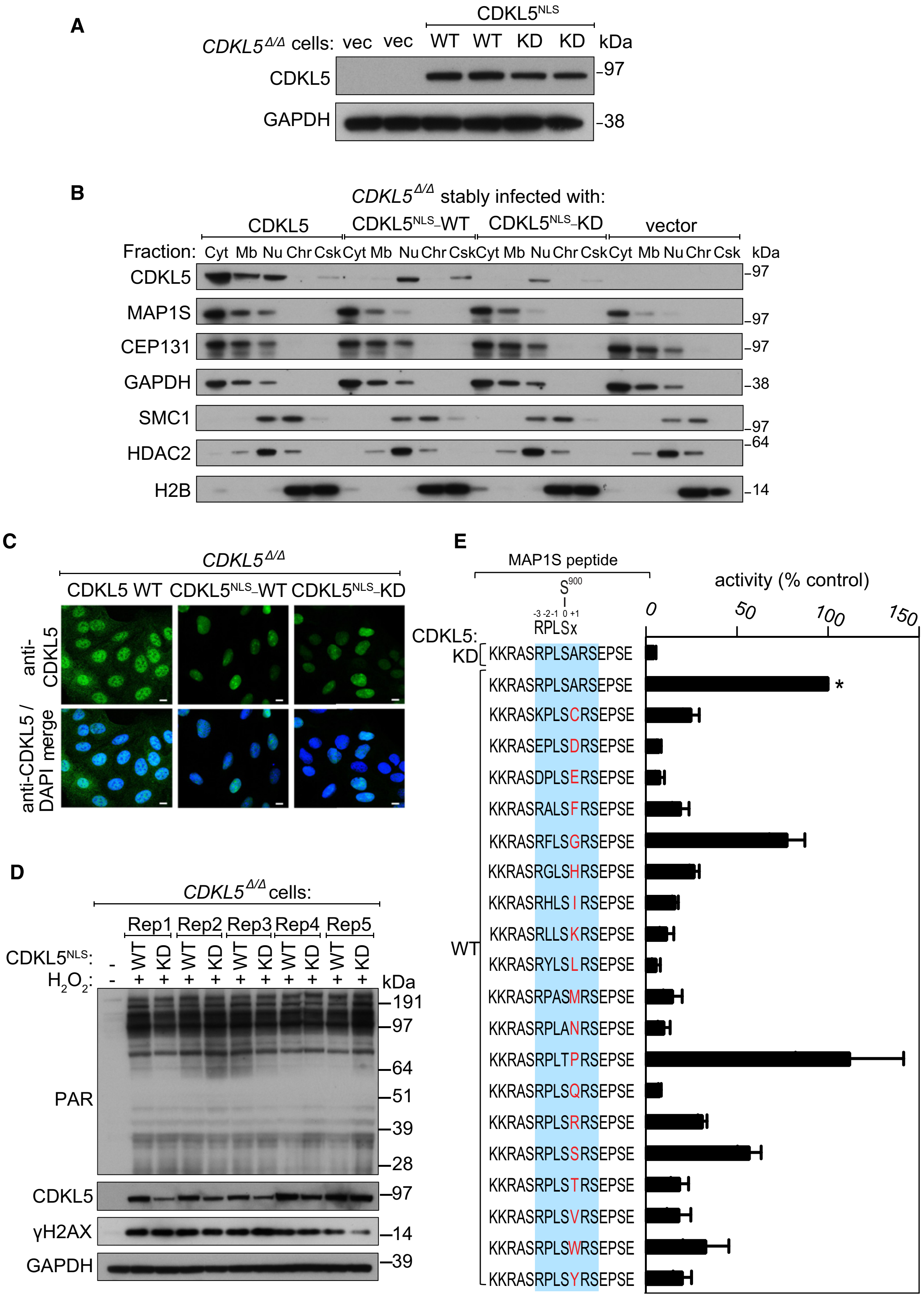

Figure EV2.

© 2021 The Authors

Figure EV3. Phosphoproteomic data quality control.

A Left: Normal Q–Q plot of the raw TMT intensity data with large deviations from a normal distribution as seen from datapoints not following the indicated line in the plot. Right: Q–Q plot of the TMT intensity data after VSN transformation. Only minor deviations from the line indicates the transformed data follow a normal distribution to a satisfactory degree. The hypervariable datapoints in the upper quantiles are controlled by the application of the robust implementation of the empirical Bayes algorithm used by *limma* (Phipson *et al*, 2016) and implemented in the analysis scripts.

B Standard deviation plotted against the intensity rank of the VSN-transformed TMT data. Red line indicates the mean standard deviation. Line is approximately horizontal, indicating that the variance is not overly dependent on intensity rank and suggests a successful VSN transform.

C Boxplot of intensity distribution in each TMT channel. No obvious discrepancy between the median values of the individual channels indicates a successful calibration by VSN and no introduction of an obvious intensity bias for any experimental group. The central band of the boxplot indicates the median value, while the hinges represent the first and third quartile (bottom and top of boxplot, respectively). The whiskers extend to the largest/smallest (upper or lower whisker, respectively) datapoint not further than 1.5 times the interquartile range from their respective hinge. The experiment was conducted using five biological replicates of $CDKL5^{NLS}$ WT (WT, red) and $CDKL5^{NLS}$ KD (KD, blue) where each TMT channel represents a single biological replicate from the respective group.

Source data are available online for this figure.

© 2021 The Authors

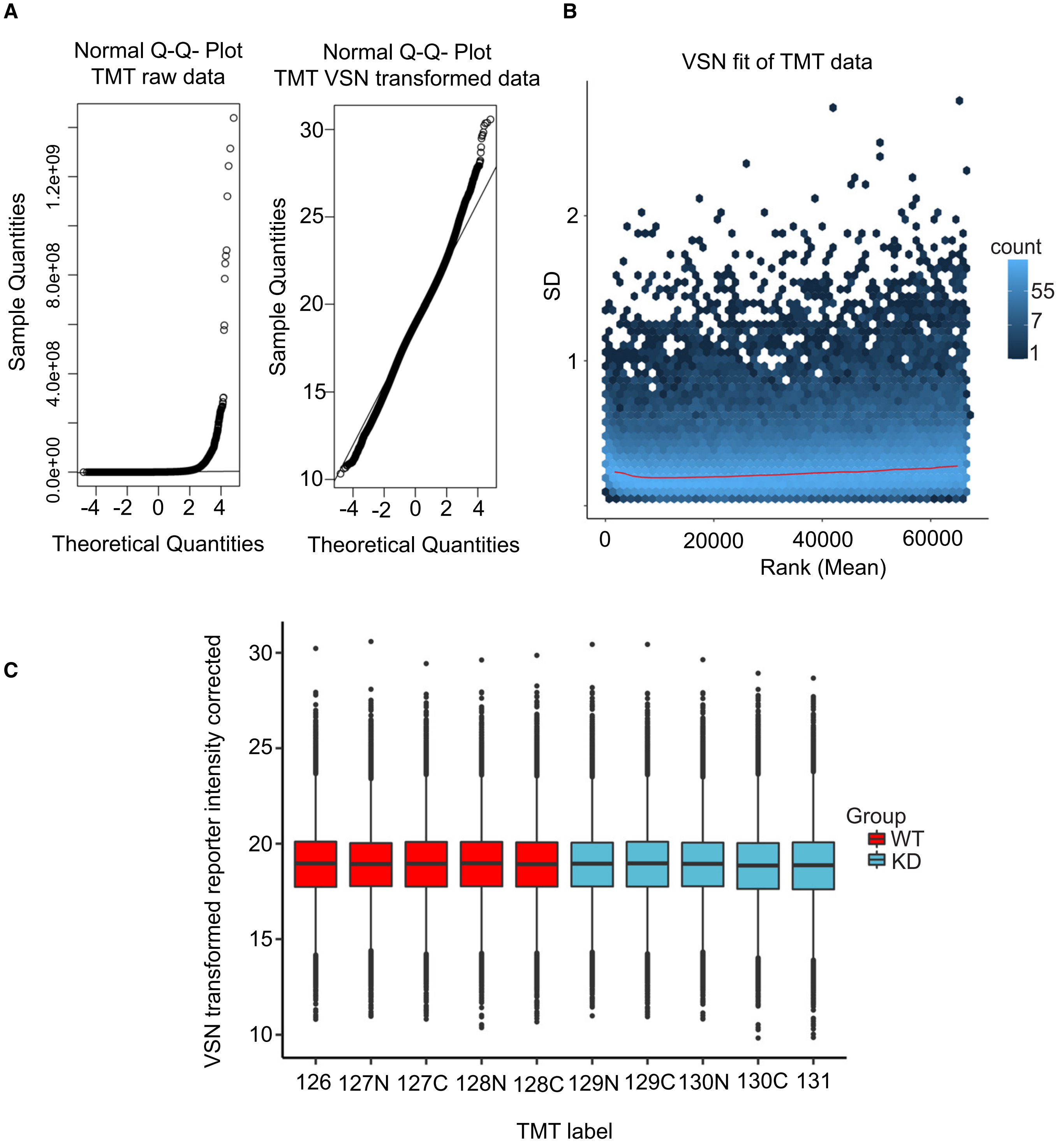

Figure EV3.

 © 2021 The Authors

Figure EV4. Recruitment of CDKL5 substrates to DNA damage sites.

A–C BrdU-sensitized U-2-OS (Flp-In T-REx) cells stably expressing GFP-tagged forms of the proteins indicated were pre-incubated with (A) olaparib (PARPi; 5 μM) or PD00017273 (PARGi; 0.3 μM, 1 h), (B) α-amanitin (20 μg/ml, 8 h) or (C) DRB (100 μM, 2 h) prior to line micro-irradiation (355 nm) and time-lapse imaging. One of three independent experiments is shown. Scale bar is 10 μm.

D BrdU-sensitized U-2-OS (Flp-In T-REx) cells were subjected to nuclear line micro-irradiation (355 nm). Cells were fixed and then mock-treated (con) or treated with lambda-phosphatase prior to incubation with the primary antibodies indicated. Alternatively, ELOA-pSer^{311} phosphopeptide was included during incubation with the primary antibodies before indirect immunofluorescence analysis. Quantification of ELOA-pSer^{311} signal at the laser tracks is shown. Data represent mean ± SD of total pELOA Ser^{311} intensities in different biological replicates as indicated (*n*). For simplicity, only intensities greater than zero are shown. Statistical significance was assessed by one-way ANOVA test. Asterisks **** indicate *P*-values of < 0.0001. Scale bar is 10 μm.

E BrdU-sensitized U-2-OS cells (Flp-In T-Rex; *CDKL5*–disrupted ($CDKL5^{\Delta/\Delta}$) or parental cells) stably expressing GFP-tagged ELOA wild-type (WT) or S^{311}A mutant were line-micro-irradiated and imaged after at the time points indicated.

Source data are available online for this figure.

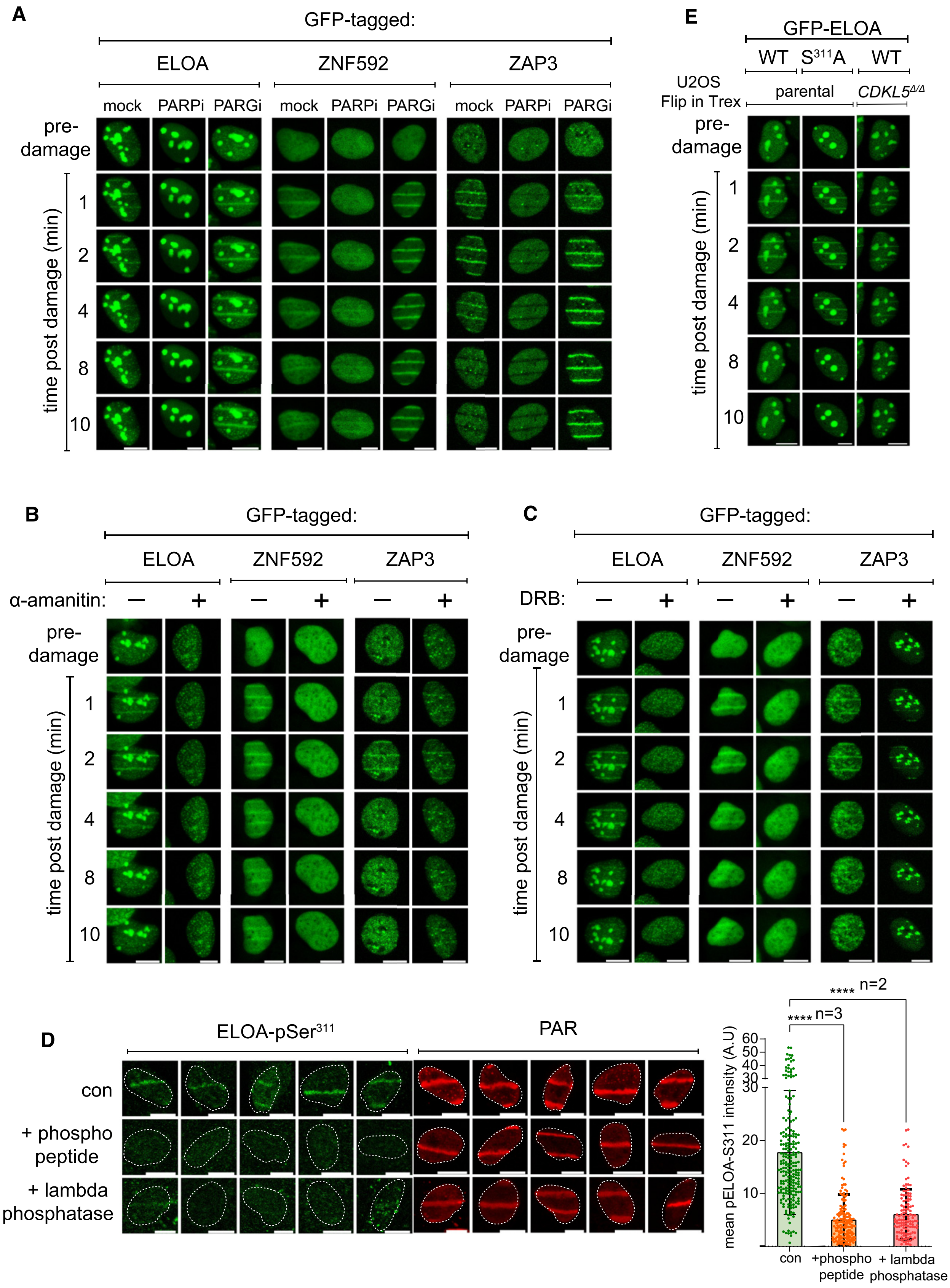

Figure EV4.

 © 2021 The Authors

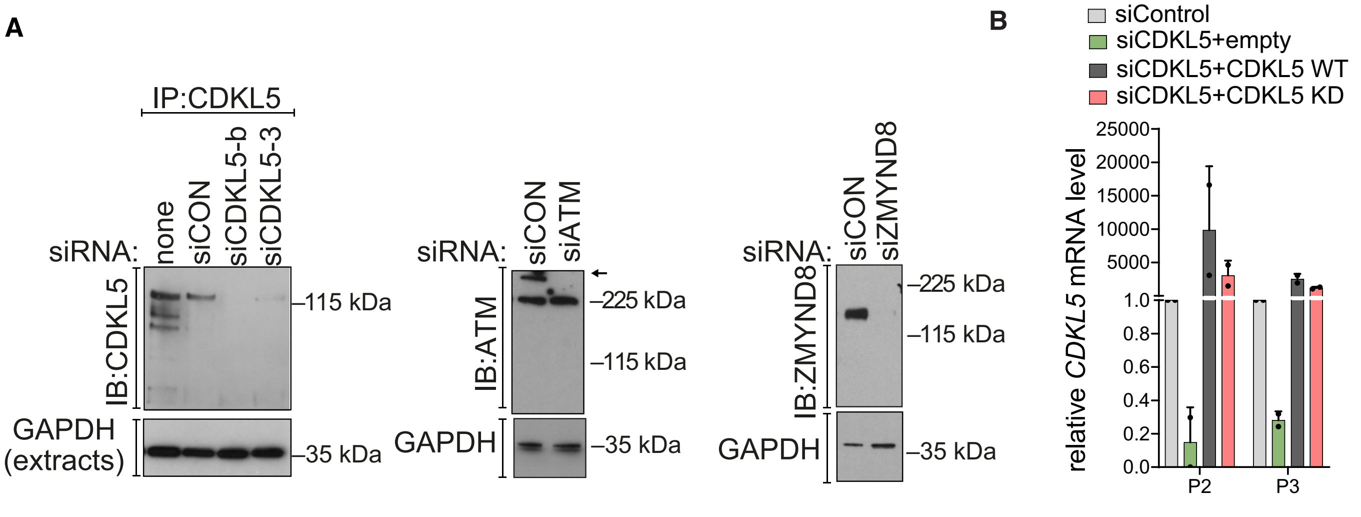

Figure EV5. siRNA-mediated knockdown of proteins in U-2-OS reporter cells.

A CDKL5, ATM and ZMYND8 were depleted in U-2-OS 263 IFII reporter cells using indicated siRNA. siCON—non-targeting control.

B qRT–PCR measurements in U2OS I-PpoI cells to validate the silencing efficiency of siCDKL5 and the subsequent rescue efficiency of ectopic expression of siRNA-resistant forms of CDKL5 wild-type (WT) and $K^{42}R$ kinase-dead mutant (KD). P2 and P3 primer pairs were used to analyse the changes in mRNA levels. The mean ± SD from two qPCR replicates of two independent experiments is shown.

Source data are available online for this figure.

© 2021 The Authors
