# Supplementary material for: CDKL5 kinase controls transcription‐coupled responses to DNA damage
Source: EMBO J. 2021 Oct 4;40(23):e108271. doi: 10.15252/embj.2021108271 (PMC8634139; doi:10.15252/embj.2021108271)
Supplement: Supplementary file 4 — Movie EV1 [file EMBJ-40-e108271-s013.zip › Movie EV1/Movie EV1 legend.docx]

**Movie EV1.** **Live–imaging of CDKL5 recruitment to sites of line–micro–irradiation.** U–2–OS Flp–In T–REx cells stably expressing GFP–NLS–CDKL5 were preincubated with BrdU overnight. An hour before micro-irradiation along a line in the nucleus using a 355 nm laser attached to a Leica TCS SP8X confocal microscope, cells were mock treated or treated with PARP inhibitor (olaparib, 5µM, 1h) or PARG inhibitor (PDD00017273, 0.3 µM, 1h). Cells were live imaged for the time indicated.
