# Supplementary material for: CDKL5 kinase controls transcription‐coupled responses to DNA damage
Source: EMBO J. 2021 Oct 4;40(23):e108271. doi: 10.15252/embj.2021108271 (PMC8634139; doi:10.15252/embj.2021108271)
Supplement: Supplementary file 5 — Movie EV2 [file EMBJ-40-e108271-s012.zip › Movie EV2/Movie EV2 legend.docx]

**Movie EV2. Live–imaging of CDKL5 recruitment to sites of spot–micro–irradiation**. U–2–OS Flp–In T–REx cells stably expressing GFP–NLS–CDKL5 were preincubated with BrdU overnight. Cells were mock treated or treated with PARP inhibitor (olaparib, 5 µM) or PARG inhibitor (PDD00017273, 0.3 µM) an hour before micro-irradiation in a spot-shaped sub-nuclear volume in the nucleus using a 405 nm laser attached to a Zeiss Axio Observer Z1 spinning disk confocal microscope. Cells were live imaged for the time indicated.
