# Supplementary material for: CDKL5 kinase controls transcription‐coupled responses to DNA damage
Source: EMBO J. 2021 Oct 4;40(23):e108271. doi: 10.15252/embj.2021108271 (PMC8634139; doi:10.15252/embj.2021108271)
Supplement: Supplementary file 7 — Source Data for Figure 1 [file EMBJ-40-e108271-s011.zip › Figure 1/Source data_Fig 1_B_C_E_G_Omero figure links.docx]

| **Figure 1B** | [OMERO.figure - Khanam et al. Fig 1B (dundee.ac.uk)](https://omero.lifesci.dundee.ac.uk/figure/file/381342/) |
| --- | --- |
| **Figure 1C** | [OMERO.figure - Khanam et al. Fig 1C (dundee.ac.uk)](https://omero.lifesci.dundee.ac.uk/figure/file/381338/) |
| **Figure 1E** | [OMERO.figure - Khanam et al. Fig 1E (dundee.ac.uk)](https://omero.lifesci.dundee.ac.uk/figure/file/381356/) |
| **Figure 1G** | [OMERO.figure - Khanam et al. Fig 1G (dundee.ac.uk)](https://omero.lifesci.dundee.ac.uk/figure/file/381335/) |
