# Supplementary material for: CDKL5 kinase controls transcription‐coupled responses to DNA damage
Source: EMBO J. 2021 Oct 4;40(23):e108271. doi: 10.15252/embj.2021108271 (PMC8634139; doi:10.15252/embj.2021108271)
Supplement: Supplementary file 8 — Source Data for Figure 2 [file EMBJ-40-e108271-s005.zip › Figure 2/Source data_Fig 2_B_D_Omero figure links.docx]

| **Figure 2B i** | [OMERO.figure - Khanam et al. Fig 2B part i (dundee.ac.uk)](https://omero.lifesci.dundee.ac.uk/figure/file/381346/) |
| --- | --- |
| **Figure 2B ii** | [OMERO.figure - Khanam et al. Fig 2B part ii (dundee.ac.uk)](https://omero.lifesci.dundee.ac.uk/figure/file/381348/) |
| **Figure 2D** | [OMERO.figure - Khanam et al. Fig 2D (dundee.ac.uk)](https://omero.lifesci.dundee.ac.uk/figure/file/381345/) |
