# Supplementary figures and images for: CDKL5 kinase controls transcription‐coupled responses to DNA damage
Source: EMBO J. 2021 Oct 4;40(23):e108271. doi: 10.15252/embj.2021108271 (PMC8634139; doi:10.15252/embj.2021108271)

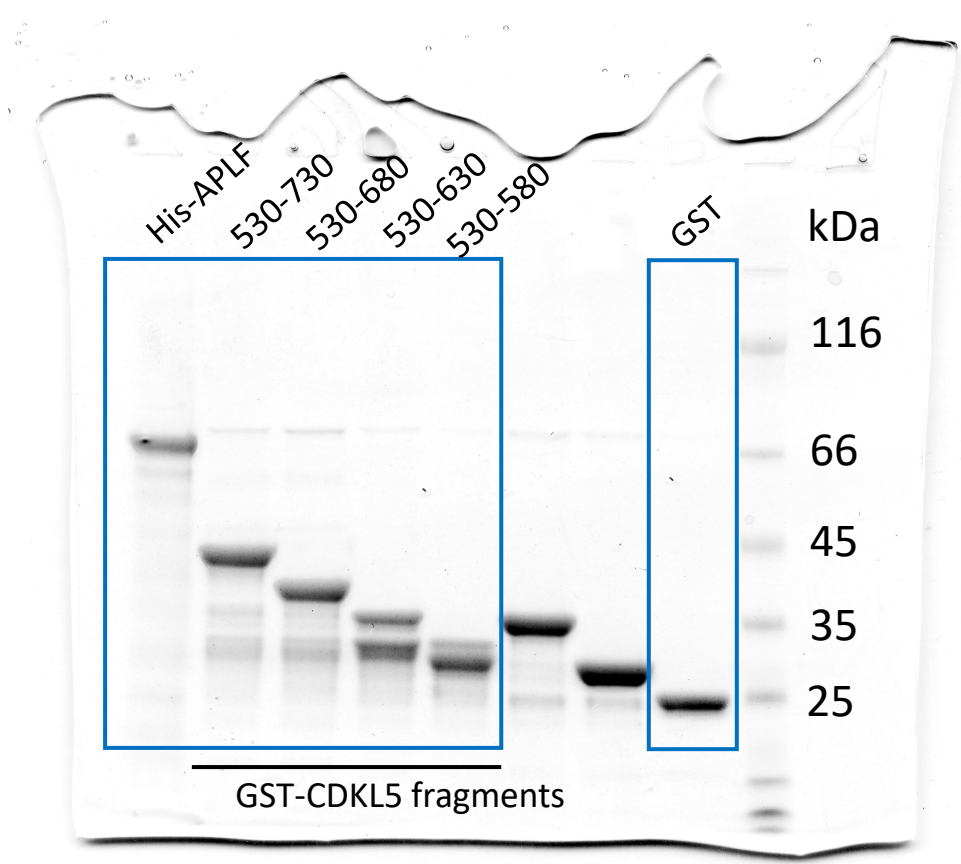

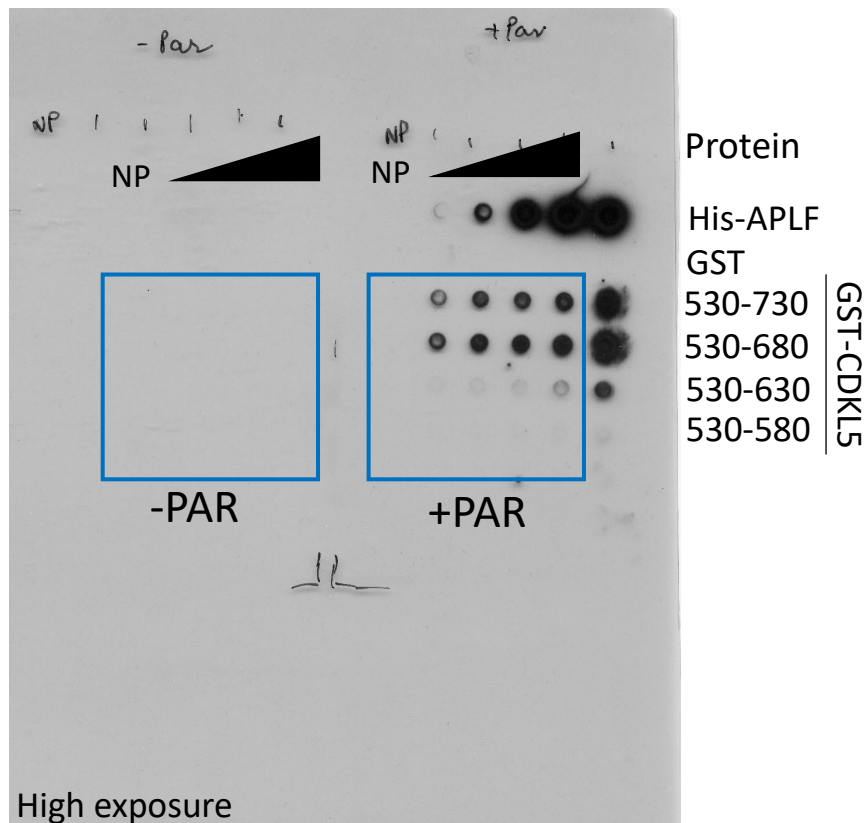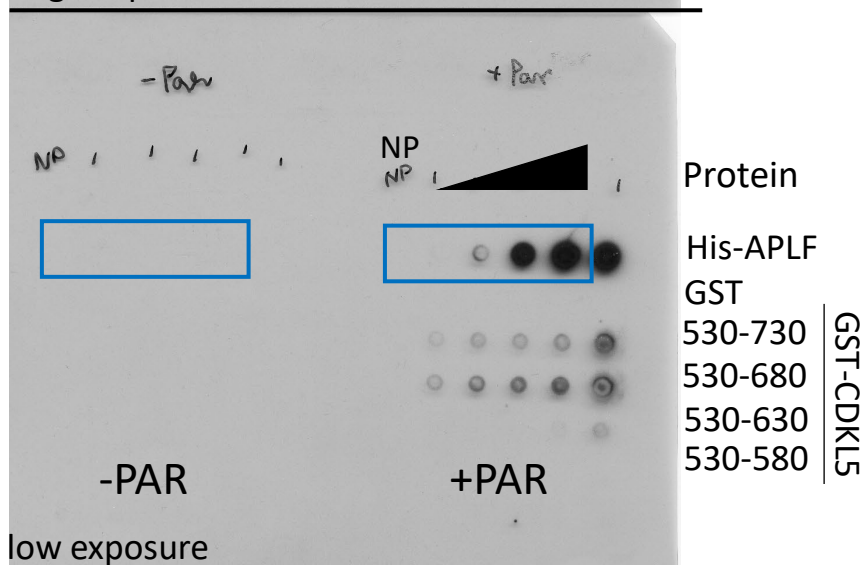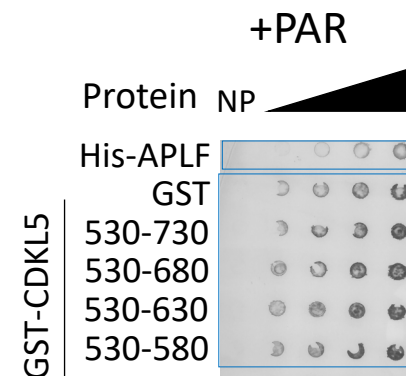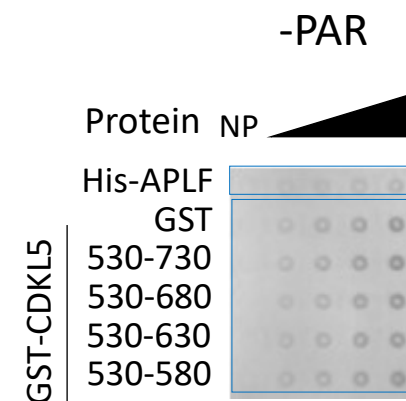

Figure: 2G

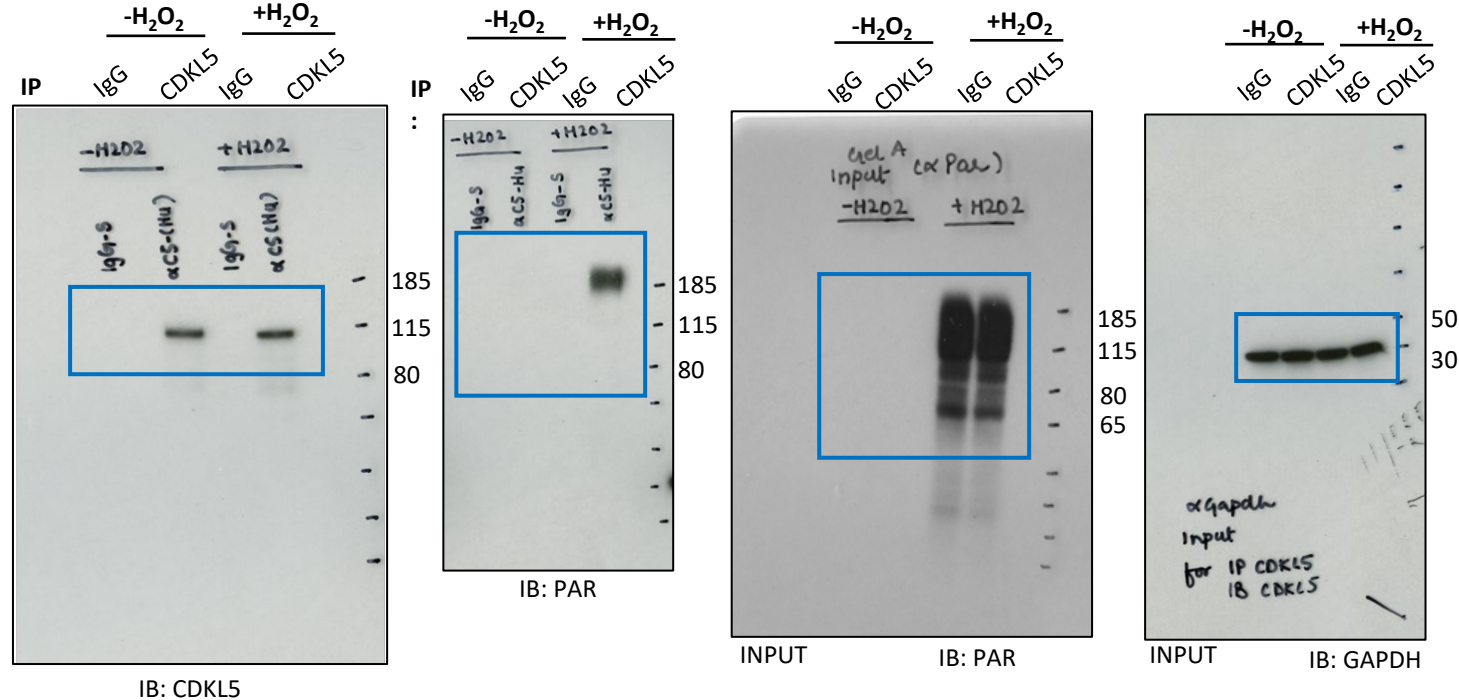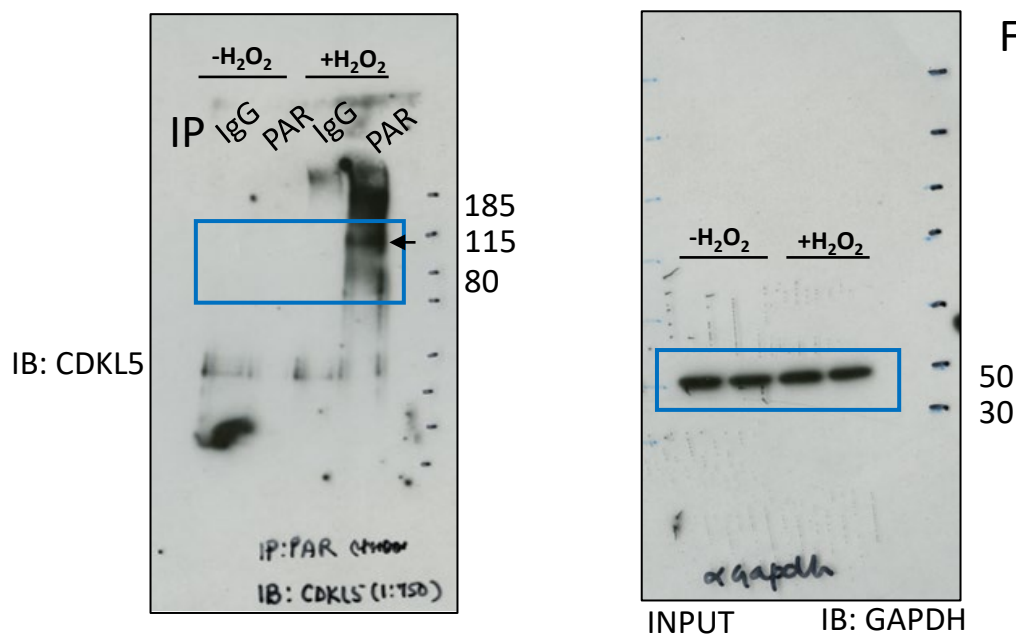

Figure: 2H

Supplement: Supplementary file 8 — Source Data for Figure 2 [file EMBJ-40-e108271-s005.zip › Figure 2/Source data_FIG 2E-H.pdf]

Figure 5

A.

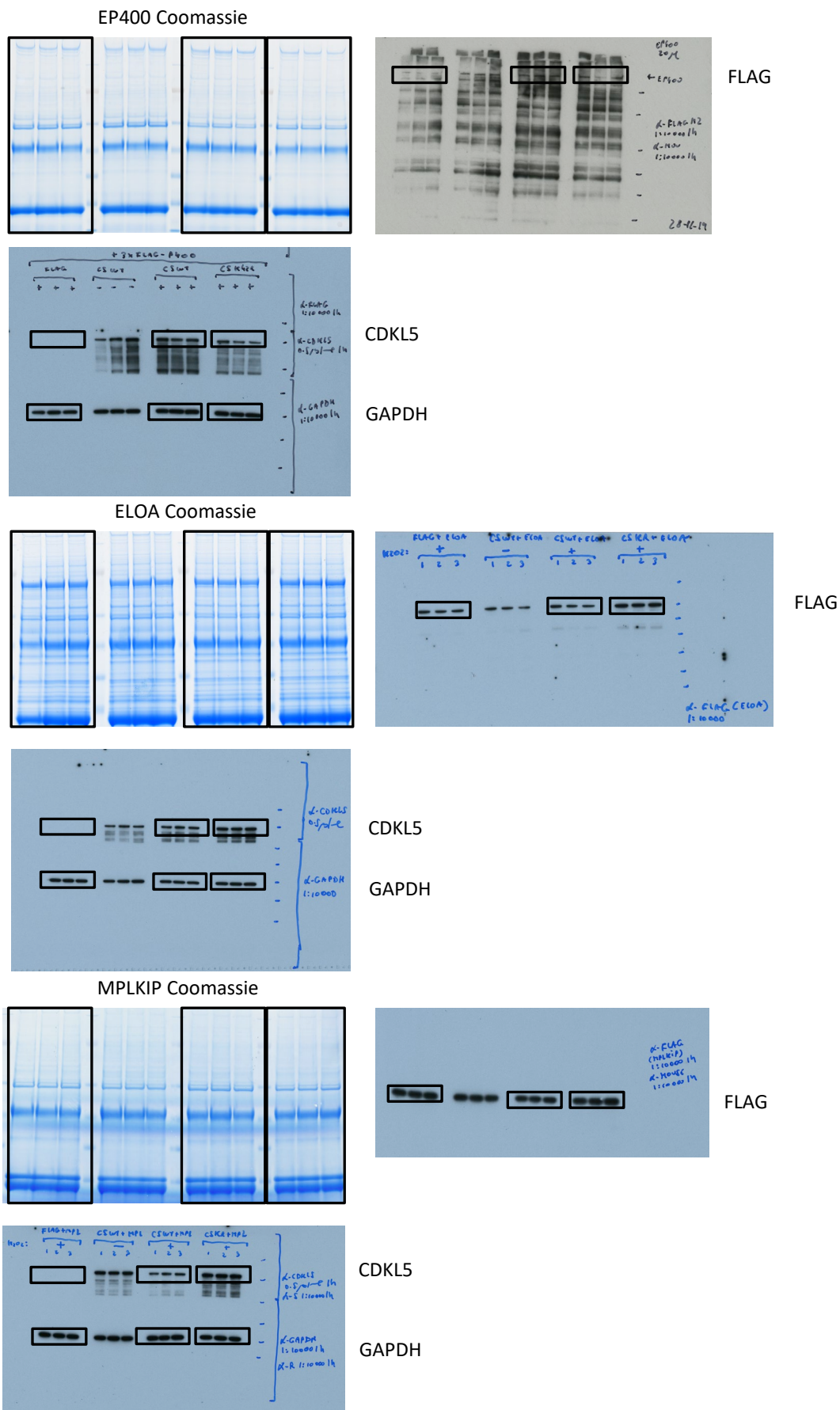

Figure 5

D.

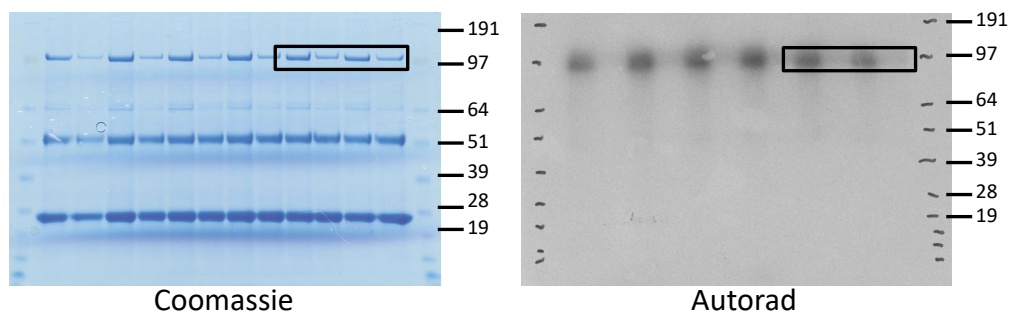

Coomassie

Autorad

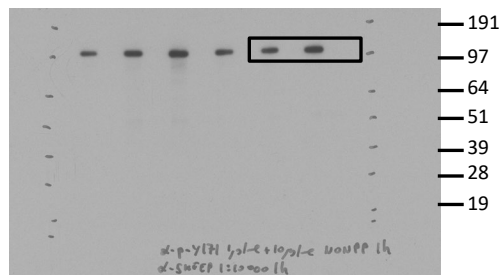

CDKL5-pTyr171

Supplement: Supplementary file 10 — Source Data for Figure 5 [file EMBJ-40-e108271-s001.zip › Figure 5/Source data Fig5A and D.pdf]

Figure 6

A.

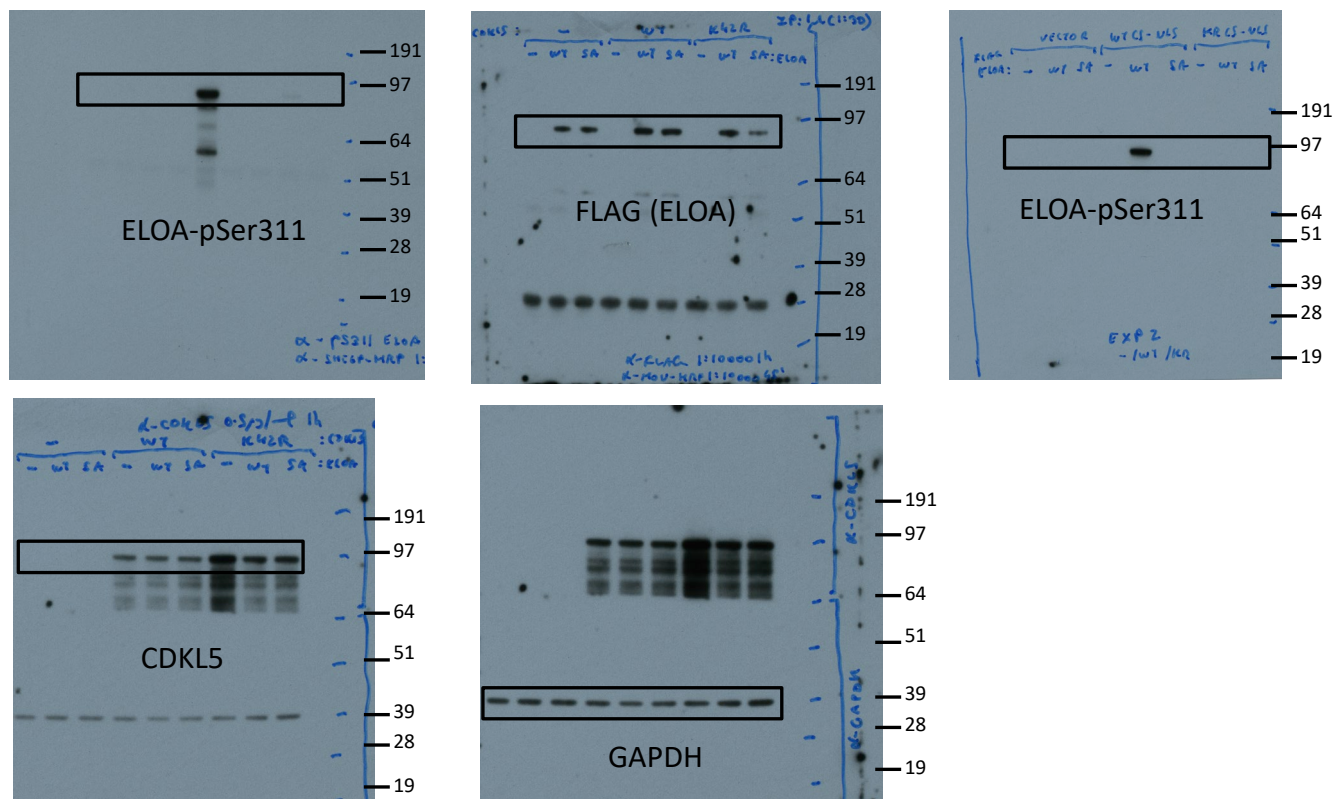

B.

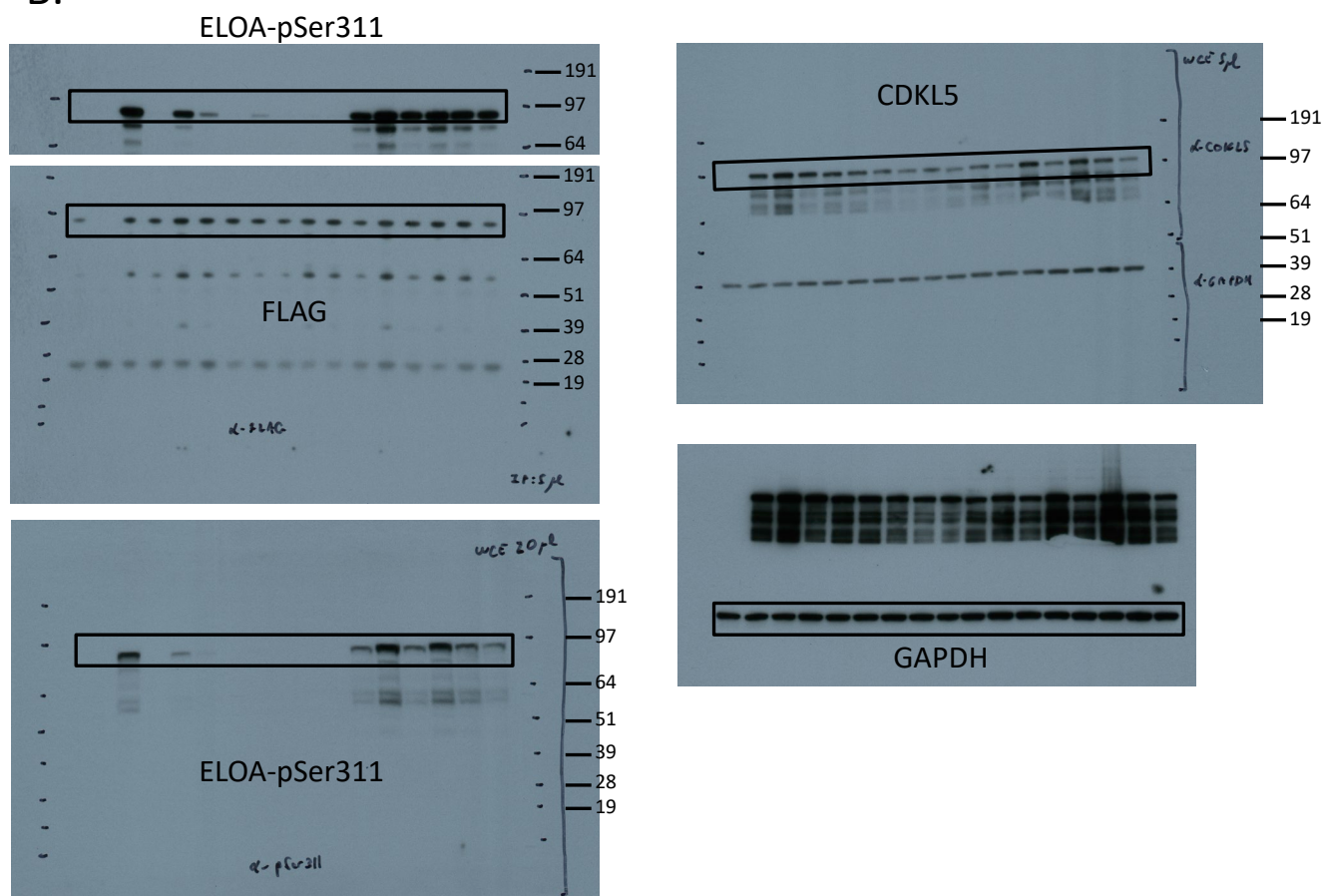

Supplement: Supplementary file 11 — Source Data for Figure 6 [file EMBJ-40-e108271-s007.zip › Figure 6/Source data Fig6A-B.pdf]
