# Supplementary material for: CDKL5 kinase controls transcription‐coupled responses to DNA damage
Source: EMBO J. 2021 Oct 4;40(23):e108271. doi: 10.15252/embj.2021108271 (PMC8634139; doi:10.15252/embj.2021108271)
Supplement: Supplementary file 9 — Source Data for Figure 3 [file EMBJ-40-e108271-s002.zip › Figure 3/Source data_Fig 3_A_B_C_D_E_F_Omero figure links.docx]

| **Figure 3A** | [OMERO.figure - Khanam et al. Fig 3A (dundee.ac.uk)](https://omero.lifesci.dundee.ac.uk/figure/file/381350/) |
| --- | --- |
| **Figure 3B** | [OMERO.figure - Khanam et al. Fig 3B (dundee.ac.uk)](https://omero.lifesci.dundee.ac.uk/figure/file/381341/) |
| **Figure 3C** | [OMERO.figure - Khanam et al. Fig 3C (dundee.ac.uk)](https://omero.lifesci.dundee.ac.uk/figure/file/381357/) |
| **Figure 3D** | [OMERO.figure - Khanam et al. Fig 3D (dundee.ac.uk)](https://omero.lifesci.dundee.ac.uk/figure/file/381347/) |
| **Figure 3E** | [OMERO.figure - Khanam et al. Fig 3E (dundee.ac.uk)](https://omero.lifesci.dundee.ac.uk/figure/file/381352/) |
| **Figure 3F** | [OMERO.figure - Khanam et al. Fig 3F (dundee.ac.uk)](https://omero.lifesci.dundee.ac.uk/figure/file/381349/) |
