# Supplementary material for: CDKL5 kinase controls transcription‐coupled responses to DNA damage
Source: EMBO J. 2021 Oct 4;40(23):e108271. doi: 10.15252/embj.2021108271 (PMC8634139; doi:10.15252/embj.2021108271)
Supplement: Supplementary file 11 — Source Data for Figure 6 [file EMBJ-40-e108271-s007.zip › Figure 6/Source data_Fig 6_C_D_E_Omero figure links.docx]

| **Figure 6C** | [OMERO.figure - Khanam et al. Fig 6C (dundee.ac.uk)](https://omero.lifesci.dundee.ac.uk/figure/file/381358/) |
| --- | --- |
| **Figure 6D** | [OMERO.figure - Khanam et al. Fig 6D (dundee.ac.uk)](https://omero.lifesci.dundee.ac.uk/figure/file/381343/) |
| **Figure 6E** | [OMERO.figure - Khanam et al. Fig 6E (dundee.ac.uk)](https://omero.lifesci.dundee.ac.uk/figure/file/381344/) |
