# Supplementary material for: CDKL5 kinase controls transcription‐coupled responses to DNA damage
Source: EMBO J. 2021 Oct 4;40(23):e108271. doi: 10.15252/embj.2021108271 (PMC8634139; doi:10.15252/embj.2021108271)
Supplement: Supplementary file 12 — Source Data for Figure 7 [file EMBJ-40-e108271-s014.zip › Figure 7/Source data_Fig 7_B_C_Omero figure links.docx]

| **Figure 7B** | [OMERO.figure - Khanam et al. Fig 7B (dundee.ac.uk)](https://omero.lifesci.dundee.ac.uk/figure/file/381351/) |
| --- | --- |
| **Figure 7C** | [OMERO.figure - Khanam et al. Fig 7C (dundee.ac.uk)](https://omero.lifesci.dundee.ac.uk/figure/file/381340/) |
