# Supplementary material for: CDKL5 kinase controls transcription‐coupled responses to DNA damage
Source: EMBO J. 2021 Oct 4;40(23):e108271. doi: 10.15252/embj.2021108271 (PMC8634139; doi:10.15252/embj.2021108271)
Supplement: Supplementary file 14 [file EMBJ-40-e108271-s004.pdf]

CDKL5 kinase controls transcription-coupled responses to DNA damage

John Rouse, Taran Khanam, Ivan Muñoz, Florian Weiland, Thomas Carroll, Barbara Borsos, Vasiliki Pantazi, Meghan Slean, Miroslav Novak, Rachel Toth, Paul Appleton, Tibor Pankotai, Houjiang Zhou, and Michael Morgan
DOI: 10.15252/embj.2021108271

Corresponding author(s): John Rouse (j.rouse@dundee.ac.uk)

Review Timeline:

Submission Date:	16th Mar 21
Editorial Pre-consultation:	12th Apr 21
Authors' Preliminary Response:	16th April 21
Editorial Decision:	19th Apr 21
Revision Received:	26th Jul 21
Editorial Decision:	27th Aug 21
Revision Received:	8th Sep 21
Accepted:	9th Sep 21

Editor: Hartmut Vodermaier

Transaction Report:

(Note: With the exception of the correction of typographical or spelling errors that could be a source of ambiguity, letters and reports are not edited. Depending on transfer agreements, referee reports obtained elsewhere may or may not be included in this compilation. Referee reports are anonymous unless the Referee chooses to sign their reports.)

Editorial Pre-consultation: 12th Apr 2021

Thank you again for submitting your manuscript EMBOJ-2021-108271, "CDKL5 kinase recognizes transcription-associated DNA damage and controls transcription at DNA breaks." We have now received a complete set of reviews from three referees, which you will find enclosed below for your information. As you will see, the referees generally appreciate the study's topic, quality, and findings, but also raise a number of important queries. While some of them may go beyond the scope of the present investigation and/or may not be addressable without substantive further efforts, other points appear to be well-taken and possibly straightforward to tackle. In this situation, I would very much appreciate hearing from you how the different points might be answered and which revision experiments you could envision to address particularly salient issues. Therefore, please carefully consider the attached reports and send back a brief point-by-point response outlining how the referees' comments might be addressed/clarified. I could then take these tentative responses (parts of which we may choose to share and discuss with referees) into account when making our final decision regarding eventual further consideration of this work as an EMBO Journal research or resource article. It would be great if you could get back to me with such a response by early next week.

REFEREE REPORTS

Referee #1 (Report for Author)

This is a really nice body of work that on the whole supports the authors statements linking CDKL5 (activity) with transcriptional regulation at DNA breaks. However, I have concerns that some of the data are over-interpreted and the authors are drawing conclusions that really cannot be made based on the evidence presented. Although I have experience in DNA damage, my expertise is focussed on the area of phosphorylation-mediated signalling and proteomics, therefore I have focused my review primarily on these aspects of the manuscript.
General comments:
A key question is: how do they know that any of the differentially regulated phosphorylation sites are genuine CDKL5 targets, as opposed to indirect substrates being modulated by a CDKL5-reguated kinase? While the HEK293 validation of potential CDKL5 substrates does not actually rule out these proteins being phosphorylated indirectly by a kinase that itself is regulated by CDKL5, the in vitro assays with peptide substrates do support this hypothesis, at least for Ser729 on EP400 and Ser311 on ELOA. Given the possibility for indirect regulation in both the HTP screen and the 293 cell experiments, the authors need to tone down their language to reflect this possibility.

How do the authors explain identification of cytoplasmic substrates given the time course of these studies and the targeted nuclear localisation CDKL5?

The authors describe ELOA pSer311 as a potential characteristic biomarker of CDD, based on decreased observation in cell lines containing CDD-associated CDKL5 mutations. I think this is oversimplifying the situation and assumes that this residue is not modified by any other kinase, and that there is no effect of a phosphatase that would mask this effect. In the absence of validation with clinical samples, this statement should be removed.

The final series of experiments demonstrate that knock-down of CDKL5 weakens the silencing of transcriptional near DNA breaks. However, there is no evidence currently that this is actually dependent on CDKL5 kinase activity. While they do not state this to be the case, evaluation of KD versus WT CDKL5 in this regard would bring the manuscript together based on their original hypothesis.

Technical concerns:

1. The total number of phosphopeptide identified are details, but there is no mention of how many phosphopeptides (or sites) were significantly regulated as a function of expression of inactive CDKL5. What proportion of these were nuclear?
2. There is no mention in the methods section of how (or if!) the phosphoproteomics data were filtered for site localisation confidence. This is particularly relevant as they are applying a 5% FDR, rather than the more typical 1% FDR. 223 phosphopeptides with p-value<0.01 (2079 at p<0.05) were in this list and a cursory glance through these would suggest that a relatively small number are above the typically applied 0.75 PTM-score threshold (assuming this is PTM-score that they are using), let alone the 0.994 value that equates to a 1% FLR as suggested in Ferries et al JPR (2017). I'm not sure what justification the authors have for selecting a p-site probability of 0.6 as they have stated in the legend to Fig 4. They need to be explicit about which particular score they apply, and justification for using a 0.6 cut-off.
3. Where site localisation is ambiguous (i.e. below 0.75 or 0.994 dependent on what cut-off you select), this needs to be explicitly stated in any downstream analysis, and the data treated separately. Looking at the Supp. Tables, they do not appear to have filtered based on site localisation at all, but overall this is confusing - for example, GLVRPGS(0.5)S(0.5)R is identified from P49750 where Ser at positions 7 or 8 are equally as likely to be phosphorylated (hence both have a localisation score of 0.5), yet the phosphorylated residue that appears to have been considered is Ser7. There are numerous similar examples - I am thus concerned based on this that the authors may be cherry-picking their data, as they haven't provided sufficient explanation of what they have actually done.
4. As a general point, it is unclear how they are filtering the data presented in Fig. 4C. It is more important to filter by statistical probability of a site being regulated, rather than the fold change, which will also be influenced potentially by the activity of other kinases/phosphatases. I am not a fan of fold-change filtering is appropriate statistical cut-offs are applied.
5. Can you please provide details on imputation of null values. The volcano plot would suggest that this was done, but this is not explained.
6. What proportion of all phosphorylation sites, versus the statistically significantly regulated phosphosites match the consensus (considering a suitable oFLR of >0.75). To increase confidence of this being a CDKL5 consensus motif, you should show enrichment of those sites that are regulated against a background of all identified phosphosites.
7. Please provide some more details (supp. Table) on GO term enrichment (that will likely need to be re-done using the newly filtered dataset) - typically you would require 3 proteins per group.

Referee #2 (Report for Author)

In this manuscript, Khanam et al. present evidence that CDKL5 is recruited to sites of DNA damage in actively transcribed regions of the nucleus. By performing a quantitative phospho-proteomic screen for nuclear CDKL5, they identify several transcriptional regulators as substrates including Elongin A, phosphorylated on a specific CDKL5 consensus motif. Recruitment of CDKL5 and Elongin A to damaged DNA, and subsequent phosphorylation of Elongin A, requires both active transcription and the synthesis of poly-ADP ribose, to which CDKL5 can bind. They also present evidence that CDKL5 facilitates transcriptional silencing of genes that lie near DNA breaks. The data are interesting, are presented clearly and the manuscript is well written. However, my main concern about the current manuscript is that the physiologic relevance of phosphorylation of nuclear targets of CDKL5 is unclear.
I think therefore the conceptual advance achieved by the findings is not sufficient to support publication of this paper in EMBO Journal.

Major points:
(1) Elongin A performs dual functions as an RNA polymerase II (pol II) elongation factor and as the substrate recognition subunit of a Cullin-RING E3 ubiquitin ligase that targets pol II stalled at sites of DNA damage. It is already known that Elongin A and Cul5 are rapidly recruited in cells to regions of localized DNA damage, and that assembly of Elongin A and Cul5 is triggered following DNA damage as well as by treatment of cells with pol II inhibitors; alpha-amanitin and DRB (Weems JC et al. JBC 2015). In addition, Elongin A and Cul5 associate in cells with the Cockayne syndrome B (CSB) protein and this interaction is also induced by DNA damage, alpha-amanitin and DRB (Weems JC et al. JBC 2017).

Does the phosphorylation of Elongin A by CDKL5 affect either of the above processes? This question needs to be answered by additional studies.

Minor points:
(1) Since the amino acid sequences of Elongin A are highly conserved between human, rat (773 aa protein) and mouse (773 aa protein), I believe human Elongin A is composed of 772 amino acids (A0A024RAC6). Therefore, phosphorylation site of human Elongin A will be not Ser311, but Ser285. Ser311 in the sentences and figures may be better to be changed to Ser285.
(2) In the bottom line of page 11, Figs. 4A-C need to be changed to Figs. EV4A-C.
(3) In several references, the volume and page numbers are missing. Those should be corrected.

Referee #3 (Report for Author)

In this manuscript, Khanam and colleagues reports a novel function of the kinase CDKL5, frequently mutated in the rare neurodevelopmental condition called CDD and that also causes childhood epilepsy. The rationale of this story is to decipher the poorly understood functions of CDKL5 in the nucleus. The authors found that CDKL5 plays roles in the DNA damage response. Accordingly, CDK5L is recruited to DNA damage lesions; a localization that is dependent on transcription and PAR. In order to assess the functions of CDKL5, the authors first set up a phospho-proteomic screen to identify the phospho-targets of CDKL5. They identify many transcriptional regulators and particularly focused their attention on ELOA, which had previously been implicated in the DNA damage response. This protein was reported to display dual functions by acting as a transcriptional elongation factor in unstressed condition and as a component of the E3 ubiquitin ligase complex, known to ubiquitylate and degrade RNA pol II, upon genotoxic stress conditions. The authors confirm that CDKL5 phosphorylates synthetic ELOA peptides at Ser311 in vitro and also endogenous ELOA in vivo. They also nicely link the inability of CDKL5 pathogenic CDD-associated mutations to phosphorylate ELOA. Similar to CDKL5, ELOA is recruited to DNA lesion in a PAR- and transcription-dependent manner. Phosphorylation of ELOA Ser311 at sites of DNA damage requires CDKL5. Lastly, the authors show that CDKL5 mediates transcriptional repression in the context of DNA double-strand breaks.

Main comments

This novel implication of CDKL5 in the DNA damage response is interesting and clearly deserves to be reported, especially since CDKL5 is associated with the CDD neurodevelopmental disorder, which expands the list of pathogenic variants of DNA repair proteins linked to neurological diseases. The authors start by the initial characterization of the localization of CDKL5 to DNA damage lesions, which is followed by its substrate identification, and lastly function of CDKL5. The manuscript will be of great interest for the community but could be improved by answering the following major points.

1) Although the data clearly show that DRB/a-amanitin pre-treatment abrogates recruitment of CDKL5, additional experiments would be required to fully confirm that CDKL5 is recruited at transcription-associated DSB. We suggest to revise the title and modify sentences throughout the manuscript to be less assertive on this point.
2) It is unclear whether CDKL5-mediated phosphorylation of ELOA is DNA damage-dependent. Additionally, it would be useful to know whether unphosphorylated ELOA is recruited in a CDK5L-dependent manner at DNA lesions, or is it only the Ser311 phosphorylation at damage sites that is CDKL5 dependent?
3) The authors present functional data on CDKL5 and show that it is implicated in the transcriptional repression that occurs post-DSB induction. Yet this part of the manuscript is somehow disconnected to the previous part where the relationship between CDKL5 and ELOA is made. Is transcriptional repression dependent on ELOA and/or on the Ser311 phosphorylated form of ELOA?

4) While CDKL5 is recruited at sites of laser microirradiation, it would be useful if the authors could show its recruitment at FokI-induced DNA damage. Regarding CDKL5 recruitment at DNA damage, the data presented Fig. 1G, are puzzling, since GFP-NLS alone behaves as GFP-NLS-CDKL5 (increases post H202). It looks like there is a mistake in the annotation (GFP-CDKL5: 5 cells shown without H202, 4 cells only with H202 and 6 with PARPi). If not, it seems difficult from the data presented here to conclude that CDKL5-GFP is recruited at DNA damage. Finally, did the authors assessed whether endogenous CDKL5 also localizes to laser micro-irradiation or H2O2-induced damage?

Minor points

A) Different isoforms of CDK5L have been reported (PMID: 27315173). Could the authors specify in material and methods which isoform was used in this study?
B) Could the authors provide a reference when citing APLF as a positive control for PAR binding?
C) The authors also need to describe in the Materials and methods the experimental procedure for the fractionation experiment
D) When the authors mention the localization of ELOA to DNA damage (p11 last line), the wrong figures are cited. It should be Fig 6C-E instead of Fig4A-C.
E) Fig. 4B the x axis deserves better labelling. Is it the log2 fold change of WT/KD?

Please find attached our preliminary responses to the Referees' comments on our CDKL5 study.

As you'll see, the responses to Referees 2 and 3 are quite brief. The comments from Referee 1 made us realise that the description of how we analysed the mass spec data must have lacked clarity. To address this issue, we have written a pretty detailed response. There was a typo in the text relevant to our data analysis, and we've set this straight. There was also a simple mistake in Fig 1G relevant to the last comment of Referee 3. A corrected version of Fig. 1 is attached.

I hope the responses and the list of extra experiments we are doing to address the comments are satisfactory. Please let me know if you need any more information.

Referee 1
We are grateful to referee 1 for their insightful comments, especially on the mass spectrometric data analysis methodologies. It's clear now that the text could be improved in terms of clarity, especially regarding how we analysed the MS data. We will re-work the text to address this issue.

1. ***The total number of phosphopeptide identified are details, but there is no mention of how many phosphopeptides (or sites) were significantly regulated as a function of expression of inactive CDKL5. What proportion of these were nuclear?***
Based on analyses described in point 2 below, we identified 37 peptides (31 unique peptide sequences, 24 unique proteins) which were higher in abundance in the CDKL5-NLS-WT samples compared with the CDKL5-NLS-KD samples. 14 out of 24 proteins are known to be nuclear in localization (GO: 0005634 (nucleus and nucleus-offspring GO terms).

2. ***There is no mention in the methods section of how (or if!) the phosphoproteomics data were filtered for site localisation confidence. This is particularly relevant as they are applying a 5% FDR, rather than the more typical 1% FDR. 223 phosphopeptides with p-value<0.01 (2079 at p<0.05) were in this list and a cursory glance through these would suggest that a relatively small number are above the typically applied 0.75 PTM-score threshold (assuming this is PTM-score that they are using), let alone the 0.994 value that equates to a 1% FLR as suggested in Ferries et al JPR (2017). I'm not sure what justification the authors have for selecting a p-site probability of 0.6 as they have stated in the legend to Fig 4. They need to be explicit about which particular score they apply, and justification for using a 0.6 cut-off.***
We apologize that there is a mistake in the manuscript which may have caused confusion. The line on the last line of page 9 "…CDKL5–NLS–WT cells compared with CDKL5–NLS–KD cells (>1.5–fold, p<0.05)" would, as the Referee mentioned, have resulted in the 2079 phospho-peptides. However, this line should have read "…CDKL5–NLS–WT cells compared with CDKL5–NLS–KD cells (>1.5–fold, p < 0.0005)". In essence, we analysed the volcano plot shown in Figure 4B, which clearly showed 37 phospho-peptides (31 unique sequences) that were higher in abundance in the CDKL5-WT samples compared with the KD samples; this group clustered away from the bulk of phospho-peptides, and all the phospho–peptides within this cluster had p < 0.0005. These 37 phospho-peptides were assigned as the peptides of interest. Within this group of 31 unique sequences, an RPX[S/T] sequence motif occurs 25 times. In 21 out of these 25 cases, Andromeda assigned a phosphorylation site to the RPXp[S/T] motif. When a 75% filter is applied, the assignment of 18 of these peptides remains (removing POM121C, NCOA5 and ZAP3). When a 100% filter is applied, the assignment of 9 of these peptides remains. The cut-off of 0.6 was chosen arbitrarily for the sake of simplifying the table in Figure 4C. In the revised manuscript we will add an extra column in Fig. 4C depicting the actual site probabilities. We will also try to draft a revised table where we add a line to indicate where the 1% FDR cut-off lies and/or indicate 75% and 100% site localization cut-offs. We will also spell out these considerations more explicitly in the legend to Figure 4 and in the Methods section.

We should point out some unpublished data relevant to our analyses. In our 2018 paper (PMID: 30266825), we noticed that the phosphorylated residues in the cytosolic substrates we validated all occurred in the motif R-P-X-S*-A. Using synthetic peptides based on the CDKL5 phosphorylation site in MAP1S, we narrowed down the CDKL5 specificity determinant (consensus) to R-P-X-[S/T]*-[A/G/P] – but of course this came from *in vitro* data, and we only tried a handful of residues in the +1 position relative to the phospho–acceptor. SInce those data were published, we substituted the A at +1 in a synthetic peptide from MAP1S to every other amino acid, which refined to CDKL5 consensus to R-P-X-[S/T]*-[A/G/P/S]. But we had no idea if this was relevant to CDKL5 in cells. In the current study, all the RPX[S/T] motifs mentioned above had A, G, P or S in the +1 position which seemed to

validate the *in vitro* peptide data. We're thinking we should include the data leading to the refined consensus in the paper.

3. ***Where site localisation is ambiguous (i.e. below 0.75 or 0.994 dependent on what cut-off you select), this needs to be explicitly stated in any downstream analysis, and the data treated separately. Looking at the Supp. Tables, they do not appear to have filtered based on site localisation at all, but overall this is confusing - for example, GLVRPGS(0.5)S(0.5)R is identified from P49750 where Ser at positions 7 or 8 are equally as likely to be phosphorylated (hence both have a localisation score of 0.5), yet the phosphorylated residue that appears to have been considered is Ser7. There are numerous similar examples - I am thus concerned based on this that the authors may be cherry-picking their data, as they haven't provided sufficient explanation of what they have actually done.***
In the case of P49750 – called ZAP3/YLPM1, several closely-related peptides were present in the phospho-proteomics dataset in Table EV1, from different RP chromatography fractions: GLVRPGS(0.5)S(0.5)R (fold-change 13.08, p = 4.47E-10); GLVRPGS(0.101)S(0.899)R (fold-change 6.23, p = 8.96E-08); DRGLVRPGS(0.877)S(0.123)R (fold-change 4.42, p = 2.64E-09). The numbers in brackets in the peptide sequence are the phospho-site probabilities assigned by Andromeda. Based on these numbers, the referee rightly points out that it's not possible to know if it's the first or second serine in this motif that's phosphorylated by CDKL5 – it could be just one, or both. We agree, and that's why we show ZAP3 twice in the table in Figure 4C, each with one of the two serines highlighted in red. We're disappointed the referee thinks we're cherry picking especially as we were careful to highlight the second serine. To help clarify this point in the revised paper, we will spell out these considerations more explicitly in the legend to Figure 4 and in the Methods section. We will include a column in Figure 4C showing the localisation site probabilities. We will also revise Table EV1 and apply a 75% phosphosite localization filter to the column labelled "Phosphorylation site part of an RPX[S/T] motif".

4. ***As a general point, it is unclear how they are filtering the data presented in Fig. 4C. It is more important to filter by statistical probability of a site being regulated, rather than the fold change, which will also be influenced potentially by the activity of other kinases/phosphatases. I am not a fan of fold-change filtering is appropriate statistical cut-offs are applied.***
After much discussion we opted not to present our data filtered based on a pre-defined statistical significance cut-off. We were influenced, for example, by recent considerations on the potential pitfalls associated with this approach (see Figure 4 and discussion in: *Blume JD, Greevy RA, Welty VF, Smith JR & Dupont WD (2019) An Introduction to Second-Generation p-Values. Am Stat* ***73****: 157–167*). Therefore, we simply analysed the volcano plot as described in the response to point 2 above and focussed on the 37-member phospho-peptide group that clustered away from the bulk.
>The arbitrary cut-off of fold-change > 1.5 in Figure 4C was chosen for the sake of simplifying the table.
> We agree that the sites identified in this study could be targets of other kinases/phosphatases. But we argue that we're looking specifically a CDKL5-dependent phosphorylation of individual sites in our study.
>Applying a 1% FDR cut-off to the data as the Referee suggested, leaves 18 unique peptides of interest. The approach we took showed 31 unique peptides of interest. One of these (TTDN1) would have been excluded by the 1% FDR cut-off, and yet this was one of the proteins validated as being phosphorylated in a CDKL5-dependent manner in XIC experiments. Nonetheless we will look at ways to indicate where the 1% FDR cut-off lies in Figure 4C.

5. ***Can you please provide details on imputation of null values. The volcano plot would suggest that this was done, but this is not explained.***
Out of 90,868 peptides detected in our phospho–proteomic dataset (after excluding peptides with >5% sequence identification FDR, removal of potential contaminants as flagged by MaxQuant, parent ion fraction < 0.60 and reverse database hits), 100 peptides in the KD group and 64 peptides in the WT group had one or more missing datapoints which were assigned "NA". Peptides with only a single datapoint within KD (8 peptides) and/or WT groups (6 peptides) were removed prior to statistical analysis.

6. ***What proportion of all phosphorylation sites, versus the statistically significantly regulated phosphosites match the consensus (considering a suitable oFLR of >0.75). To increase confidence of this being a CDKL5 consensus motif, you should show enrichment of those sites that are regulated against a background of all identified phosphosites.***
Within the group of 31 unique peptides of interest, there are 21 unique phospho-peptides with putative phosphorylation sites in form of an RPXp[S/T] motif (as assigned by Andromeda). Applying a 75% filter, leaves 18 phospho-peptides (after removal of POM121C (RPLS(0.645)S(0.355)), NCOA5 (RPVS(0.687)S(0.174)) and ZAP3 (GLVRPGS(0.5)S(0.5))). Applying a filter setting of 100% leaves 9 peptides with an RPXp[S/T] motif. All of these RPXp[S/T] motifs have A, G, P or S at the +1 position.
>Within the complete dataset (46,258 unique phospho-peptides) 492 peptides exhibit an RPXp[S/T] motif (regardless of site localisation probability). When only taking the 100% filtered RPXp[S/T] peptides within our peptides of interest group into account, this is a 27-fold enrichment of this motif as compared to the background ($p < 0.00001$ (Fisher's exact test)).

7. ***Please provide some more details (supp. Table) on GO term enrichment (that will likely need to be re-done using the newly filtered dataset) - typically you would require 3 proteins per group.***
As mentioned above in the last line on page 9 "…CDKL5NLS–WT cells compared with CDKL5NLS–KD cells (>1.5–fold, $p<0.05$)" should read "…CDKL5NLS–WT cells compared with CDKL5NLS–KD cells ($p < 0.0005$)". For the gene ontology analyses, we extracted all GO terms attached to the 25 unique proteins identified as higher or lower abundant in WT by our volcano plot analysis (foreground) and tested for GO term enrichment against the remaining 5960 identified proteins (background) using a Fisher's exact test. As we only have 25 unique proteins as foreground, we chose a cut-off of at least two proteins per significant GO term. Most of the significant GO terms are closely related (Figure 4E). We will revise the Methods part to clarify this and include the number of protein hits within each GO term category (Figure 4E).

8. ***The final series of experiments demonstrate that knock-down of CDKL5 weakens the silencing of transcriptional near DNA breaks. However, there is no evidence currently that this is actually dependent on CDKL5 kinase activity. While they do not state this to be the case, evaluation of KD versus WT CDKL5 in this regard would bring the manuscript together based on their original hypothesis.***
> We are attempting to carry out rescue-type experiments to test if the kinase activity of CDKL5 is required for I-PpoI-induced gene silencing. This has proven to be technically tricky but we are working on it.

Referee 2
We are grateful to referee 2 for their insightful comments.
Major points:
(1) ***Elongin A performs dual functions as an RNA polymerase II (pol II) elongation factor and as the substrate recognition subunit of a Cullin-RING E3 ubiquitin ligase that targets pol II stalled at sites of DNA damage. It is already known that Elongin A and Cul5 are rapidly recruited in cells to regions of localized DNA damage, and that assembly of Elongin A and Cul5 is triggered following DNA damage as well as by treatment of cells with pol II inhibitors; alpha-amanitin and DRB (Weems JC et al. JBC 2015). In addition, Elongin A and Cul5 associate in cells with the Cockayne syndrome B (CSB) protein and this interaction is also induced by DNA damage, alpha-amanitin and DRB (Weems JC et al. JBC 2017).***
Does the phosphorylation of Elongin A by CDKL5 affect either of the above processes? This question needs to be answered by additional studies.
>We already found that ELOA is recruited to DNA damage sites with the same apparent efficiency in CDKL5 KO cells as in parental cells, implying that phosphorylation of Ser311 is not important for recruitment. We will include these data in the revised paper, and we will follow this up by analysing recruitment of an ELOA Ser311A mutant.
>We have not tested the impact of ELOA Ser311 phosphorylation on interaction with CUL5 or CSB, but we will now test this in CDKL5 KO cells and parental cells before and after inducing DNA damage; we will compare WT ELOA with a Ser311A mutant.

Minor points:
(1) Since the amino acid sequences of Elongin A are highly conserved between human, rat (773 aa protein) and mouse (773 aa protein), I believe human Elongin A is composed of 772 amino acids (A0A024RAC6). Therefore, phosphorylation site of human Elongin A will be not Ser311, but Ser285. Ser311 in the sentences and figures may be better to be changed to Ser285.
> A0A024RAC6 appears to be an unreviewed Uniprot entry, corresponding to a short ELOA isoform (772 amino acids). In our study, we use the ELOA accession number Q14241.2 which is a reviewed entry in Uniprot. We therefore propose to keep the pSer311 annotation in the text, being careful to mention which isoform and accession number we're referring to.

Referee 3
We are grateful to referee 2 for their insightful comments.
Main comments
1) ***Although the data clearly show that DRB/a-amanitin pre-treatment abrogates recruitment of CDKL5, additional experiments would be required to fully confirm that CDKL5 is recruited at transcription-associated DSB. We suggest to revise the title and modify sentences throughout the manuscript to be less assertive on this point.***
>Agreed. However, we are currently doing an additional experiments, which may confirm CDKL5 is recruited at transcription-associated DSB (see below).

2) **It is unclear whether CDKL5-mediated phosphorylation of ELOA is DNA damage-dependent. Additionally, it would be useful to know whether unphosphorylated ELOA is recruited in a CDK5L-dependent manner at DNA lesions, or is it only the Ser311 phosphorylation at damage sites that is CDKL5 dependent?**
> Unfortunately, the ELOA pSer311 antibodies don't work well in western blotting experiments, and we've been unable to test if the phosphorylation is DNA damage dependent. However, since both CDKL5 and ELOA are recruited to DNA damage sites by similar mechanism, we speculate that their juxtaposition enables ELOA phosphorylation at DNA damage sites.
> We have found that ELOA is recruited to DNA damage sites with the same apparent efficiency in CDKL5 KO cells as in parental cells, implying that phosphorylation of Ser311 is

not important for recruitment. We will present these data in the revised paper, and before resubmission we will analyse recruitment of an ELOA Ser311A mutant.

3) ***The authors present functional data on CDKL5 and show that it is implicated in the transcriptional repression that occurs post-DSB induction. Yet this part of the manuscript is somehow disconnected to the previous part where the relationship between CDKL5 and ELOA is made. Is transcriptional repression dependent on ELOA and/or on the Ser311 phosphorylated form of ELOA?***
> To address the disconnect the referee mentions, we are attempting to carry out rescue-type experiments to test if the kinase activity of CDKL5 is required for I-PpoI-induced gene silencing. > We are attempting to carry out rescue-type experiments to test if the kinase activity of CDKL5 is required for I-PpoI-induced gene silencing. This has proven to be technically tricky but we are working on it.
>We haven't yet looked the consequence of the ELOA phospho-site mutant. In other projects we're doing, it's the phosphorylation of multiple targets that combine to give a net functional output and it's possible that the same applies here. We are, however, testing whether the kinase activity of CDKL5 is required for the role in transcriptional silencing at DSB as mentioned above.

4) ***While CDKL5 is recruited at sites of laser microirradiation, it would be useful if the authors could show its recruitment at FokI-induced DNA damage.***
> We have been trying this experiment, but the transient nature of CDKL5 association with breaks make the analysis difficult, as other haver found for PAR-dependent proteins like XRCC1. However, we have recently observed CDKL5 recruitment to FokI-induced DSB in a small proportion of cells (maybe because of the transient recruitment), and we're doing some extra controls at present. In preliminary experiments recruitment occurs only when transcription is turned on at the DSB. We're currently doing the experiments, and if we obtain reproducible, convincing data then we will include them in the revised paper.

Regarding CDKL5 recruitment at DNA damage, the data presented Fig. 1G, are puzzling, since GFP-NLS alone behaves as GFP-NLS-CDKL5 (increases post H202). It looks like there is a mistake in the annotation (GFP-CDKL5: 5 cells shown without H202, 4 cells only with H202 and 6 with PARPi). If not, it seems difficult from the data presented here to conclude that CDKL5-GFP is recruited at DNA damage. Finally, did the authors assessed whether endogenous CDKL5 also localizes to laser micro-irradiation or H2O2-induced damage?
>We're very sorry that there's a mistake in Fig. 1G – the middle GFP-NLS control panel is mis-labelled. The amended figure is attached.
>We have tried many experiments to look at endogenous CDKL5. We have tried all of the commercially available antibodies, using CDKL5 siRNA and CDKL5 KO cells as a control for antibody specificity. However, none of the antibodies recognised CDKL5 specifically. The antibodies we raised in sheep don't work in immunofluorescence. This problem is reminiscent of XRCC1, which has proven very difficult to visualize in recruitment experiments at the endogenous level.
>We have, however, used antibodies that we raised against a CDKL5 autophosphorylation site – Y171 (these were described in our 2018 EMBO J CDKL5 paper). These antibodies recognize laser micro-irradiation tracks, and a series of careful control experiments indicate the signal comes from the phosphorylated form of endogenous CDKL5. We will include these data in the revised version of the paper, if further control experiments prove beyond doubt it's really endogenous CDKL5 we're detecting.

Minor points – we can respond to all of these points by amending the text.

Figure_1

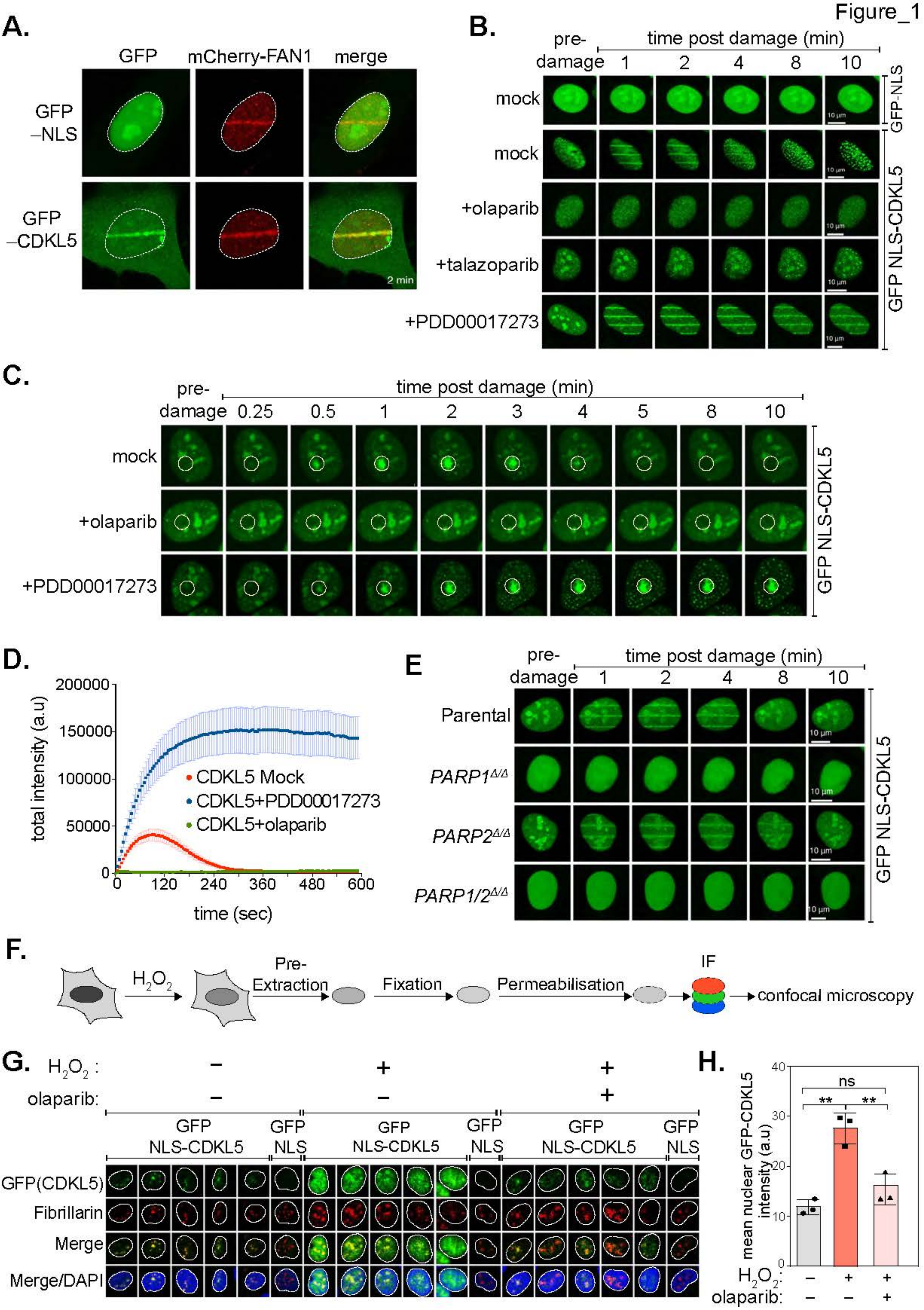

A.
GFP
mCherry-FAN1
merge
GFP –NLS
GFP –CDKL5
2 min
B.
pre-damage
time post damage (min)
1
2
4
8
10
mock
mock
+olaparib
+talazoparib
+PDD00017273
GFP-NLS
GFP NLS-CDKL5
10 µm
C.
pre-damage
time post damage (min)
0.25
0.5
1
2
3
4
5
8
10
mock
+olaparib
+PDD00017273
GFP NLS-CDKL5
D.
total intensity (a.u)
time (sec)
CDKL5 Mock
CDKL5+PDD00017273
CDKL5+olaparib
E
pre-damage
time post damage (min)
Parental
PARP1Δ/Δ
PARP2Δ/Δ
PARP1/2Δ/Δ
GFP NLS-CDKL5
F.
H2O2
Pre-Extraction
Fixation
Permeabilisation
IF
confocal microscopy
G.
H2O2 :
olaparib:
GFP NLS-CDKL5
GFP NLS
GFP(CDKL5)
Fibrillarin
Merge
Merge/DAPI
H.
mean nuclear GFP-CDKL5 intensity (a.u)
ns
**
H2O2:
olaparib:

1st Editorial Decision 19th Apr 2021

Thank you for tentative point-by-point response to the referee reports, and proposal for revising this work for The EMBO Journal. I have now had a chance to consider these plans, and appreciate that they should likely clarify the technical/experimental issues (particularly raised by referee 1), as well as deepen the functional understanding as asked by referees 2 and 3. With the incorporation of these revisions, we would therefore be happy to consider this work further as a primary EMBO Journal research article. Key aspects for the revised manuscript would be the proposed experiments on CDKL5 activity/ELOA phosphorylation on CUL5/CSB interactions, on whether CDKL5 activity is required for damage-induced silencing, and on (endogenous/phospho) CDKL5 damage recruitment. I also agree that incorporating your unpublished data on consensus motif refinement would be valuable.

Responses to Referees' Comments
New data added:
Fig. 7B
Figs. 8B&C
Fig. EV2E
Fig. EV4E
Other data obtained during revision, which are not included in the revised paper are shown and/or discussed below.

Response to Referee 1
We are grateful to Referee 1 for their insightful comments, especially on the mass spectrometric data analysis, which lacked clarity in the original version.

General comments:
A key question is: how do they know that any of the differentially regulated phosphorylation sites are genuine CDKL5 targets, as opposed to indirect substrates being modulated by a CDKL5-reguated kinase? While the HEK293 validation of potential CDKL5 substrates does not actually rule out these proteins being phosphorylated indirectly by a kinase that itself is regulated by CDKL5, the in vitro assays with peptide substrates do support this hypothesis, at least for Ser729 on EP400 and Ser311 on ELOA. Given the possibility for indirect regulation in both the HTP screen and the 293 cell experiments, the authors need to tone down their language to reflect this possibility.
In our 2018 paper (PMID: 30266825), we noticed that the phosphorylated residues in the cytosolic substrates which we validated all occurred in the motif R-P-X-S*-A; the phosphorylated residues in the CDKL5 substrates validated by Sila Ultanir in a back-to-back paper also lay in the motif R-P-X-S*-A. Using synthetic peptides corresponding to sequence around the CDKL5 phosphorylation site in MAP1S, we narrowed down the CDKL5 specificity determinant (consensus) to R-P-X-[S/T]*-[A/G/P] – but of course the requirement for A/G/P at the +1 position came from *in vitro* data. Since those data were published, we substituted the A at the +1 position the MAP1S peptide to every other amino acid, which refined the CDKL5 consensus to R-P-X-[S/T]*-[A/G/P/S]. But we had no idea if this was relevant to CDKL5 in intact cells. In our current study, the R-P-X-[S/T]*-[A/G/P/S] motif occurs in 17 of the 31 unique phospho-peptides ($\geq$75% site localisation probability, single phosphorylation site) that are more abundant in CDKL5-NLS-WT samples compared with the CDKL5-NLS-KD samples. This observation strongly suggests, but does not prove, that the targets we identified are direct CDKL5 targets. We have modified the text, and discussed this point, and we have included the experiment with the synthetic MAP1S peptides in revised Fig EV2E.

How do the authors explain identification of cytoplasmic substrates given the time course of these studies and the targeted nuclear localisation CDKL5?
The nuclear enrichment protocols we used simply enrich nuclear proteins, they don't result in a pure nuclear fraction, so the presence of proteins from other compartments is not unexpected.

The authors describe ELOA pSer311 as a potential characteristic biomarker of CDD, based on decreased observation in cell lines containing CDD-associated CDKL5 mutations. I think this is oversimplifying the situation and assumes that this residue is not modified by any other kinase, and that there is no effect of a phosphatase that would mask this effect. In the absence of validation with clinical samples, this statement should be removed.
Given that we have obtained evidence of CDKL5-dependent phosphorylation of ELOA Ser^{311} we think it is not unreasonable to describe ELOA as a <u>potential</u> biomarker, which is how we describe ELOA in the revised text. The point about other kinases is well taken; however, in cells where we disrupted CDKL5, phosphorylation of ELOA Ser311 at sites of laser micro–

irradiation was markedly reduced, and so in these cells Ser311 does not appear to be modified by other kinases to the same degree as by CDKL5 at least under these limited conditions. The situation might be different in human tissue samples of course, but we still feel it's appropriate to describe ELOA as a potential biomarker for CDKL5 activity worthy of investigation.

The final series of experiments demonstrate that knock-down of CDKL5 weakens the silencing of transcriptional near DNA breaks. However, there is no evidence currently that this is actually dependent on CDKL5 kinase activity. While they do not state this to be the case, evaluation of KD versus WT CDKL5 in this regard would bring the manuscript together based on their original hypothesis.
We carried out rescue-type experiments, by expressing siRNA-resistant forms of CDKL5 (wild-type or kinase-dead) to test if the kinase activity of CDKL5 is required for I-PpoI-induced transcriptional repression. These experiments showed clearly that the kinase activity of CDKL5 is required for I-PpoI-induced gene silencing. The new data are shown in revised Figs. 8B and C.

Technical concerns
1. ***The total number of phosphopeptide identified are details, but there is no mention of how many phosphopeptides (or sites) were significantly regulated as a function of expression of inactive CDKL5. What proportion of these were nuclear?***
Based on analyses described in point 2 below, we identified 37 peptides (31 unique peptide sequences, 24 unique proteins) which were higher in abundance in the CDKL5-NLS-WT samples compared with the CDKL5-NLS-KD samples. 17 out of 24 proteins are known to be nuclear (or "nucleus-associated") in localization (GO: 0005634 (nucleus and nucleus-offspring GO terms)).

2. ***There is no mention in the methods section of how (or if!) the phosphoproteomics data were filtered for site localisation confidence. This is particularly relevant as they are applying a 5% FDR, rather than the more typical 1% FDR. 223 phosphopeptides with p-value<0.01 (2079 at p<0.05) were in this list and a cursory glance through these would suggest that a relatively small number are above the typically applied 0.75 PTM-score threshold (assuming this is PTM-score that they are using), let alone the 0.994 value that equates to a 1% FLR as suggested in Ferries et al JPR (2017). I'm not sure what justification the authors have for selecting a p-site probability of 0.6 as they have stated in the legend to Fig 4. They need to be explicit about which particular score they apply, and justification for using a 0.6 cut-off.***
We apologize that there was a mistake in the original manuscript, which has caused major confusion. The line on page 9 "CDKL5–NLS–WT cells compared with CDKL5–NLS–KD cells (>1.5–fold, p<0.05)" would, as the Referee mentioned, have resulted in the 2079 phospho-peptides. However, this line should have read "CDKL5–NLS–WT cells compared with CDKL5–NLS–KD cells (>1.5–fold, p < 0.0005)". In essence, we analysed the volcano plot shown in Figure 4B, which clearly revealed 37 phospho-peptides (31 unique sequences) that were higher in abundance in the CDKL5-WT samples compared with the KD samples; this group clustered away from the bulk of phospho-peptides, and all the phospho–peptides within this cluster all had p < 0.0005. These 37 phospho-peptides were assigned as the peptides of interest. We state this clearly now in the Results section, and we have amended the Methods section too, and we have corrected the p-value typo.

Within the group of 31 unique sequences, an RPX[S/T] sequence motif occurs 25 times. In 19 out of these 25 cases, Andromeda assigned a phosphorylation site unique within the protein to the RPXp[S/T] motif. When a 75% filter is applied and only singly phosphorylated peptides are selected, the assignment of 16 of these unique phosphorylated sites peptides remains (removing POM121C, NCOA5 and RBM12B). When a 100% filter is applied, the assignment of 9 of these unique sites remains. The cut-off of 0.6 was chosen arbitrarily for

the sake of simplifying the table in Figure 4C. In the revised manuscript we have corrected the p-value typo, added an extra column in Fig. 4C depicting the actual site probabilities, and applied a probability filter of 75% as well as indicating 1% FLR.

3. ***Where site localisation is ambiguous (i.e. below 0.75 or 0.994 dependent on what cut-off you select), this needs to be explicitly stated in any downstream analysis, and the data treated separately. Looking at the Supp. Tables, they do not appear to have filtered based on site localisation at all, but overall this is confusing - for example, GLVRPGS(0.5)S(0.5)R is identified from P49750 where Ser at positions 7 or 8 are equally as likely to be phosphorylated (hence both have a localisation score of 0.5), yet the phosphorylated residue that appears to have been considered is Ser7. There are numerous similar examples - I am thus concerned based on this that the authors may be cherry-picking their data, as they haven't provided sufficient explanation of what they have actually done.***
In the specific case listed by the Referee: protein P49750 – called ZAP3/YLPM1, several closely-related peptides were present in the phospho-proteomics dataset in Table EV1, from different RP chromatography fractions: GLVRPGS(0.5)S(0.5)R (fold-change 13.08, p = 4.47E-10); GLVRPGS(0.101)S(0.899)R (fold-change 6.23, p = 8.96E-08); DRGLVRPGS(0.877)S(0.123)R (fold-change 4.42, p = 2.64E-09). The numbers in brackets in the peptide sequence are the phospho-site probabilities assigned by Andromeda. Based on these numbers, the referee rightly points out that it's not possible to know if it's the first or second serine in this motif that's phosphorylated by CDKL5 – it could be just one, or both. However, the first serine lies in a motif similar to the CDKL5 consensus motif. But even knowing this, we showed ZAP3 twice in the table in Figure 4C, each with one of the two serines highlighted in red. That's why we're disappointed the referee thinks we're cherry picking, because we were careful to highlight the second serine.

In the revised manuscript, we have added an extra column in revised Figure 4C showing the localisation site probabilities and also applied a site localisation probability filter of 75%, as well as a 1% FLR. We have also revised Table EV1 and added a 75% phospho–site localization filter as a well a 1% FLR filter column labelled "Site Probability ≥ 75%" and "Site probability ≥ 99.4% (1% FLR)". In addition, extra columns were added to Table EV1 for assignment of an RPXp[S/T][A/G/P/S] motif (denoted "Phosphorylation site part of RPX[S,T][A,G,P,S] motif (≥ 75%)?" and "Phosphorylation site part of RPX[S,T][AGPS]motif (≥ 99.4%)?", respectively).

4. ***As a general point, it is unclear how they are filtering the data presented in Fig. 4C. It is more important to filter by statistical probability of a site being regulated, rather than the fold change, which will also be influenced potentially by the activity of other kinases/phosphatases. I am not a fan of fold-change filtering is appropriate statistical cut-offs are applied.***
After much discussion we opted not to present our data filtered based on a pre-defined statistical significance cut-off. We were influenced, for example, by recent considerations on the potential pitfalls associated with this approach (see Figure 4 and discussion in: *Blume JD, Greevy RA, Welty VF, Smith JR & Dupont WD (2019) An Introduction to Second-Generation p-Values. Am Stat **73**: 157–167*). Therefore, we simply analysed the volcano plot as described in the response to point 2 above and focussed on the 37-member phospho-peptide group that clustered away from the bulk.
>The arbitrary cut-off of fold-change > 1.5 in Figure 4C was chosen for the sake of simplifying the table; the full dataset is shown in Table EV1.
>We agree that the sites identified in this study could be targets of other kinases/phosphatases. But we argue that we're looking specifically at CDKL5-dependent phosphorylation of individual sites in our study.
>Applying a 1% FDR cut-off to the data as the Referee suggested, leaves 18 unique peptides of interest. The approach we took showed 31 unique peptides of interest. One of

these (TTDN1) would have been excluded by the 1% FDR cut-off, and yet this was one of the proteins validated as being phosphorylated in a CDKL5-dependent manner in the XIC experiments. On balance therefore, we've opted not to filter based 1%FDR. We hope the Referee concurs with our choice.

5. *Can you please provide details on imputation of null values. The volcano plot would suggest that this was done, but this is not explained.*
Out of 90,867 peptides detected in our phospho–proteomic dataset (after excluding peptides with >5% sequence identification FDR, removal of potential contaminants as flagged by MaxQuant, parent ion fraction < 0.60 and reverse database hits), 100 peptides in the KD group and 64 peptides in the WT group had one or more missing datapoints which were assigned "NA". Peptides with only a single datapoint within KD (8 peptides) and/or WT groups (6 peptides) were removed prior to statistical analysis (10 peptides in total).

6. *What proportion of all phosphorylation sites, versus the statistically significantly regulated phosphosites match the consensus (considering a suitable oFLR of >0.75). To increase confidence of this being a CDKL5 consensus motif, you should show enrichment of those sites that are regulated against a background of all identified phosphosites.*
Within the group of 31 unique peptides of interest, there are 19 unique phospho-sites derived from singly phosphorylated peptides with putative phosphorylation sites in the form of an RPXp[S/T] motif (as assigned by Andromeda). Applying a 75% filter, leaves 16 unique phospho-sites. Applying a filter setting of 100% leaves 9 unique sites with an RPXp[S/T] motif. All of these RPXp[S/T] motifs have A, G, P or S at the +1 position.
>Within the complete dataset (28,185 unique phosphorylation sites with a site localisation probability of $\geq$ 75%) 186 phosphorylation sites exhibit an RPXp[S/T] motif. When only taking the 100% filtered RPXp[S/T] sites within our group of unique phosphorylation sites into account, this is a 52-fold enrichment of this motif as compared to the background (p = 8.44E-014 (Fisher's exact test)).

7. *Please provide some more details (supp. Table) on GO term enrichment (that will likely need to be re-done using the newly filtered dataset) - typically you would require 3 proteins per group.*
As mentioned above the line "CDKL5–NLS–WT cells compared with CDKL5–NLS–KD cells (>1.5–fold, p<0.05)" on page 9 should read "CDKL5NLS–WT cells compared with CDKL5NLS–KD cells (p < 0.0005)". For the gene ontology analyses, we extracted all GO terms attached to the 25 unique proteins identified as higher or lower abundant in WT by our volcano plot analysis (foreground) and tested for GO term enrichment against the background of 5985 identified proteins using a Fisher's exact test. As we only have 25 unique proteins as foreground, we chose a cut-off of at least two proteins per significant GO term. Most of the significant GO terms are closely related (Figure 4E), but we have changed this cut-off to three proteins as suggested by the Referee and amended Figure 4E accordingly. We have revised the Methods section to clarify this issue.

Response to Referee 2
We are grateful to Referee 2 for their insightful comments.
Major points:
(1) ***Elongin A performs dual functions as an RNA polymerase II (pol II) elongation factor and as the substrate recognition subunit of a Cullin-RING E3 ubiquitin ligase that targets pol II stalled at sites of DNA damage. It is already known that Elongin A and Cul5 are rapidly recruited in cells to regions of localized DNA damage, and that assembly of Elongin A and Cul5 is triggered following DNA damage as well as by treatment of cells with pol II inhibitors; alpha-amanitin and DRB (Weems JC et al. JBC 2015). In addition, Elongin A and Cul5 associate in cells with the Cockayne syndrome B (CSB) protein and this interaction is also induced by DNA damage, alpha-amanitin and DRB (Weems JC et al. JBC 2017).***
Does the phosphorylation of Elongin A by CDKL5 affect either of the above processes? This question needs to be answered by additional studies.
>We found that ELOA recruitment to micro–irradiation tracks does not appear to require phosphorylation by CDKL5. Recruitment of GFP–tagged wild-type ELOA occurs normally in in CDKL5 KO cells, and an ELOA S^{311} phospho–site mutant behaves like wild-type ELOA. These data are now shown in revised Fig. EV4E.
>We tested the impact of ELOA Ser311 phosphorylation on interaction with CUL5 or CSB. In co-transfection experiments the level of GFP–ELOA S^{311}A mutant interacting with FLAG–CUL5 was not significantly different from GFP–ELOA wild-type; also, the level of FLAG–ELOA S^{311}A mutant interacting with GFP–CSB was not significantly different from FLAG–ELOA wild-type. Moreover, the levels of endogenous CSB or endogenous CUL5 bound to GFP-ELOA immunoprecipitated from CDKL5–disrupted U2OS cells was similar to the parental cells. The data are shown in Figure R1 below. Therefore, in our hands, the phosphorylation of ELOA by CDKL5 does not appear to impact on the association of ELOA with CUL5 or CSB. These new data are included in a file for the Referee. On balance, we have not included these data in the revised paper; the reader may be left wondering why we concentrated arbitrarily on this aspect of ELOA function, when there are so many other angles we could have tested, and the data don't gel with the rest of the paper. We're open to including the data if necessary though.

Minor points:
(1) Since the amino acid sequences of Elongin A are highly conserved between human, rat (773 aa protein) and mouse (773 aa protein), I believe human Elongin A is composed of 772 amino acids (A0A024RAC6). Therefore, phosphorylation site of human Elongin A will be not Ser311, but Ser285. Ser311 in the sentences and figures may be better to be changed to Ser285.
> A0A024RAC6 appears to be an unreviewed Uniprot entry, corresponding to a short ELOA isoform (772 amino acids). In our study, we use the ELOA accession number Q14241 (798 aa) which is a reviewed entry in Uniprot. This is the cDNA we cloned and used. We therefore propose to keep the pSer311 annotation in the text, and we now provide the Uniprot accession number of ELOA, EP400 and TTDN1 in the Results section in the revised manuscript.
>We have amended the text to address the remaining minor points.

Response to Referee 3
We are grateful to Referee 3 for their insightful comments.
Main comments
1) ***Although the data clearly show that DRB/a-amanitin pre-treatment abrogates recruitment of CDKL5, additional experiments would be required to fully confirm that CDKL5 is recruited at transcription-associated DSB. We suggest to revise the title and modify sentences throughout the manuscript to be less assertive on this point.***
>We have obtained more evidence of recruitment to transcription-associated DNA damage, in the FokI reporter cells, which came about when testing recruitment of CDKL5 to FokI-

induced DSB. This is not easy, as the transient nature of CDKL5 association with breaks makes capturing CDKL5 at foci very difficult, as other researchers have found for PAR-dependent proteins like XRCC1.

To capture GFP–CDKL5 in live imaging experiments in the FokI reporter cells: multiple fields were quickly scanned just 15 min after inducing FokI nuclear translocation with 4-hydroxytamoxifen. Fields were scanned specifically to detect cells that showed co-localization of mCherry-FokI and GFP–CDKL5. Imaging was done for 10 min, making total time lapses of not more than 25 min post DSB induction. This short timeframe was necessary as CDKL5 is only transiently recruited to DNA damage sites; we included an inhibitor of PARG which should prolong retention at damage sites but not for long), and an ATM inhibitor which prevents transcriptional silencing. Even under these conditions, only around 5-20% of cells showed clear mCherry-FokI foci, and only around 3-5% cells showed the co-localisation of mCherry-FokI dots with GFP–tagged CDKL5. Again, we wouldn't have expected any other outcome because of the transience of CDKL5. We never saw GFP alone at mCherry-FokI foci. Furthermore, we failed to observe GFP-CDKL5 at mCherry-FokI foci in the absence of doxycycline treatment – in other words when transcription of the reporter was off. A major issue is that the low proportion of cells GFP-CDKL5 foci make it almost impossible to provide accurate quantitation, and we were unsure as to whether we should include the data in the revised paper. However, we saw GFP–CDKL5 foci in 5 biological replicates of the FokI experiment; we tested the impact of omitting doxycycline in three biological replicates of the experiment with similar results. Multiple technical replicates were done the same day per biological replicate. Therefore, we have included the data in revised Fig. 7B. We're happy to discuss this point; we have also revised the title and text as requested.

2) **It is unclear whether CDKL5-mediated phosphorylation of ELOA is DNA damage-dependent. Additionally, it would be useful to know whether unphosphorylated ELOA is recruited in a CDK5L-dependent manner at DNA lesions, or is it only the Ser311 phosphorylation at damage sites that is CDKL5 dependent?**
> Unfortunately, we've been unable to test if the phosphorylation of endogenous ELOA is DNA damage dependent. However, since both CDKL5 and ELOA are recruited to DNA damage sites by similar mechanism, we speculate that their juxtaposition enables ELOA phosphorylation at DNA damage sites.
> In the response above to Major Point 1 from Referee 2, we describe how we've found that have found that ELOA phosphorylation by CDKL5 does not appear to be required for ELOA recruitment to DNA damage sites (revised Fig. EV4E).

3) ***The authors present functional data on CDKL5 and show that it is implicated in the transcriptional repression that occurs post-DSB induction. Yet this part of the manuscript is somehow disconnected to the previous part where the relationship between CDKL5 and ELOA is made. Is transcriptional repression dependent on ELOA and/or on the Ser311 phosphorylated form of ELOA?***
> We have now carried out rescue-type experiments, by expressing siRNA-resistant forms of CDKL5 wild-type and kinase-dead to test if the kinase activity of CDKL5 is relevant for transcriptional repression at DSB. These experiments showed clearly that the kinase activity of CDKL5 is required for I-PpoI-induced gene silencing. The new data are shown in revised Figs 8B and C.
>We haven't yet looked the consequence of the ELOA phospho-site mutant. In other projects we're doing, it's the phosphorylation of multiple targets that combine to give a net functional output and it's possible that the same applies here. To rigorously address this question, it will be necessary to engineer homozygous knock–in phospho–site mutants, which we're starting to do. These studies, however, are beyond the scope of the current study in our opinion.

4) ***While CDKL5 is recruited at sites of laser microirradiation, it would be useful if the authors could show its recruitment at FokI-induced DNA damage.***
See response to Point 1.

Regarding CDKL5 recruitment at DNA damage, the data presented Fig. 1G, are puzzling, since GFP-NLS alone behaves as GFP-NLS-CDKL5 (increases post H202). It looks like there is a mistake in the annotation (GFP-CDKL5: 5 cells shown without H202, 4 cells only with H202 and 6 with PARPi). If not, it seems difficult from the data presented here to conclude that CDKL5-GFP is recruited at DNA damage. Finally, did the authors assessed whether endogenous CDKL5 also localizes to laser micro-irradiation or H2O2-induced damage?
>We're very sorry that there's a mistake in Fig. 1G – the middle GFP-NLS control panel was mis-labelled. The mistake has now been corrected.
>Visualizing recruitment of the endogenous counterparts of proteins recruited transiently is notoriously difficult and has not been possible for well-characterized PARP-dependent repair proteins such as XRCC1. In this light, we have tried many experiments to look at endogenous CDKL5 recruitment without success. We have tried all of the commercially available antibodies, using CDKL5 siRNA and CDKL5 KO cells as a control for antibody specificity. However, none of the antibodies recognised CDKL5 specifically in our hands. The antibodies we raised in sheep against total CDKL5 don't work in immunofluorescence analyses. We tested antibodies that we raised against a CDKL5 autophosphorylation site – Y^{171} (these were described in our 2018 EMBO Journal CDKL5 paper). We found that these antibodies weakly recognize laser micro-irradiation tracks, and the signal was modestly reduced in CDKL5 knockout U2OS cell clones, and by CDKL5 siRNAs. However, the reduction was modest at best, and was variable in different biological in replicates, perhaps due to recognition of the T-loop of kinases other than CDKL5 in cell extracts. In the end, we concluded that these data were not sufficiently strong to include as evidence of endogenous CDKL5 recruitment. In the past we have mNeon-tagged the endogenous CDKL5 locus but could not detect the tagged protein in IF experiments. So the detection of endogenous CDKL5 has not been possible. Perhaps raising rabbit monoclonal antibodies, which often display remarkably high sensitivity, will be a solution to this problem.

Minor points:
A) Different isoforms of CDK5L have been reported (PMID: 27315173). Could the authors specify in material and methods which isoform was used in this study?
*We used the longest isoform encoding the 1030 amino acid protein (*NM_003159.2 with silent changes t996c and t2118c), which we now state in the Methods section.
B) Could the authors provide a reference when citing APLF as a positive control for PAR binding?
This was an oversight on our part; we have now included a reference.
***C) The authors also need to describe in the Materials and methods the experimental* procedure for the fractionation experiment.**
We have now included a section describing the protocol used for fractionating cells**.**
D) When the authors mention the localization of ELOA to DNA damage (p11 last line), the wrong figures are cited. It should be Fig 6C-E instead of Fig4A-C.
This error has now been corrected.
E) Fig. 4B the x axis deserves better labelling. Is it the log2 fold change of WT/KD?
We have amended the x-axis in 4B accordingly.

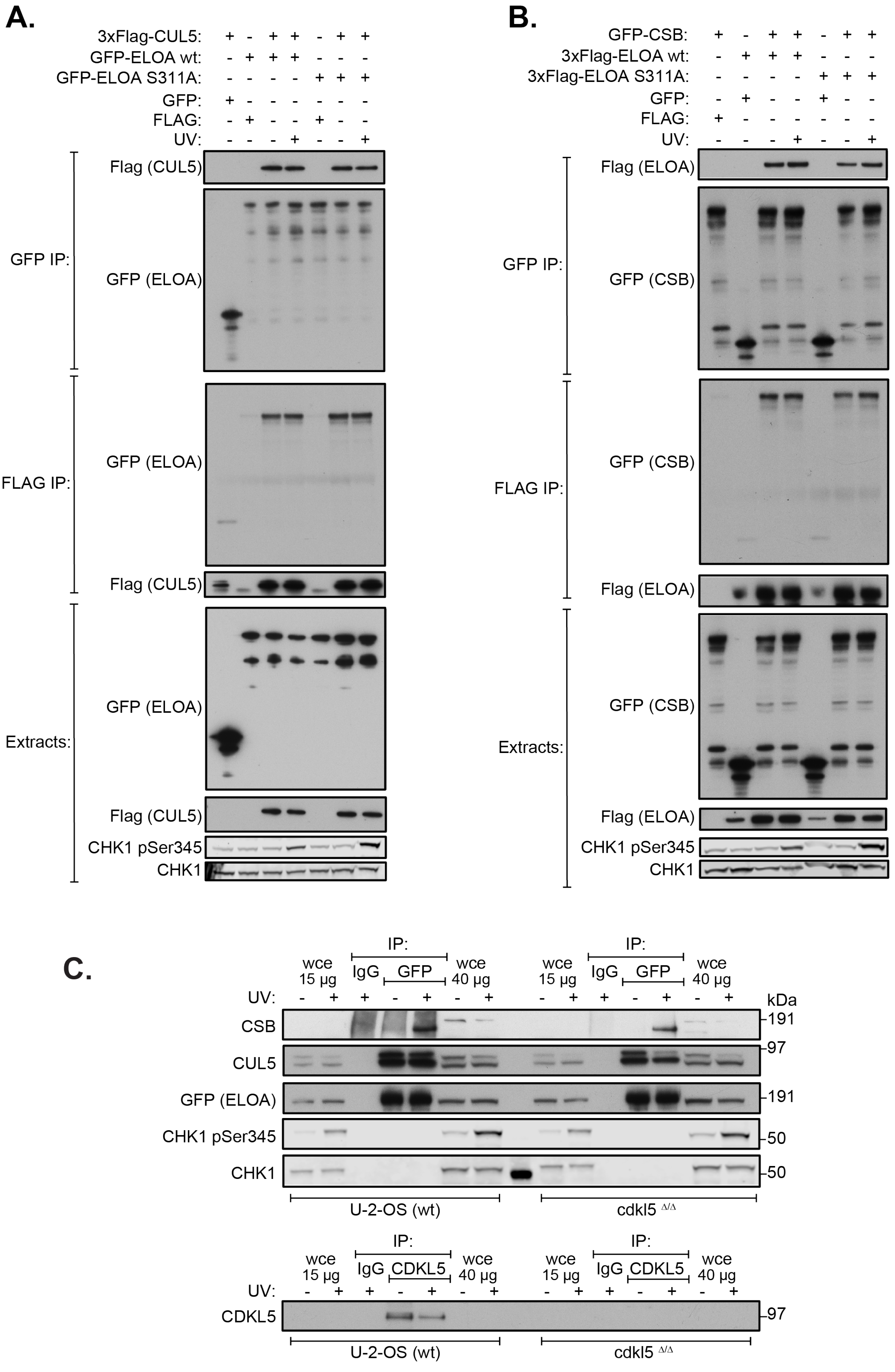
A.
3xFlag-CUL5:
GFP-ELOA wt:
GFP-ELOA S311A:
GFP:
FLAG:
UV:
Flag (CUL5)
GFP IP:
GFP (ELOA)
FLAG IP:
GFP (ELOA)
Flag (CUL5)
Extracts:
GFP (ELOA)
Flag (CUL5)
CHK1 pSer345
CHK1
B.
GFP-CSB:
3xFlag-ELOA wt:
3xFlag-ELOA S311A:
GFP:
FLAG:
UV:
Flag (ELOA)
GFP IP:
GFP (CSB)
FLAG IP:
GFP (CSB)
Flag (ELOA)
Extracts:
GFP (CSB)
Flag (ELOA)
CHK1 pSer345
CHK1
C.
IP:
wce 15 µg
IgG
GFP
wce 40 µg
UV:
kDa
CSB
CUL5
GFP (ELOA)
CHK1 pSer345
CHK1
191
97
191
50
50
U-2-OS (wt)
cdkl5 Δ/Δ
IP:
wce 15 µg
IgG
CDKL5
wce 40 µg
UV:
CDKL5
97
U-2-OS (wt)
cdkl5 Δ/Δ

Figure R1 Investigating the impact of ELOA phosphorylation on interaction with CSB and CUL5

A. and **B.** HEK293 cells transfected with the plasmids indicated (5 µg each) were either mock treated or exposed to UV-C (254 nm; 20 J/m^2) using a Boekel Scientific UV-C crosslinker. Cells were allowed to recover for 10 minutes before lysis, and extracts were subjected to anti–GFP or anti–FLAG immunoprecipitations as indicated. Precipitates were subjected to SDS-PAGE and western blotting with the antibodies indicated. The lower panel shows input extracts subjected to immunoblotting with indicated antibodies. **C.** Parental U–2–OS Flp-In T-Rex cells or *CDKL5*$^{\Delta/\Delta}$ cells stably expressing GFP–ELOA were mock treated or exposed to UV-C (254 nm; 20 J/m^2). Cells were allowed to recover for 10 minutes before lysis, and extracts were then subjected to immunoprecipitation with control IgG beads or anti–GFP beads as indicated. Precipitates were then subjected to SDS-PAGE and western blotting with the antibodies indicated. The lower panel shows CDKL5 immunoprecipitation from parental U–2–OS Flp-In T-Rex cells or *CDKL5*$^{\Delta/\Delta}$ cells followed by CDKL5 western blotting to show that CDKL5 cannot be detected in the *CDKL5*$^{\Delta/\Delta}$ cells.

1st Revision - Editorial Decision 27th Aug 2021

Thank you for submitting your revised manuscript to The EMBO Journal. We have now heard back from the three original referees, and I am pleased to say that they all found the previously-raised points satisfactorily addressed. Following a final revision round to incorporate some remaining minor issues noted in the comments, as well as the below-listed editorial points, we shall therefore be happy to accept the study for publication in our journal.

REFEREE REPORTS

Referee #1:

This manuscript is much improved over the previous version, with the authors having (attempted to) address most of my comments.
I welcomed the comments and clarification about how the (phospho)proteomics data were treated and agree with their explanation and implications of filtering by p-value/fold change. However, I think that there is still some confusion about site localisation scores versus site localisation probabilities. These two phrases appear to be have been used synonymously which they are not: e.g. a PTM-score of 0.994 does not equate to a localisation probability of 0.994, likewise, while they have state 75% site localisation probability, this actually refers to a PTM-score of 0.75 (so-called 'Class 1' phosphosites) (see e.g. Potel et al 2019). While application of a 0.75 or 0.994 cut off as used here is fine, the description of what these numbers actually refer to is not and needs to be corrected both in the Methods (not mentioned at all currently) and in the main text.

Referee #2:

The authors experimentally addressed all my questions in the revised manuscript, and I have no further points.

Referee #3:

In their revised manuscript, Khanam et al addressed most of the comments we made. They have now really strengthened their data regarding the function of CDKL5 at DSB occurring within transcribed regions by including experiment regarding the recruitment of CDKL5 at such damaged transcribed locus (FokI-assay) and by showing that the kinase activity of CDKL5 is required for I-PpoI-induced gene silencing. Therefore the manuscript now provides robust evidence for a function of CDKL5 in transcriptional silencing at DSB in actively transcribed regions, and it will be of interest to a broad audience. We recommend publication in EMBO Journal.

Reviewer 1:

However, I think that there is still some confusion about site localisation scores versus site localisation probabilities. These two phrases appear to be have been used synonymously which they are not: e.g. a PTM-score of 0.994 does not equate to a localisation probability of 0.994, likewise, while they have state 75% site localisation probability, this actually refers to a PTM-score of 0.75 (so-called 'Class 1' phosphosites) (see e.g. Potel et al 2019). While application of a 0.75 or 0.994 cut off as used here is fine, the description of what these numbers actually refer to is not and needs to be corrected both in the Methods (not mentioned at all currently) and in the main text.

Answer from the authors:

As far as we are aware “PTM score” is the name of the algorithm behind the calculation of the phosphorylation localisation probabilities in MaxQuant. However, there is a potential source of ambiguity: Within this algorithm, there is also a variable called “PTM score” which is calculated as $-10\log_{10}(p)$, where p is the probability of a specific (and possible) peptide/PTM configuration (i.e. the probability of matching (at least) k out of n masses by chance) (Olsen et al. 2006, Supplementary File S1: Page 3; PMID: 17081983; see also: Cox et al. 2011; PMID: 21254760 “Andromeda score”). Thus, the PTM score within the PTM score algorithm is not a probability itself. The reason the p-value is transformed into a PTM score is mostly historical: The original implementation of this algorithm used the $-10\log_{10}(p)$ transformation to obtain scores which were comparable to Mascot scores (Olsen & Mann 2004; PMID: 15347803). Mascot itself implemented this transformation to overcome the “inconvenience” of expressing p-values spanning orders of magnitude in scientific notation (Perkins et al. 1999; PMID: 10612281). However, MaxQuant does utilize the PTM score for the PTM score algorithm (minimum 40 and a delta of 6 between top hit and next best sequence (as per default settings, which we used)). The output of the PTM score algorithm is a site localisation probability and is calculated as the sum of the proportional, inverted probabilities over this selected set of possible peptide/PTM configurations (Olsen et al. 2006, Figure S6; PMID: 17081983). Potential phosphorylation sites with “Localization PTM probabilities” > 75% are defined as class I sites.

For phosphosite localisation probabilities we are using the column “Phospho (STY) Probabilities” from the MaxQuant “evidence.txt” output file, which is the “Sequence representation of the peptide including PTM positioning probabilities ([0..1], where 1 is best match) for 'Phospho (STY)'.” as per the “tables.pdf” output file. These values are also found in the column “Localisation Prob” in the “Phospho(STY).txt” output file which is mentioned in Tyanova et al. 2016 (PMID: 27809316) as “[…]Qualitative information, including score, PEP, **localization probability** and occupancy error, can be used to filter the table. For example, to limit the downstream analysis to **class I sites, a localization probability filter of >0.75** can be applied. […]”.

We therefore are going to add the expression “PTM-Score probability”, as used in the paper from Marx et al. (2013) (PMID: 23685481), to avoid any confusion between the output of the PTM score algorithm (localisation probability) and the actual PTM score (transformed p-value).

ACCEPTED **9th Sep 2021**

Thank you for submitting your final revised manuscript for our consideration. I am pleased to inform you that we have now accepted it for publication in The EMBO Journal.

EMBO PRESS

YOU MUST COMPLETE ALL CELLS WITH A PINK BACKGROUND

PLEASE NOTE THAT THIS CHECKLIST WILL BE PUBLISHED ALONGSIDE YOUR PAPER

Corresponding Author Name: John Rouse
Journal Submitted to: EMBO JOURNAL
Manuscript Number: EMBOJ-2021-108271

Reporting Checklist For Life Sciences Articles (Rev. June 2017)

This checklist is used to ensure good reporting standards and to improve the reproducibility of published results. These guidelines are consistent with the Principles and Guidelines for Reporting Preclinical Research issued by the NIH in 2014. Please follow the journal's authorship guidelines in preparing your manuscript.

A- Figures

1. Data

The data shown in figures should satisfy the following conditions:

- the data were obtained and processed according to the field's best practice and are presented to reflect the results of the experiments in an accurate and unbiased manner.
- figure panels include only data points, measurements or observations that can be compared to each other in a scientifically meaningful way.
- graphs include clearly labeled error bars for independent experiments and sample sizes. Unless justified, error bars should not be shown for technical replicates.
- if n< 5, the individual data points from each experiment should be plotted and any statistical test employed should be justified
- Source Data should be included to report the data underlying graphs. Please follow the guidelines set out in the author ship guidelines on Data Presentation.

2. Captions

Each figure caption should contain the following information, for each panel where they are relevant:

- a specification of the experimental system investigated (eg cell line, species name).
- the assay(s) and method(s) used to carry out the reported observations and measurements
- an explicit mention of the biological and chemical entity(ies) that are being measured.
- an explicit mention of the biological and chemical entity(ies) that are altered/varied/perturbed in a controlled manner.

- the exact sample size (n) for each experimental group/condition, given as a number, not a range;
- a description of the sample collection allowing the reader to understand whether the samples represent technical or biological replicates (including how many animals, litters, cultures, etc.).
- a statement of how many times the experiment shown was independently replicated in the laboratory.
- definitions of statistical methods and measures:
  - common tests, such as t-test (please specify whether paired vs. unpaired), simple χ2 tests, Wilcoxon and Mann-Whitney tests, can be unambiguously identified by name only, but more complex techniques should be described in the methods section;
  - are tests one-sided or two-sided?
  - are there adjustments for multiple comparisons?
  - exact statistical test results, e.g., P values = x but not P values < x;
  - definition of 'center values' as median or average;
  - definition of error bars as s.d. or s.e.m.

Any descriptions too long for the figure legend should be included in the methods section and/or with the source data.

In the pink boxes below, please ensure that the answers to the following questions are reported in the manuscript itself. Every question should be answered. If the question is not relevant to your research, please write NA (non applicable). We encourage you to include a specific subsection in the methods section for statistics, reagents, animal models and human subjects.

USEFUL LINKS FOR COMPLETING THIS FORM

http://www.antibodypedia.com
http://1degreebio.org
http://www.equator-network.org/reporting-guidelines/improving-bioscience-research-repor
http://grants.nih.gov/grants/olaw/olaw.htm
http://www.mrc.ac.uk/Ourresearch/Ethicsresearchguidance/Useofanimals/index.htm
http://ClinicalTrials.gov
http://www.consort-statement.org
http://www.consort-statement.org/checklists/view/32-consort/66-title
http://www.equator-network.org/reporting-guidelines/reporting-recommendations-for-tumo
http://datadryad.org
http://figshare.com
http://www.ncbi.nlm.nih.gov/gap
http://www.ebi.ac.uk/ega
http://biomodels.net/
http://biomodels.net/miriam/
http://jjj.biochem.sun.ac.za
https://osp.od.nih.gov/biosafety-biosecurity-and-emerging-biotechnology/
http://www.selectagents.gov/

B- Statistics and general methods

Please fill out these boxes (Do not worry if you cannot see all your text once you press return)

1.a. How was the sample size chosen to ensure adequate power to detect a pre-specified effect size?	
1.b. For animal studies, include a statement about sample size estimate even if no statistical methods were used.	NA
2. Describe inclusion/exclusion criteria if samples or animals were excluded from the analysis. Were the criteria pre-established?	NA
3. Were any steps taken to minimize the effects of subjective bias when allocating animals/samples to treatment (e.g. randomization procedure)? If yes, please describe.	NA
For animal studies, include a statement about randomization even if no randomization was used.	NA
4.a. Were any steps taken to minimize the effects of subjective bias during group allocation or/and when assessing results (e.g. blinding of the investigator)? If yes please describe.	NA
4.b. For animal studies, include a statement about blinding even if no blinding was done	NA
5. For every figure, are statistical tests justified as appropriate?	Yes (script file, supplementary data and source data)
Do the data meet the assumptions of the tests (e.g., normal distribution)? Describe any methods used to assess it.	Yes (script file, supplementary data and source data)
Is there an estimate of variation within each group of data?	Yes (script file, supplementary data and source data)

Is the variance similar between the groups that are being statistically compared?	Yes (script file, supplementary data and source data)

C- Reagents

6. To show that antibodies were profiled for use in the system under study (assay and species), provide a citation, catalog number and/or clone number, supplementary information or reference to an antibody validation profile. e.g., Antibodypedia (see link list at top right), 1DegreeBio (see link list at top right).	Yes (see Table EV2)
7. Identify the source of cell lines and report if they were recently authenticated (e.g., by STR profiling) and tested for mycoplasma contamination.	Yes (see materials and methods)

* for all hyperlinks, please see the table at the top right of the document

D- Animal Models

8. Report species, strain, gender, age of animals and genetic modification status where applicable. Please detail housing and husbandry conditions and the source of animals.	NA
9. For experiments involving live vertebrates, include a statement of compliance with ethical regulations and identify the committee(s) approving the experiments.	NA
10. We recommend consulting the ARRIVE guidelines (see link list at top right) (PLoS Biol. 8(6), e1000412, 2010) to ensure that other relevant aspects of animal studies are adequately reported. See author guidelines, under 'Reporting Guidelines'. See also: NIH (see link list at top right) and MRC (see link list at top right) recommendations. Please confirm compliance.	NA

E- Human Subjects

11. Identify the committee(s) approving the study protocol.	NA
12. Include a statement confirming that informed consent was obtained from all subjects and that the experiments conformed to the principles set out in the WMA Declaration of Helsinki and the Department of Health and Human Services Belmont Report.	NA
13. For publication of patient photos, include a statement confirming that consent to publish was obtained.	NA
14. Report any restrictions on the availability (and/or on the use) of human data or samples.	NA
15. Report the clinical trial registration number (at ClinicalTrials.gov or equivalent), where applicable.	NA
16. For phase II and III randomized controlled trials, please refer to the CONSORT flow diagram (see link list at top right) and submit the CONSORT checklist (see link list at top right) with your submission. See author guidelines, under 'Reporting Guidelines'. Please confirm you have submitted this list.	NA
17. For tumor marker prognostic studies, we recommend that you follow the REMARK reporting guidelines (see link list at top right). See author guidelines, under 'Reporting Guidelines'. Please confirm you have followed these guidelines.	NA

F- Data Accessibility

18: Provide a "Data Availability" section at the end of the Materials & Methods, listing the accession codes for data generated in this study and deposited in a public database (e.g. RNA-Seq data: Gene Expression Omnibus GSE39462, Proteomics data: PRIDE PXD000208 etc.) Please refer to our author guidelines for 'Data Deposition'.<br><br>Data deposition in a public repository is mandatory for:<br>a. Protein, DNA and RNA sequences<br>b. Macromolecular structures<br>c. Crystallographic data for small molecules<br>d. Functional genomics data<br>e. Proteomics and molecular interactions	Yes (provided in materials and methods section)
19. Deposition is strongly recommended for any datasets that are central and integral to the study; please consider the journal's data policy. If no structured public repository exists for a given data type, we encourage the provision of datasets in the manuscript as a Supplementary Document (see author guidelines under 'Expanded View' or in unstructured repositories such as Dryad (see link list at top right) or Figshare (see link list at top right).	DATA deposited in PRIDE
20. Access to human clinical and genomic datasets should be provided with as few restrictions as possible while respecting ethical obligations to the patients and relevant medical and legal issues. If practically possible and compatible with the individual consent agreement used in the study, such data should be deposited in one of the major public access-controlled repositories such as dbGAP (see link list at top right) or EGA (see link list at top right).	NA
21. Computational models that are central and integral to a study should be shared without restrictions and provided in a machine-readable form. The relevant accession numbers or links should be provided. When possible, standardized format (SBML, CellML) should be used instead of scripts (e.g. MATLAB). Authors are strongly encouraged to follow the MIRIAM guidelines (see link list at top right) and deposit their model in a public database such as Biomodels (see link list at top right) or JWS Online (see link list at top right). If computer source code is provided with the paper, it should be deposited in a public repository or included in supplementary information.	R-SCRIPTS Zenodo links provided in materials and methods

G- Dual use research of concern

22. Could your study fall under dual use research restrictions? Please check biosecurity documents (see link list at top right) and list of select agents and toxins (APHIS/CDC) (see link list at top right). According to our biosecurity guidelines, provide a statement only if it could.	NA
