# Supplementary material for: CDKL5 kinase controls transcription‐coupled responses to DNA damage
Source: EMBO J. 2021 Oct 4;40(23):e108271. doi: 10.15252/embj.2021108271 (PMC8634139; doi:10.15252/embj.2021108271)
Supplement: Supplementary file 6 — Source Data for Expanded View [file EMBJ-40-e108271-s003.zip › EMBOJ-2021-108271R1-Figure_EV5_Source_Data-sd.zip!/Figure EV5/Source data_FIG EV5 A.pdf]

Fig EV5 A

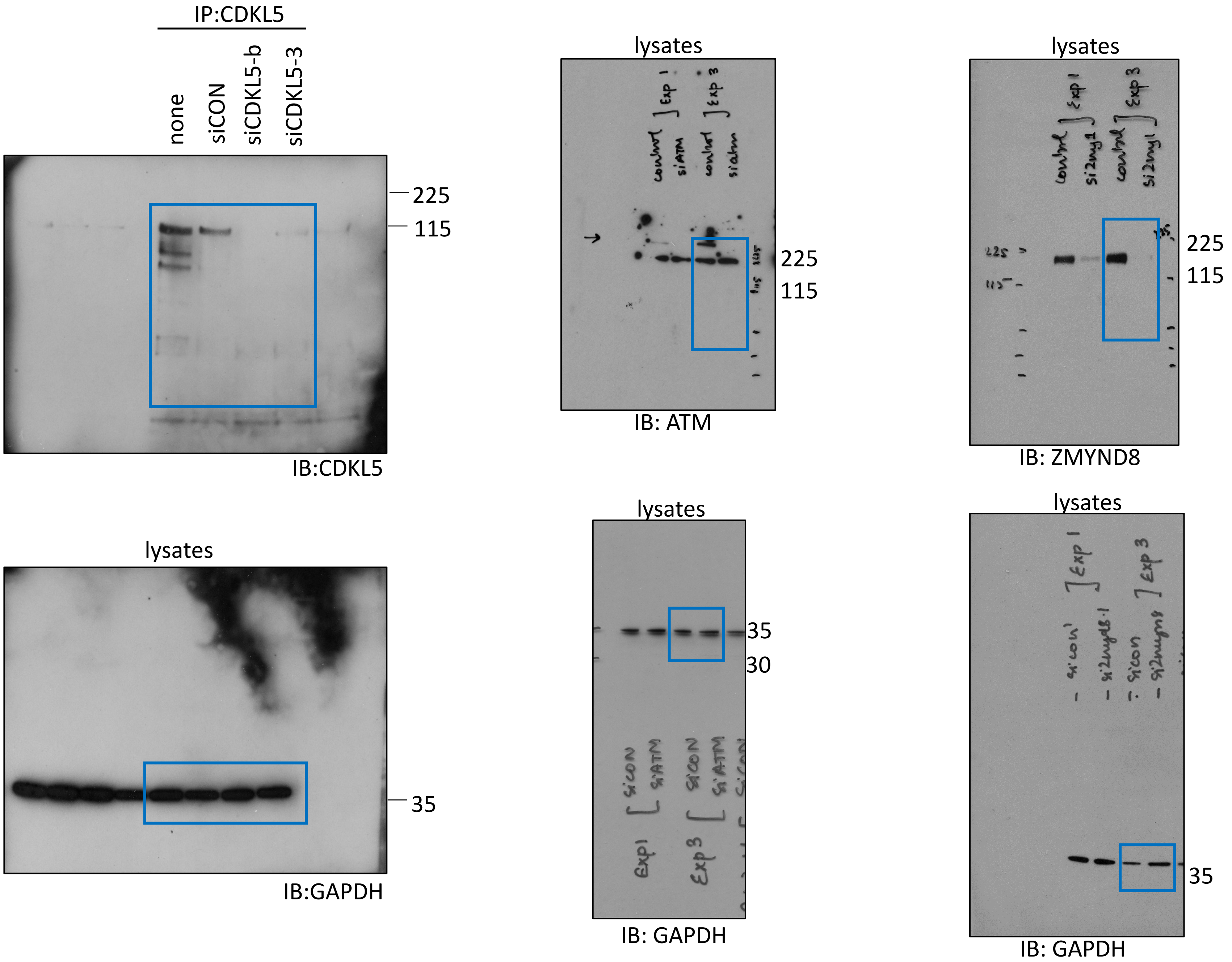
IP:CDKL5
none
siCON
siCDKL5-b
siCDKL5-3
225
115
IB:CDKL5
lysates
35
IB:GAPDH
lysates
225
115
IB: ATM
lysates
35
30
IB: GAPDH
lysates
225
115
IB: ZMYND8
lysates
35
IB: GAPDH
