# Supplementary material for: CDKL5 kinase controls transcription‐coupled responses to DNA damage
Source: EMBO J. 2021 Oct 4;40(23):e108271. doi: 10.15252/embj.2021108271 (PMC8634139; doi:10.15252/embj.2021108271)

Figure EV2

A.

CDKL5

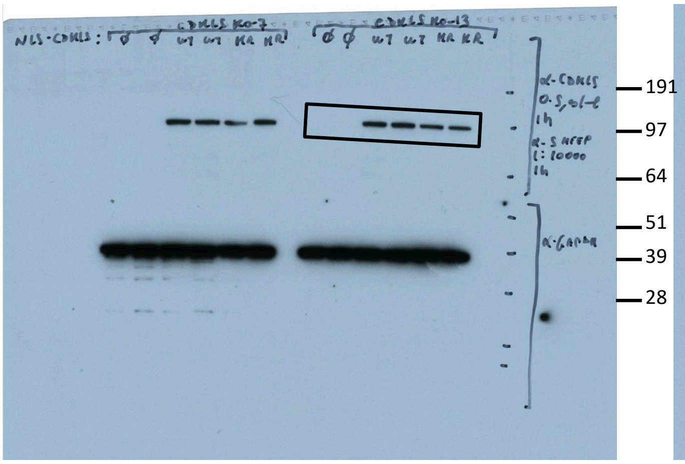

GAPDH

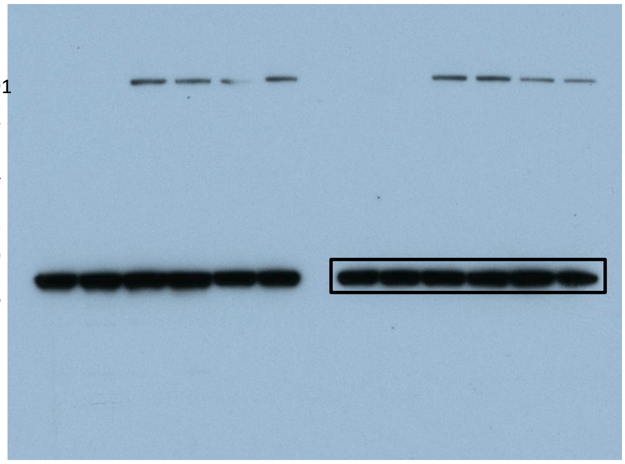

B.

CDKL5

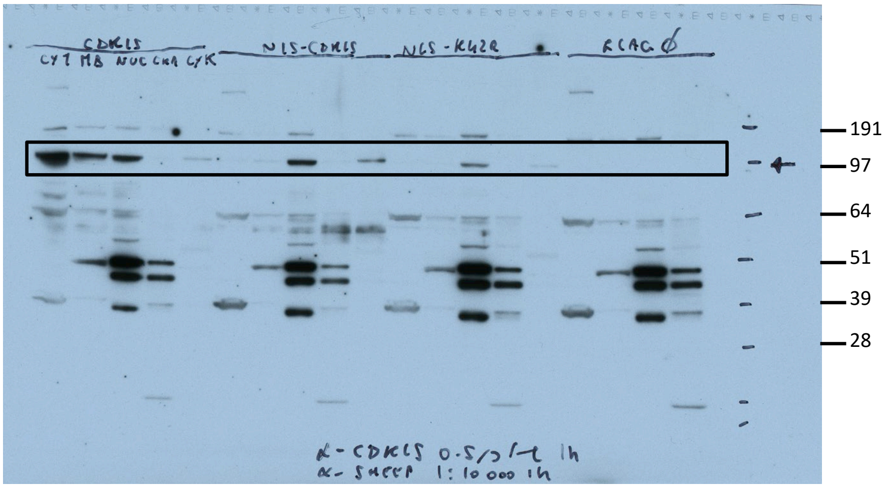

SMC1, GAPDH and H2B

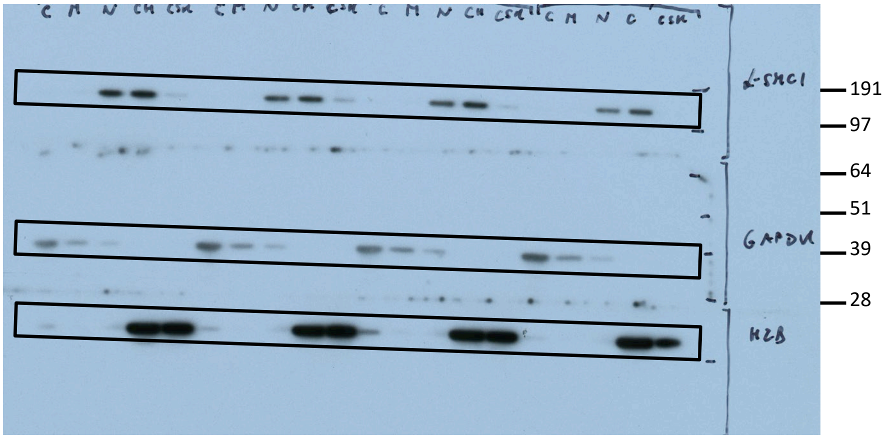

Figure EV2

MAP1S

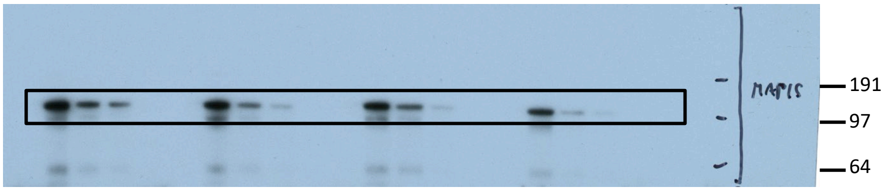

CEP131 and HDAC2

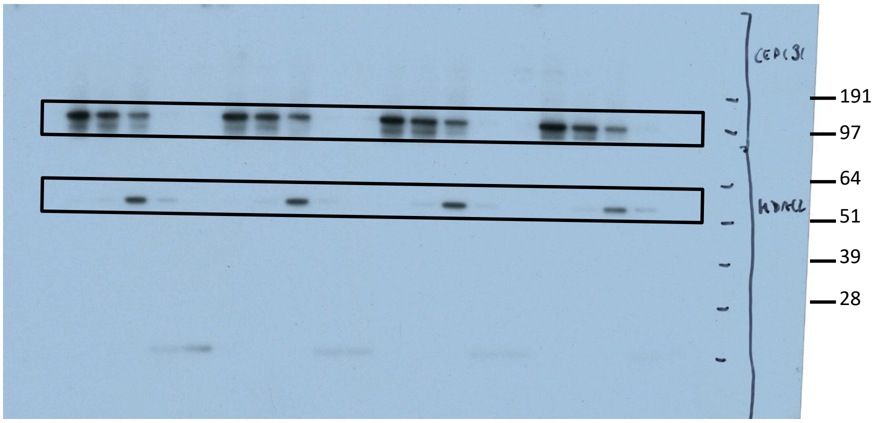

D.

PAR

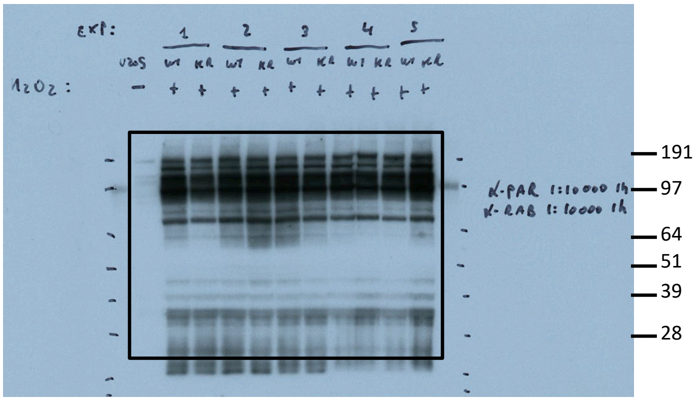

CDKL5

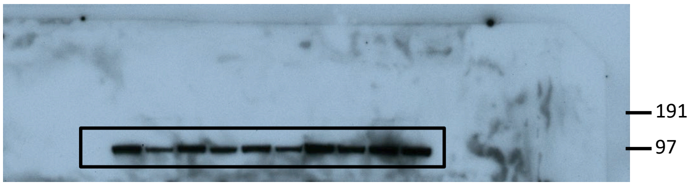

GAPDH

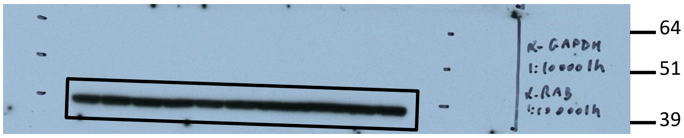

γH2AX

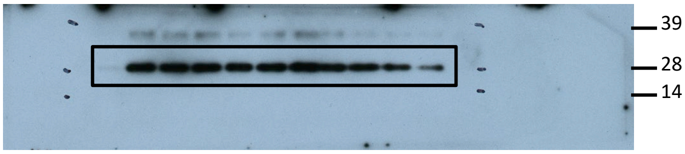

Supplement: Supplementary file 6 — Source Data for Expanded View [file EMBJ-40-e108271-s003.zip › EMBOJ-2021-108271R1-Figure_EV2_Source_Data-sd.zip!/Figure EV2/Source data Fig EV2A,B,D.pdf]
