# Supplementary material for: CDKL5 kinase controls transcription‐coupled responses to DNA damage
Source: EMBO J. 2021 Oct 4;40(23):e108271. doi: 10.15252/embj.2021108271 (PMC8634139; doi:10.15252/embj.2021108271)
Supplement: Supplementary file 6 — Source Data for Expanded View [file EMBJ-40-e108271-s003.zip › EMBOJ-2021-108271R1-Figure_EV2_Source_Data-sd.zip!/Figure EV2/Source data_Fig EV2_C_Omero figure links.docx]

| **Figure EV2C** | [OMERO.figure - Khanam et al. Fig EV2 C (dundee.ac.uk)](https://omero.lifesci.dundee.ac.uk/figure/file/381353/) |
| --- | --- |
